# Spatiotemporal transcriptomic mapping of regenerative inflammation in skeletal muscle reveals a dynamic multilayered tissue architecture

**DOI:** 10.1172/JCI173858

**Published:** 2024-08-27

**Authors:** Andreas Patsalos, Laszlo Halasz, Darby Oleksak, Xiaoyan Wei, Gergely Nagy, Petros Tzerpos, Thomas Conrad, David W. Hammers, H. Lee Sweeney, Laszlo Nagy

**Affiliations:** 1 Departments of Medicine and Biological Chemistry, Johns Hopkins University School of Medicine, Institute for Fundamental Biomedical Research, Johns Hopkins All Children’s Hospital, St. Petersburg, Florida, USA.; 2 Department of Biochemistry and Molecular Biology, Faculty of Medicine, University of Debrecen, Debrecen, Hungary.; 3 Max Delbrück Center for Molecular Medicine, Berlin, Germany.; 4 Myology Institute and Department of Pharmacology and Therapeutics, University of Florida, Gainesville, Florida, USA.

**Keywords:** Inflammation, Expression profiling, Macrophages, Skeletal muscle

## Abstract

Tissue regeneration is orchestrated by macrophages that clear damaged cells and promote regenerative inflammation. How macrophages spatially adapt and diversify their functions to support the architectural requirements of actively regenerating tissue remains unknown. In this study, we reconstructed the dynamic trajectories of myeloid cells isolated from acutely injured and early stage dystrophic muscles. We identified divergent subsets of monocytes/macrophages and DCs and validated markers (e.g., glycoprotein NMB [GPNMB]) and transcriptional regulators associated with defined functional states. In dystrophic muscle, specialized repair-associated subsets exhibited distinct macrophage diversity and reduced DC heterogeneity. Integrating spatial transcriptomics analyses with immunofluorescence uncovered the ordered distribution of subpopulations and multilayered regenerative inflammation zones (RIZs) where distinct macrophage subsets are organized in functional zones around damaged myofibers supporting all phases of regeneration. Importantly, intermittent glucocorticoid treatment disrupted the RIZs. Our findings suggest that macrophage subtypes mediated the development of the highly ordered architecture of regenerative tissues, unveiling the principles of the structured yet dynamic nature of regenerative inflammation supporting effective tissue repair.

## Abstract 2

Macrophages coordinate tissue repair by promoting regenerative inflammation. We discovered diverse myeloid cell subsets that organize in functional multilayered tissue zones, crucial for effective muscle repair.

## Graphical abstract

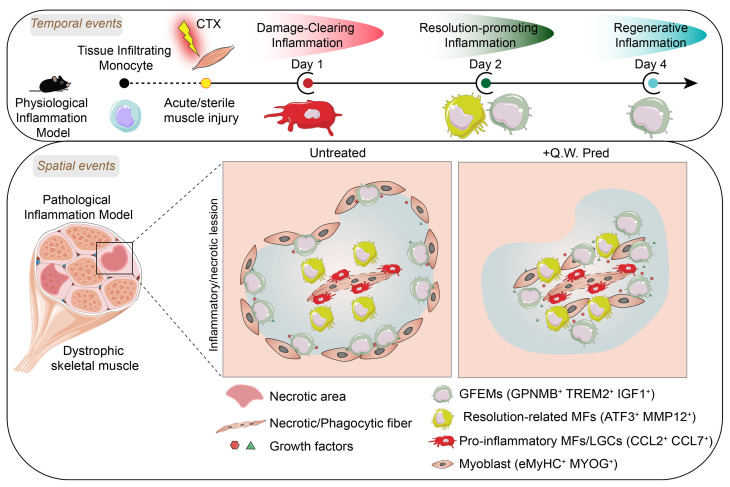

## Introduction

Muscle repair following injury involves a well-coordinated immune response, marked by the recruitment of myeloid cells, clearance of damaged tissue, activation of myofibroblasts, and formation of new blood vessels and extracellular matrix (ECM). Central to this process are macrophages (MFs), which regulate satellite cells to restore tissue integrity and function (1), but can also exacerbate disease progression in the context of asynchronous chronic inflammation (2–4). A delicate balance of immune modulation to promote the active resolution of inflammation and expression of growth factors (GFs) is important for emerging muscular dystrophy therapies (5, 6) and underscores the importance of a deep mechanistic understanding of (a) regenerating tissues’ histological complexity and (b) the spatiotemporal distribution, kinetics, interactions, and polarization states of innate immune cells in regeneration, a concept termed “regenerative inflammation” (1, 7–9).

In response to acute injury, circulating monocytes (Ly6C^hi^CCR2^+^CX3CR1^lo^F4/80^lo^) get activated, extravasate into the injured area, and differentiate into Ly6C^lo^F4/80^hi^CX3CR1^hi^ repair MFs (through an in situ phenotypic shift) capable of regulating all phases of regeneration (10). A major role of these muscle-infiltrating MFs is the clearance of cellular debris and the secretion of growth/differentiation factor 15 (GDF15), GDF3, IGF1, other GFs, and ECM proteins to coordinate muscle tissue repair through the regulation of myogenic, fibro/adipogenic, and endothelial cell fates (7, 11–13). Thus, the kinetics of regenerative inflammation are fundamental for the proper orchestration of myogenesis and the coordination of adjacent supportive repair-associated biological processes. Inhibition of the MF maturation/phenotypic shift pathways (including IGF1, MKP1/p38, SRB1/ERK, AMPK, C/EBPβ, STAT3, NFIX, BACH1/HMOX1, and PPARγ/RXR/GDF15) prevents the acquisition of the restorative phenotype and impairs regeneration (summarized in refs. 1, 8). Premature onset of the repair phenotype also impairs damage clearing (5, 14–18). Single-cell technologies and trajectory inference analyses now suggest that the monocyte/MF lineage may be more accurately interpreted as a hierarchical continuum of cell states upon tissue injury (19–25). We have identified and characterized 4 distinct and functional states of repair MFs observed during the repair phase in a myotoxin-induced injury model (7). However, deconvoluting their spatial arrangement and temporal progression through all phases of regeneration, as well as their contribution to tissue organization and function, remains a major challenge.

Here, we combined single-cell and spatially resolved transcriptomics complemented by immunofluorescence (IF) validation to deconvolute the events following muscle injury–induced monocyte infiltration.

## Results

### Highly ordered distribution of repair MF subtypes during muscle regeneration.

**Figure 1:**
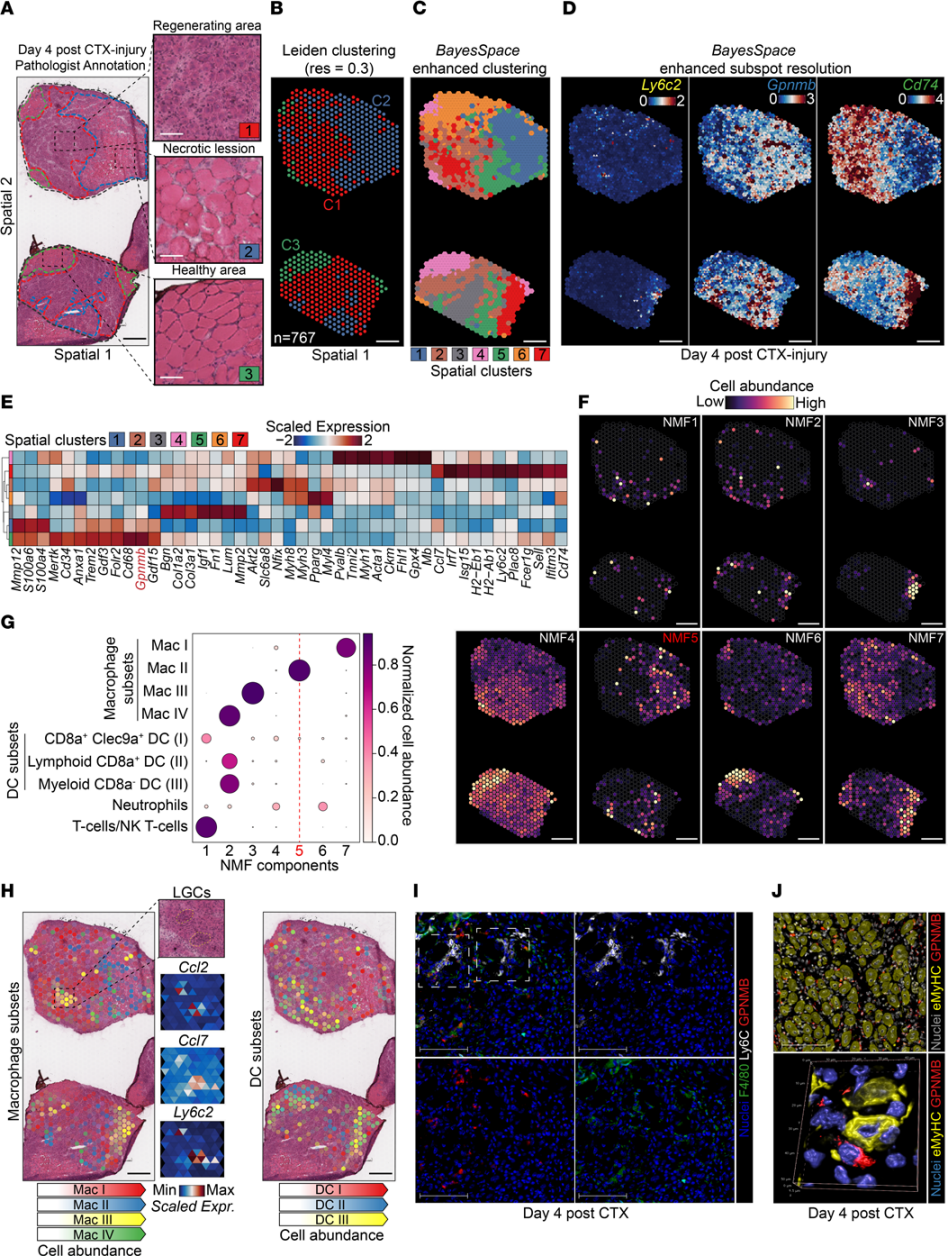
Spatial and single-cell transcriptomics integration and enhanced-resolution clustering resolve the cellular distribution of myeloid subtypes during regenerative inflammation. (**A**) H&E of TAs at 4 days after CTX injury used for ST. Insets indicate histopathological annotations (red-C1: regenerative muscle; blue-C2: necrotic/inflammatory lesions; green-C3: healthy muscle). C2 shows segmental necrosis of pale fibers with loss of cytoplasmic structures, active phagocytosis, C or delta lesions, and membrane damage (95), compared with C1, which includes inflammatory cells and regenerating myocytes. Scale bars: 50 μm. (**B**) Spatial clustering (Leiden algorithm; cluster resolution 0.3) identifies 3 discrete regions overlapping with histopathology annotations of **A**. The number of spots (*n* = 767) is indicated. (**C**) Enhanced subspot resolution clustering (BayesSpace) identified 7 spatial domains, not resolved at spot-level clustering. (**D**) Spatial expression patterns at subspot resolution of genes defining the myeloid subsets characterized by scRNA-Seq (7). Color scale shows log-normalized counts for each subspot. Gene label color corresponds to the classification in left panel of **H**. (**E**) Heatmap of the spatial expression of top predicted and curated markers, highlighting the specificity of the spatial BayesSpace clusters. *Gpnmb* is highlighted. (**F**) Identification of tissue compartments using NMF-based decomposition and day 4 after CTX reference immune subtype signatures (7). Spatial plots show cell abundance. (**G**) Dot plot of the estimated NMF weights of subtypes across 7 predicted NMF components. Note the differential abundance of MacII and MacIII subtypes and the overlap of DC subsets with MacIV. MacI, resolution-related MFs; MacII, GFEMs; MacIII, infiltrating monocytes/proinflammatory MFs; MacIV, antigen-presenting MFs (7). (**H**) Distribution and estimated cell abundance of MF and DC subtypes associated with specific NMF cellular compartments. Insets: histological area on NMF3, predicting MacIII and the formation of LGCs (encircled). The local spatial expression of known markers of LGCs (*Ccl2*, *Ccl7*) (58, 86) overlaps with the histological features and other MacIII markers (*Ly6c2*; **D**, *Plac8*; Supplemental Figure 1E). Scale bars: 500 μm. (**I**) IF detection of LGCs (MacIII) and GFEMs (MacII) by GPNMB, Ly6C, and F4/80 (green) in C57BL/6J animals at day 4 after CTX injury. Split channels are shown. White boxes indicate 2 LGC-like structures. Scale bars: 100 μm. (**J**) Upper: IF of GPNMB^+^ MFs and eMyHC^+^ fibers at day 4 after CTX injury. Lower: high-resolution volume projection confocal image of GPNMB^+^ MFs and eMyHC^+^ regenerating fibers preferential spatial proximity (3D reconstruction distances are indicated). Scale bar: 100 μm.

We have recently completed a comprehensive immune-specific (CD45^+^) single-cell transcriptomic analysis of the regeneration phase following acute sterile injury, complementing the wealth of data available (7, 19, 21, 22, 26, 27). Our focused single-cell RNA sequencing (scRNA-Seq) analyses provide an in-depth annotation of innate immune cell types, subpopulations, and states that dominate the early phases of tissue repair with higher resolution and better deconvoluted variability than generic non–cell type–specific scRNA-Seq approaches, resulting in the identification of 4 functionally distinct MF subtypes (7). Interestingly, we found that several GFs (i.e., IGF1, GDF3, GDF15) were enriched in a distinct repair MF subpopulation (GF-expressing MFs [GFEMs]). We were able to validate in vivo and refine the characterization of this repair MF subset by FACS using a predicted and highly enriched marker (i.e., glycoprotein NMB [GPNMB]) (7). We reasoned that a spatially resolved view of the regeneration phase is needed to reveal whether localization patterns of myeloid subsets exist and are predictive of their function. We used the spatial Transcriptomics (ST) Visium platform on tibialis anterior (TA) muscles at day 4 after cardiotoxin (CTX) injury (Figure 1, A and B, and Supplemental Figure 1A), which is highly dominated by repair-associated MFs (7), to gain insights into their spatial distribution during regeneration.

Two main workflows are used to identify molecular features that correlate with spatial location within a tissue: (a) performing differential gene expression (DGE) analysis based on spatially distinct clusters (i.e., preannotated pathological/anatomical regions within the tissue based on prior knowledge) and (b) finding features that have unique spatial patterning without taking clusters or spatial annotation into account (28). The first strategy using pathologist-annotated areas (see Figure 1A legend for designation criteria) exhibits clear spatial and morphological restriction at the tissue level (Figure 1, A and B). The 3 pathologist-predicted clusters correspond to areas of regenerative (cluster 1 [C1]), inflamed/necrotic (cluster 2 [C2]), and healthy (cluster 3 [C3]) muscle (Figure 1, A and B, and Supplemental Figure 1B) and correlate to the expression of relevant functional markers (Supplemental Figure 1, C and D) and Gene Ontology (GO) biological processes (Supplemental Figure 1E). Interestingly, *Gpnmb* (a marker of GFEMs) is predicted to be among the top 10 enriched genes in C2 (Supplemental Figure 1D), indicative of the GFEMs having unique spatial distribution surrounding necrotic areas. To explore the possibility that more detailed spatial coexpression/coherent histological patterns exist, we implemented BayesSpace (29), allowing inference of the spatial arrangement of subspots (Figure 1C) and enhancing the resolution of gene expression maps (Figure 1D and Supplemental Figure 1C). This resulted in the identification of 7 deconvoluted and spatially distinct tissue domains (Figure 1C and Supplemental Figure 1F). GO pathway enrichment analysis corresponding to each resolution-enhanced spatial cluster (Figure 1G) corroborated the existence of more complex histological and functional tissue domains during the regenerative inflammation phase compared with the pathologist annotations seen at spot-level resolution (Figure 1A). The existence of more discrete spatial tissue domains was further supported by the expression pattern of top spatially variable genes (Figure 1E).

Next, we integrated our spatial datasets with a high-resolution immune CD45^+^ scRNA-Seq dataset (>7,000 single cell profiles; day 4 after CTX) (7). This generated a reference to predict the abundance and spatial location of repair MF subsets (7) and other immune cell types in the Visium spots by applying a deconvolution method called Cell2location (30, 31). Similar to the established benefits of applying nonnegative matrix factorization (NMF) to conventional scRNA-Seq (32), the additive NMF decomposition generated 7 groups of spatial cell-type abundance profiles that captured colocalized cell types and subtypes (Figure 1F). The relative abundance and localization of muscle-infiltrating myeloid cells inferred by BayesSpace reflected a pattern of coordinated commitment, as shown by the antigen-presenting MF subset frequently colocalizing with 3 DC subsets, while other repair MF subsets had distinct distribution patterns (Figure 1, G and H). The spatial expression patterns of marker genes at subspot resolution for each of these subsets confirmed the NMF tissue components prediction (Supplemental Figure 1H), including GPMNB and GDF15 for GFEMs (MacII; Figure 1, D, I, and J, and Supplemental Figure 1, H–I) and CCL7, CCL2, and Ly6C2 for Langhans giant cells (LGCs) (LGCs appear as a component of MacIII classification; Figure 1, D, H [insets], and I). Interestingly, the MacI (resolution-related MFs) spatial distribution pattern (NMF7) appears to be surrounding areas occupied by LGCs/MacIII and suggests the formation of transcriptionally distinct zones near phagocytosed fibers (Figure 1, G and H, and Supplemental Figure 1H). In summary, the single-cell and ST integration coupled with enhanced subspot resolution clustering assigned the unique cellular distribution of MF and DC subtypes in specific/exclusive multicellular tissue zones during the regenerative inflammation phase.

### The sequential and temporal appearance of specialized MF subtypes.

Having established the utility of the combination of spatial and single-cell transcriptomics in a single time point, we next investigated it as a dynamic time course. Damage clearing and regenerative inflammation are characterized by the sequential phenotypic transitions of circulating monocytes to proinflammatory and then to repair MFs, a process correlated with tissue-regeneration kinetics. These reprogramming processes have been documented using several methodologies (5, 18, 33–38), but how the temporal appearance of repair MF subtypes defines the monocytic/MF continuum is unclear.

**Figure 2:**
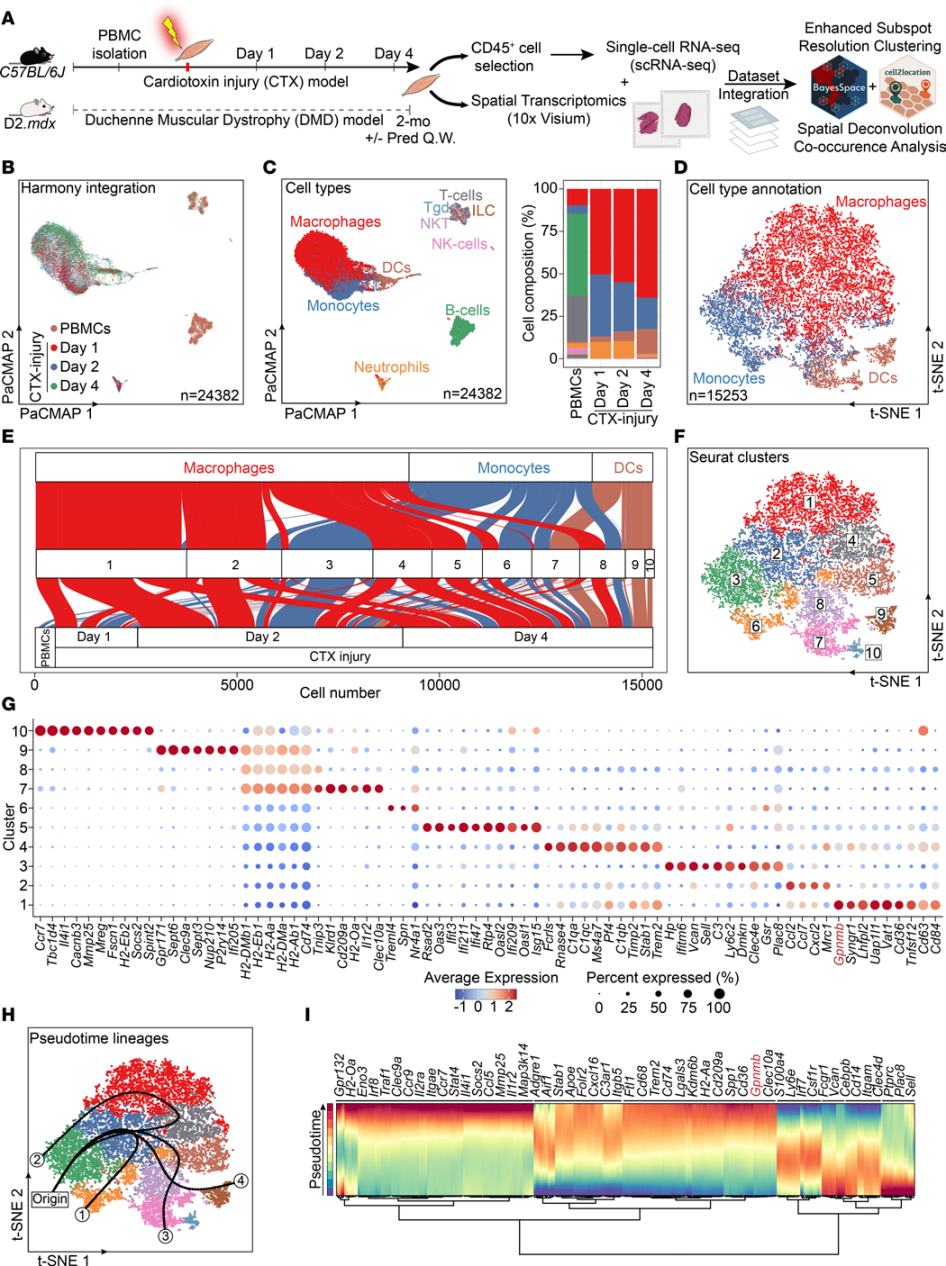
The sequential appearance of specialized MF subtypes orchestrates skeletal muscle regeneration. (**A**) Analysis workflow for CD45^+^ cell scRNA-Seq and ST (Visium) of regenerating and dystrophic muscle. Cell suspensions were collected from digested TAs of adult mice at 1, 2, and 4 days after CTX injury and steady-state GAST of 2-mo D2.*mdx*. PBMC datasets from noninjured C57BL/6J mice were from 10x Genomics. Enhanced spatial resolution, deconvolution, and cooccurrence of myeloid subtypes are achieved by single-cell and spatial dataset integration (BayesSpace and Cell2location). (**B**) Single-cell transcriptomes from CD45^+^ cells at days 1, 2, and 4 after CTX injury, and PBMCs were harmony integrated and batch-effect corrected. Data (24,382 cells) are presented as a PaCMAP projection and color coded by origin. (**C**) Integrated transcriptomic atlas of 10 major populations (SingleR automated cell type annotation ImmGen database) (96). Cell types are color coded. Right: cell-type proportions and compositional dynamics. MFs account for 41.2% of all immune cells. (**D**) Cells in the macro-clusters of interest (monocytes, MFs, and DCs) were reanalyzed in isolation. t-distributed stochastic neighbor embeddingg (t-SNE) visualization reveals local differences. Cells are colored by major cell-type classification. (**E**) Clustering of the isolated cell types from **D** resolved 10 subtypes of monocytes, MFs, and DCs. Subcluster composition (absolute numbers) is presented as an alluvial plot. (**F**) t-SNE visualization of subtypes of monocytes, MFs, and DCs. (**G**) Dot plot of top DEGs distinguishing the monocyte/MF/DC clusters (3 DC: clusters 7, 9 and 10; 2 monocytic: clusters 3 and 6; 2 MF subtypes: clusters 1, 4; and 3 MF transitional states: clusters 2, 5, and 8; **A**). Dot size represents the percentage of cells expressing each marker within a cluster. *Gpnmb*, the top GFEM marker (cluster 1) is highlighted in red. (**H**) t-SNE colored by cluster and inferred pseudotime (Slingshot; principal curves are smoothed representations of each lineage) with 4 predicted cell fates: 1 monocyte (patrolling monocytes), 1 MF (GFEMs), and 2 DC lineages. Origin determines the circulating Ly6C^hi^ monocyte population, projected at the start of all trajectories. Trajectory 1 predicts the patrolling monocyte differentiation (not relevant in injury) (37). (**I**) Gene expression dynamics of monocyte/MF/DC subpopulations resolved along latent time. Cells were subjected to trajectory inference using Monocle’s (97) differential expression analysis to identify lineages. Top likelihood-ranked genes by branch and pseudotime are shown. *Gpnmb* is highlighted.

**Figure 3:**
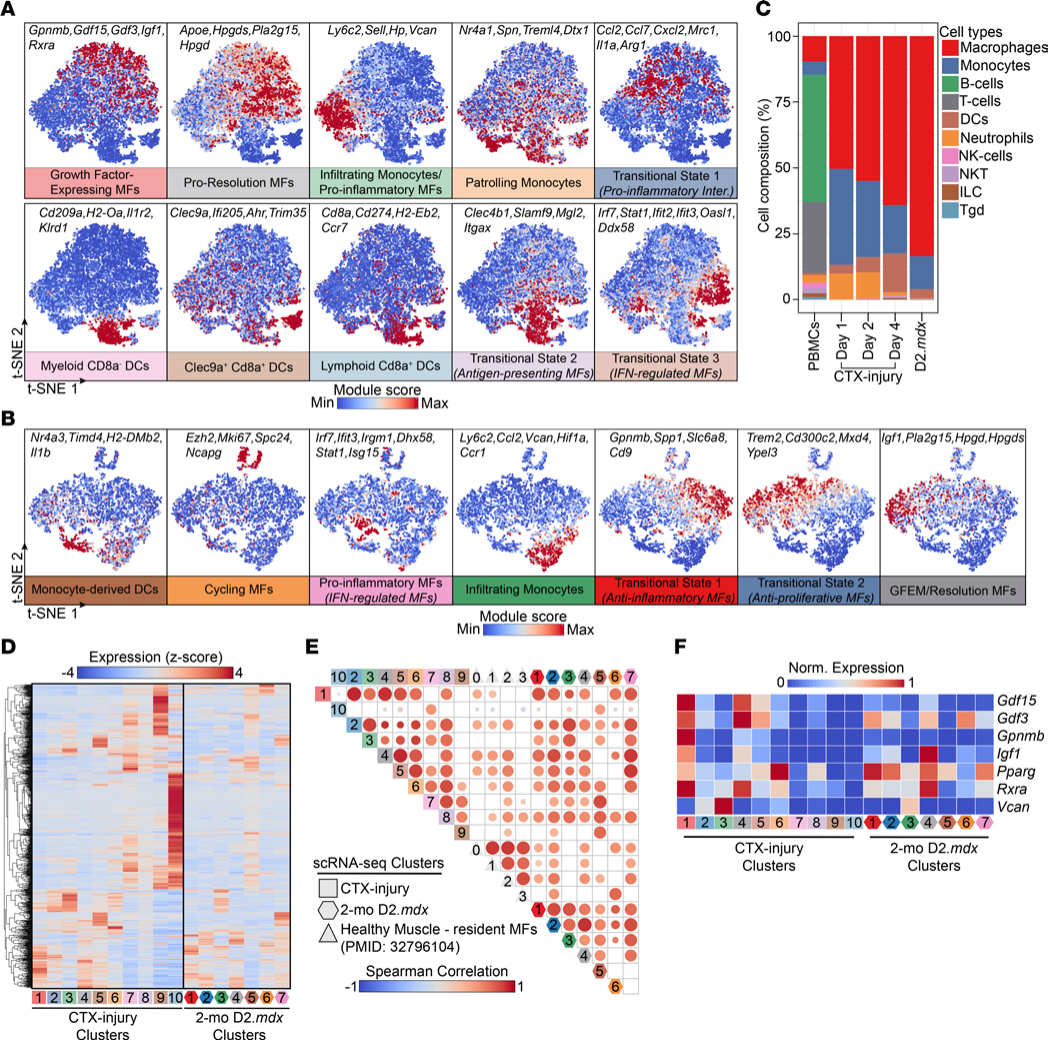
Infiltrating myeloid cell transcriptomic profile comparison from healthy, acutely injured, and early stage dystrophic muscle. (**A**) Module score of gene sets representing functional markers of each myeloid subset visualized with a t-SNE in the CTX injury time-course scRNA-Seq dataset. Genes for each module are indicated. (**B**) Module score of gene sets representing functional markers of each myeloid subset visualized with a t-SNE in the 2-mo D2.*mdx* scRNA-Seq dataset. Genes for each module are indicated. (**C**) Composition bar plot of the major immune cell types in the Harmony integrated dataset (2-mo D2.*mdx* + CTX injury). (**D**) Heatmap of top genes in 2-mo D2.*mdx* vs. CTX-injury monocytes, MF, and DC subsets. (**E**) Pairwise Spearman’s correlation plot of monocytes, MF, and DC subsets identified in 2-mo D2.*mdx*, CTX-injury, and resident muscle MFs from healthy quadriceps (36) (1,300 total; *presto* Wilcox AUC; logFC > 0.5, *P*-adj < 0.1, AUC > 0.5). The resident MFs are represented according to the following nomenclature chosen by the authors (36): cluster 0 (cluster 0), *Cd209* cluster (cluster 1), *Ccr2* cluster (cluster 2), and proliferating cluster (cluster 3). Color intensity and circle size are proportional to the correlation coefficients. Note (a) the relative uniqueness of resident MFs, (b) the high correlation of proinflammatory monocytes (cluster 3) in both CTX and D2.*mdx* datasets, and (c) the high correlation of cycling MFs in D2.*mdx* (cluster 6) with the resident MFs (proliferating cluster). (**F**) Heatmap of known regulators of regeneration in 2-mo D2.*mdx* versus CTX-injury monocyte, MF, and DC subsets.

To understand the MF subtype specification process, we generated an immune cell transcriptomic atlas using droplet-based single-cell 3′ RNA-Seq in CD45^+^ cells FACS isolated from CTX-injured TAs at days 1 and 2 and combined datasets from peripheral blood mononuclear cells (PBMCs) and day 4 after CTX (7) (Figure 2A). We used a compendium of software packages for scRNA-Seq data filtering and processing to eliminate dying cells, technical outliers, and doublets (Supplemental Figure 2A). The resulting Harmony-integrated (39) scRNA-Seq dataset contained 24,382 cells (Figure 2B). Next, we annotated the major cell types present in this dataset representing the entire immune cell milieu of the regeneration phase following injury (Figure 2C). Identification of cell types was based on the cluster-average expression of canonical genes included in the ImmGen consortium (40) and validated with a nested matrix of per-cell scores (Supplemental Figure 2B). As expected, the cumulatively largest and most ambiguous group of muscle-infiltrating cells are myeloid derived, most prominently monocytes and MFs (Figure 2C and Supplemental Figure 2C), classified by the expression of known MF markers such as F4/80 (*Adgre1*) and *Trem2* (Supplemental Figure 2, C and D). In addition, DGE in the major cell-type clusters provided additional context-dependent markers (Supplemental Figure 2C). This analysis revealed the dynamics of myeloid cell infiltration and differentiation with early detection of monocytes and a late appearance of DCs (Figure 2C). By focusing on ontogeny-linked populations such as monocytes, MFs, and DCs and analyzing them in isolation, we could further discriminate these closely related populations (Figure 2D). By using several frameworks, including subsampling cluster robustness metrics and clustering trees to guide the selection of single-cell clustering parameters (41), we were able to initially classify 9 distinct clusters, independent of their cell-type annotation origin (Supplemental Figure 2, E and F, and Supplemental Figure 3, A and B). However, considering the annotated cell-type origin (Figure 2D and Supplemental Figure 3C) and cluster stability index (Supplemental Figure 2F) prompted us to manually split cluster 3. The resulting 10 clusters (Figure 2, E and F) were then analyzed for unique markers using DGE between cell subpopulations within each cluster and all other cells in the dataset (Figure 2G). Both analyses revealed (a) 3 subsets of DCs, (b) 4 different subtypes of monocytes/MFs (including a nonrelevant in the context of muscle injury PBMC-originated patrolling Ly6C^lo^ monocyte population [ref. 37] enriched in cluster 6), and (c) 3 transitional states (intermediate phenotypes; clusters 2, 5 and 8) with varying cell number composition depending on the time point of isolation (Figure 2E) and unique gene expression profiles (Figure 2G and Figure 3A). We labeled DCs as (a) myeloid CD8a^–^ (cluster 7), (b) Clec9a^+^CD8a^+^ (cluster 9), and (c) lymphoid CD8a^+^ (cluster 10) (Figure 2G and Figure 3A) (7). We labeled MFs as GFEM (cluster 1), resolution related (cluster 4), and proinflammatory (cluster 3), in agreement with previous findings (7) (Figure 2G and Figure 3A). We marked the 3 MF transitional states as proinflammatory intermediate (cluster 2) due to the high-profile similarity to cluster 3, antigen presenting (cluster 8) due to the high expression of MHC genes, and IFN regulated (cluster 5) due to enriched interferon type I and II signature genes (Figure 2G and Figure 3A). To uncover the temporal subset dynamics during normal regeneration, we performed pseudotime mapping to infer continuous lineage structures (Slingshot, ref. 42; Figure 2H) and RNA velocity (dynamical modeling; scVelo; Supplemental Figure 3D) underlying differentiation processes (by calculating velocity length; Supplemental Figure 3E) and detecting putative driver genes (Figure 2I). Cluster 3 cells (representing the proinflammatory monocytes/MFs) appear first and give rise to 4 distinct MF and DC lineages. Among the novel and unique markers reported here (Figure 2G), GPNMB (also known as osteoactivin), a marker of the GFEMs (7), is also predicted to be among the drivers of the GFEM lineage (lineage 2; Figure 2, H and I). In summary, these findings (a) point toward one single progenitor cell type (infiltrating monocytes) as the origin of all myeloid (MF and DC) subsets accumulating following acute injury and during regeneration and (b) posit a central role for *Gpnmb* expression in the development of GFEMs.

### GPNMB is a marker and effector of GFEMs and a regulator of muscle regeneration.

**Figure 4:**
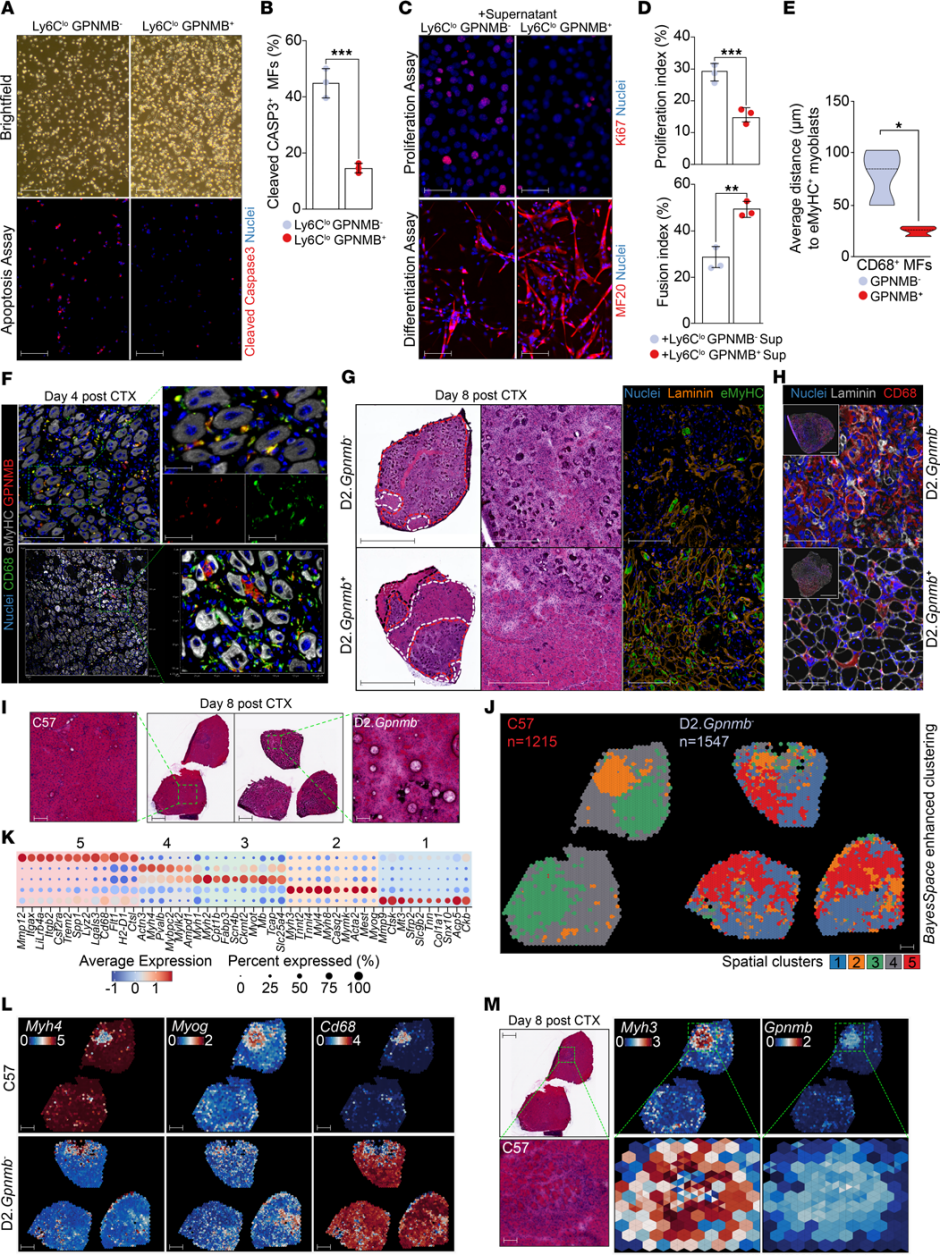
GPNMB is a marker and component of GFEMs, and its deficiency impairs regeneration. (**A**) Upper: Brightfield images of day 4 after CTX Ly6C^lo^F4/80^hi^GPNMB^–^ and Ly6C^lo^F4/80^hi^GPNMB^+^ muscle-infiltrating MFs ex vivo after 12 hours in culture (equal number of cells were seeded). Lower: apoptosis was assessed by cleaved caspase-3 immunostaining. Scale bars: 100 μm. (**B**) Percentage of apoptotic (CASP3^+^) Ly6C^lo^F4/80^hi^GPNMB^+^ and Ly6C^lo^F4/80^hi^GPNMB^–^ MFs (unpaired *t* test with a *P* = 0.0007; *n* = 3). (**C**) Effect of GPNMB^–^ and GPNMB^+^ MF-derived conditioned media on the proliferation and differentiation of C2C12 myoblasts. Scale bars: 100 μm. (**D**) Effect of GPNMB^–^ and GPNMB^+^ MF-derived conditioned media on C2C12 myoblasts (*n* = 3). Proliferation index as percentage of Ki67^+^ cells (*n* = 10 fields/group; unpaired *t* test, *P* = 0.0008). Fusion index as percentage of myotubes (visualized by heavy chain of myosin II) with more than 3 nuclei (*n* = 10 fields/experiment/group; unpaired *t* test, *P* = 0.002). (**E**) Spatial cell proximity quantification of GFEMs (CD68^+^GPNMB^+^) versus other MF subtypes to regenerating eMyHC^+^ fibers at day 4 after CTX in C57BL/6J animals (*n* = 3; >200 mm^2^ tissue area/sample; unpaired *t* test, *P* = 0.0267). (**F**) Detection of GFEMs by CD68 and GPNMB, in relation to eMyHC^+^ fibers in C57BL/6J animals at day 4 after CTX injury. Insets indicate the split channels. Scale bars: 100 μm (left); 20 μm (insets). Lower panel indicates high-resolution volume projection confocal images (3D reconstruction distances are shown). (**G**) Left: H&E images of D2.*Gpnmb*^–^ (KO) and D2.*Gpnmb*^+^ (WT) TAs at day 8 after CTX injury. Note the near complete absence of regenerating fibers (highlighted in white) and extensive inflammation areas (highlighted in red) in the D2.*Gpnmb*^–^. Right: IF detection of newly formed fibers by eMyHC in D2.*Gpnmb*^–^ and D2.*Gpnmb*^+^ TAs at day 8 after CTX injury. Scale bars: 1 mm (left H&E); 500 μm (right H&E); 100 μm (IF panels). (**H**) IF detection of mature MFs by CD68 in KO and WT at day 8 after CTX injury, correlating to the extent of unresolved inflammation. Scale bars: 100 μm (main); 1 mm (insets). (**I**) H&E images of regenerating TAs (day 8 after CTX injury) from WT (C57BL/6J) and D2.*Gpnmb*^–^ animals used for ST. Insets show magnified H&E areas (green rectangles). Scale bars: 500 μm (middle panels); 50 μm (far left and right panels). (**J**) Enhanced subspot resolution clustering of regenerating TAs (day 8 after CTX injury) from WT and D2.*Gpnmb*^–^ identified 5 spatial clusters (*n* spots/group are indicated). Scale bars: 500 μm. (**K**) Top marker gene expression after *z* score transformation for each spatial cluster. Dot size represents the percentage of subspots expressing the gene. (**L**) Spatial expression of representative healthy muscle (*Myh4*), differentiating myoblasts (*Myog*), and persistent inflammation/mature MF (*Cd68*) genes in WT and D2.*Gpnmb*^–^. Note the loss of the distinct structure of regenerative zones in the KO. Scale bars: 500 μm. (**M**) *Gpnmb* and *Myh3* spatial expression patterns in the C57BL/6J day 8 after CTX ST sample confirm the proximity of GPNMB^+^ MFs to early stage regenerating fibers and distinct tissue organization around lesions. Scale bars 500 μm (upper); 50 μm (lower). H&E previously presented in **I**. This duplication is intended to provide the location and context for the presented magnified feature plots. In all bar graphs, bars represent mean ± SD. *P < 0.05; **P < 0.01; ***P < 0.001.

In the CTX model, GPNMB expression can accurately predict the presence and abundance of GFEMs when gated for its cell surface expression on CD45^+^Ly6C^lo^F4/80^hi^ repair MFs at day 4 after CTX (Supplemental Figure 4A) (7). To evaluate the properties of these cells (CD45^+^Ly6C^lo^F4/80^hi^GPNMB^+^) and compare them with other repair MF subtypes (CD45^+^Ly6C^lo^F4/80^hi^GPNMB^–^), we sorted them from WT injured muscle (day 4 after CTX) and placed them in culture in equal numbers (Figure 4A). Twelve hours later, we evaluated their survival and activation of apoptosis pathways (assessed by cleaved caspase-3) (Figure 4, A and B). GPNMB^+^ MFs displayed prolonged survival compared with the GPNMB^–^ MFs (Figure 4, A and B), indicative of a likely more mature MF subtype (43). To assess paracrine effects, myoblasts were treated with MF supernatant and assayed for proliferation and fusion. GPNMB^+^Ly6C^lo^ MF supernatant promoted myoblast fusion, but had no effect on proliferation, while the GPNMB^–^Ly6C^lo^ MF-derived media had minimal effects in fusion but increased myoblast proliferation (Figure 4, C and D). These results support the role of GFEMs (GPNMB^+^Ly6C^lo^) as the sole repair-associated MF subset secreting GFs and supporting myoblast fusion. Importantly, these GPNMB^+^CD68^+^ MFs preferentially localize near (<20 μm) regenerating embryonic myosin heavy chain positive (eMyHC^+^) myofibers compared with other repair-associated MF subsets (CD68^+^GPNMB^–^; Figure 4, E and F), validating GPNMB as a functional marker of the GFEMs and potentially supporting their crosstalk with other regeneration-associated cells (44).

The DBA/2J strain carries a nonsense mutation in *Gpnmb* (R150X), effectively making these mice a global genetic *Gpnmb* KO (D2.*Gpnmb*^–^) (44–48). Tissue repair following acute injury is severely impaired in this model (44, 49–52), which we confirmed (Figure 4G). To test the modifying effect of GPNMB on muscle regeneration, we compared CTX-induced muscle repair in D2.*Gpnmb*^–^ and a coisogenic strain with a functional allele of *Gpnmb* (D2.*Gpnmb*^+^) (53). Eight days following CTX injury, we observed a significant increase in centrally nucleated fibers (Figure 4G and Supplemental Figure 4B), regeneration area (Figure 4G and Supplemental Figure 4C), and eMyHC^+^ fibers (Figure 4G and Supplemental Figure 4D), all indicative of a significant improvement in muscle repair, in the D2.*Gpnmb*^+^ mice. Concurrently, D2.*Gpnmb*^–^ CTX-injured muscles exhibited hyperinflammation (increased presence of CD68^+^ MFs) during the late recovery phase of regeneration (day 8 after CTX), which was significantly reduced/normalized in the D2.*Gpnmb*^+^ mice (Figure 4H and Supplemental Figure 4E). These findings suggest additional autocrine roles of GPNMB in regulating the resolution of inflammation (48). This observation was further supported by (a) quantifying the total number of CD45^+^ muscle-infiltrating cells, DCs, and neutrophils during the time course of regeneration (Supplemental Figure 4F), (b) the observed asynchrony in the MF phenotype transition during the regenerative inflammation phase (day 4 after CTX) using established repair MF maturation markers (Supplemental Figure 4G) (35), (c) the increased presence of F4/80^+^CD163^+^ and F4/80^+^CD206^+^ resolution-related MFs at day 8 after CTX (Supplemental Figure 4, H and I), and (d) the utilization of ST in regenerating muscles from WT (C57BL/6J) and D2.*Gpnmb*^–^ during the late recovery phase (day 8 after CTX injury; Figure 4I and Supplemental Figure 4J). While using C57BL/6 mice as controls in this ST experiment has the limitation of inherent differences in inflammatory responses between these 2 strains (54), its use provides a reference for proper and complete regeneration in a background compatible with the scRNA-Seq datasets. Our integrated ST analysis of a total of 2,762 spots passing our filtering criteria revealed minimal overlap between samples even after sample batch correction (Supplemental Figure 4K). Our enhanced clustering analysis at subspot resolution suggests the presence of 5 spatial clusters (Figure 4J and Supplemental Figure 4L) and revealed major differences in inflammation- and regeneration-related clusters, 2 of which were overwhelmingly enriched in the D2.*Gpnmb*^–^ samples (clusters 1 and 5; Figure 4J). Predictably, markers of mature MFs (*Adgre1* and *S100a4*) were enriched in the D2.*Gpnmb*^–^ ST samples (Supplemental Figure 4, M and N). Global tissue-wide pseudobulk analysis of differentially expressed genes (DEGs) (Supplemental Figure 4N) and marker analysis of the identified spatial clusters (Figure 4K) revealed that the D2.*Gpnmb*^–^ muscles are characterized by the downregulation of healthy muscle fiber genes (e.g., *Myh4*, *Pvalb*, *Actn2*, *Myh1*, *Myl1*, *Ttn*) and overexpression of inflammation-related genes (e.g., *Spp1*, *Lyz2*, *Cd68*, *Mmp9*, *Mmp12*, *Lgals3*, *Ctsk*, *Fcer1g*, *S100a4*; Figure 4L and Supplemental Figure 4N). In addition, the overlap of the spatial expression patterns of *Gpnmb* and *Myh3* in WT regenerating muscle supports our finding that GFEMs preferentially colocalize with regenerating fibers (Figure 4M). Integrating the scRNA-Seq dataset from the CTX time course (Figure 2F) to spatially map the different MF and DC subtypes on these muscles using NMF (Supplemental Figure 4O) revealed that GFEMs’ (NMF4) and antigen-presenting subtypes’ (including DCs; NMF3) abundance is markedly reduced in the D2.*Gpnmb*^–^ muscles (Supplemental Figure 4P). Their distribution is apparent in small regenerating spots compared with the tissue-wide spread distribution seen in normally regenerating WT muscles (Supplemental Figure 4P). Overall, these results support the unbiased identification of GPNMB as both a marker and an effector of GFEMs.

### High-resolution ST view of dystrophic muscle and associated MF subtypes.

**Figure 5:**
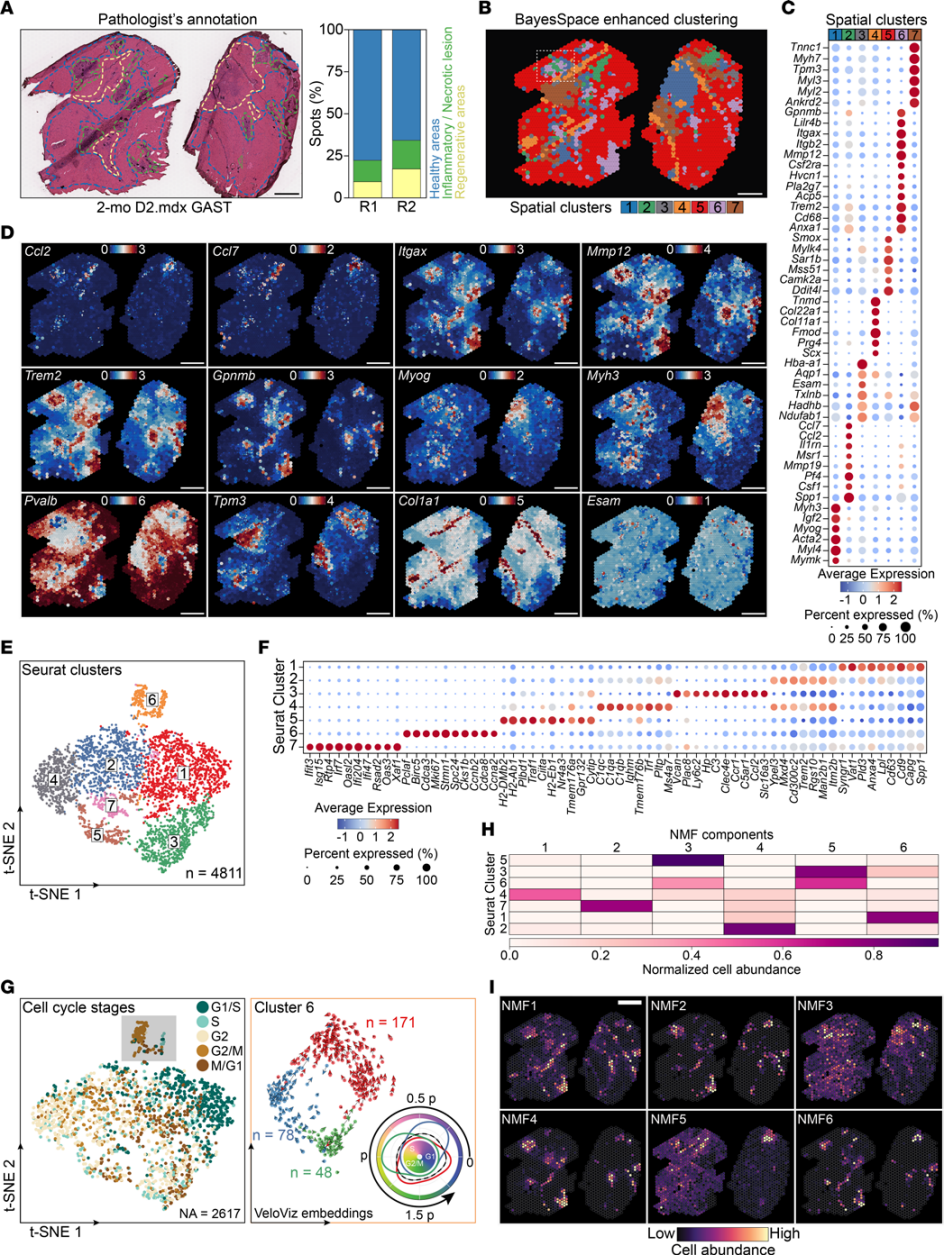
scRNA-Seq and ST integration with enhanced-resolution clustering resolve the complex dystrophic muscle architecture and cellular distribution and profiles of myeloid subtypes. (**A**) Left: H&E images of mouse GAST from 2-mo D2*.mdx* used for ST. Histopathological annotation areas are noted: regenerative muscle (yellow), necrotic/inflammatory lesions (green), healthy muscle (blue). Right: Percentage of spots in the annotated areas. (**B**) Enhanced subspot resolution clustering (BayesSpace) identified 7 spatial clusters (color coded), which were not resolved by pathologist annotations. The white rectangle highlights a lesion with structured inflammation and regeneration zones. (**C**) Top marker gene expression after *z* score transformation for each spatial cluster. Dot size represents the percentage of subspots expressing the gene. (**D**) Spatial expression of representative genes coding for markers of each spatial cluster: *Ccl2* and *Ccl7* (LGCs: cluster 2*)*, *Itgax*, *Mmp12*, *Trem2*, and *Gpnmb* (resolution-related MFs and GFEMs: cluster 6), *Myog* and *Myh3* (newly regenerating fibers: cluster 1), *Pvalb* and *Tpm3* (healthy muscle: cluster 7), *Col1a1* (ECM: cluster 4), and *Esam* (endothelial cell/vasculature-enriched areas: cluster 3). Note the differential spatial expression patterns in the highlighted region of **B**. (**E**) Single-cell transcriptomes derived from CD45^+^ cells from 2-mo D2*.mdx* GAST. A total number of 4,811 myeloid cells (MFs, monocytes, DCs; SingleR automated annotation using the ImmGen database) were analyzed. Data are presented as a t-SNE projection to visualize variation in single-cell transcriptomes. The subsampling-based clustering approach (chooseR) resolved 7 myeloid subsets (color coded). (**F**) Top marker genes for the 7 identified clusters. Dot size represents the percentage of expressing cells within a group, and color scale represents the average expression level (row *z* score) across all cells within the cluster. (**G**) Left: 2D embeddings visualizing cell cycle phases of the 2-mo D2*.mdx* scRNA-Seq dataset using t-SNE. NA indicates the number of unassigned cells. Right: 2D embeddings visualizing 3 subclusters of cycling cells from parent cluster 6 using VeloViz (98) embeddings. Cell numbers for each subcluster are indicated. Arrows show velocity projections (velocyto.R). (**H**) Identification of tissue compartments using NMF-based decomposition and 2-mo D2*.mdx* reference immune subtype expression signatures (7). Heatmap of the estimated NMF weights of subtypes (rows) across 6 predicted NMF components (columns), corresponding to the identified cellular compartments. Relative weights normalized across domains for every MF subtype are shown. (**I**) Spatial plots show cell abundance for each immune cell subtype calculated in **H**. Scale bars: 500 μm.

Given the highly ordered cellular organization of skeletal muscle and the importance of intercellular communication in Duchenne muscular dystrophy (DMD) progression, we reasoned that a spatially resolved view of disease-driven gene expression changes will reveal the relevant myeloid subpopulations and enable comparisons to the ones found in physiological injury/repair. We used a workflow combining ST with the immune-based (CD45^+^) scRNA-Seq approach described above (Figure 2A) to obtain spatial gene expression measurements of D2.*mdx* (a model of DMD) (55) skeletal muscle (gastrocnemius [GAST]) at a DMD disease stage with seemingly intact regeneration capacity (2 months of age) but still carrying a *Gpnmb* nonsense mutation (Figure 5A). Our ST analysis of 1,509 spots passing our filtering criteria revealed a gradient in the detected number of genes per spot (Supplemental Figure 5A). These differences are consistent between integrated biological replicates (Supplemental Figure 5, A and B) and overlap with pathologist-annotated areas of necrotic/inflammatory lesions and regenerative and healthy tissue (Figure 5A). Enhanced subspot resolution clustering revealed additional spatial domains beyond what the pathologist could annotate based on H&E staining (Figure 5A) for a total of 7 spatial clusters (Figure 5B and Supplemental Figure 5C). Marker enrichment analysis for these clusters revealed 2 distinct inflammatory clusters (clusters 2 and 6), 2 clusters indicating healthy muscle fibers (clusters 5 and 7), 1 regenerative cluster (cluster 1), 1 ECM-related cluster (cluster 4), and 1 endothelial cell/angiogenesis-related cluster (cluster 3; Figure 5, C and D).

The matching scRNA-Seq profiles of sorted CD45^+^ cells (2-month-old D2.*mdx*) allowed us to classify 7 subsets comprising monocytic, MF, and DC lineages (Figure 5E and Supplemental Figure 5D). These clusters were then analyzed for specific markers using DGE between cells within each cluster and all other cells in the dataset (Figure 5F), resulting in 1 subset of DCs (cluster 5), 3 different subtypes of MFs (clusters 4, 6, and 7), and 2 transitional states (intermediate phenotypes; clusters 1 and 2) (Figure 5E and Figure 3B). To uncover the temporal dynamics in the maturation of these dystrophy-associated subsets, we performed pseudotime mapping by RNA velocity (Supplemental Figure 5E), assessed the rate of differentiation by calculating velocity length (Supplemental Figure 5F), and detected putative driver genes (Supplemental Figure 5G). Integrating the scRNA-Seq profiles into the ST dataset predicted 6 NMF spatial tissue domains where these myeloid subsets localize (Figure 5, H and I). This integrative analysis yielded valuable insight into the dystrophic environment regarding the specialized myeloid subpopulations.

### Cycling MFs are enriched in dystrophic muscle and present in human DMD.

The scRNA-Seq analysis and marker prediction of the D2.*mdx* model identified a distinct proliferating MF subset in cluster 6 (Figure 5, E–G). These cycling MFs appear as a mature divergent lineage at the end of the pseudotime trajectory, suggestive of a tissue-resident enriched subset (Supplemental Figure 5G). To confirm the proliferative capacity of these MFs, we bioinformatically mapped the cell-cycle stages of each cell in the scRNA-Seq dataset (Figure 5G) and then stained acutely injured and dystrophic muscles for Ki67 (Supplemental Figure 5, H–J). We confirmed the enrichment of the cycling MF subset only in the dystrophic muscle (Supplemental Figure 5, H–J). Interestingly, the cycling MF subset is mapped on NMF 3 and 5 along with the DC and infiltrating monocytes, respectively (Figure 5, H and I). In contrast, the rest of the myeloid subsets are uniquely assigned to separate NMF domains (Figure 5, H and I).

To validate these findings, we reanalyzed publicly available single-nuclei RNA-Seq (snRNA-Seq) datasets from vastus lateralis biopsies collected from healthy and DMD patients (Supplemental Figure 3F) (27). We integrated all samples, batch corrected the datasets, annotated all cell types, isolated the myeloid subsets (monocyte, MFs, DCs) for downstream analysis (Supplemental Figure 3G), predicted 5 clusters (Supplemental Figure 3G), and determined the cell-cycle stage of each cell (Supplemental Figure 3H). Markers such as *PLK1* and *IGF1* confirmed the enrichment of a cycling myeloid subset (cluster 4) and the presence of GFEMs (cluster 3) in human DMD muscles, respectively (Supplemental Figure 3, I and J).

### Comparative analysis of myeloid subpopulations in uninjured muscle, physiological regeneration, and dystrophy.

We investigated similarities and differences in the myeloid cell type and subtype composition among (a) healthy muscle (tissue resident) (36), (b) physiological regeneration–associated, and (c) dystrophy-associated cells (Figure 3). Similarities between acutely injured and dystrophic muscle include MFs being the dominating myeloid cell type in both conditions and the presence of at least 1 subtype of DCs (Figure 3C). However, the ratio and heterogeneity of the DC populations are substantially reduced in dystrophic muscle; only 1 predicted monocyte-derived DC subset was observed (Figure 3, B–D). A strong correlation regarding gene expression exists between the DMD-enriched cycling (D2.*mdx* cluster 6; Figure 5E and Figure 3B) and tissue-resident MFs from uninjured muscle (tissue-resident cluster 3; Figure 3E). Concurrently, the GFEMs and resolution-related subtypes converge into a single population with mixed expression profiles and multiple intermediate phenotypes in dystrophic muscles (D2.*mdx* cluster 4; Figure 5E and Figure 3, B, D, and E). This discrepancy in marker (i.e., *GPNMB* and *IGF1*) expression distribution is also apparent in the human single nuclei DMD datasets (Supplemental Figure 3, I and J). Additional differences can be observed in the magnitude of expression of known regulators and effectors of MF-mediated regeneration (Figure 3F) (7, 12). This suggests that even in the early stages of chronically injured muscles, the subset diversity and compositional dynamics of regenerative inflammation (including the intermediate/transitional phenotypes) are altered.

### Damage-clearing and regenerative inflammation form multilayered tissue zones that are sensitive to glucocorticoids.

**Figure 6:**
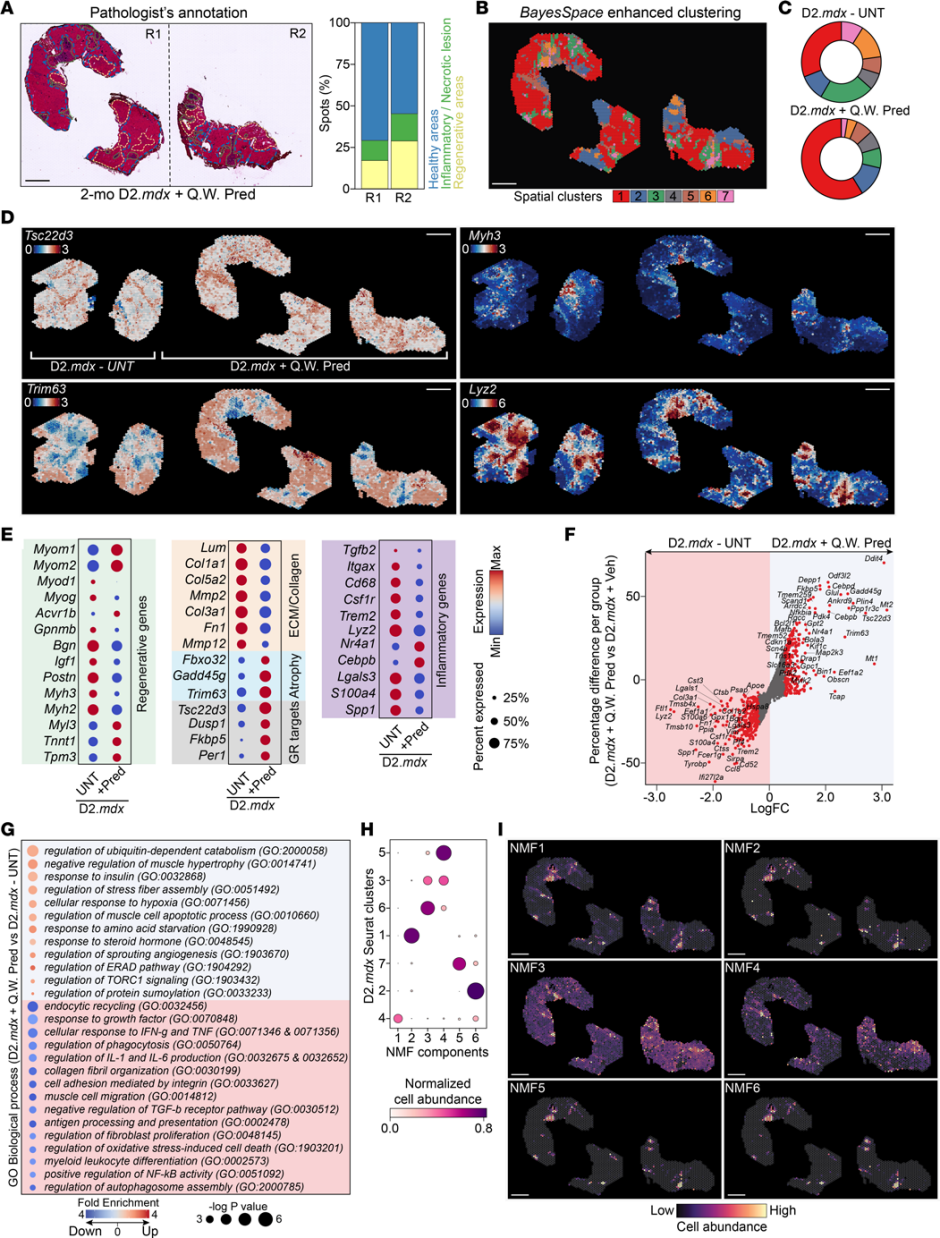
ST organization of dystrophic muscle upon intermittent Pred treatment. (**A**) Left: H&E images of GAST from 2-mo D2*.mdx* mice treated weekly (Q.W.) for 4 weeks with Pred, used for ST. Histopathological annotation areas: regenerative muscle (yellow), necrotic/inflammatory lesions (green), healthy muscle (blue). Right: Percentage of spots in annotated areas. Each section is from a different biological replicate, and each library was obtained from a separate Visium experiment followed by bioinformatic integration to remove batch effects. (**B**) Enhanced subspot resolution clustering (BayesSpace) identified 7 meaningful spatial clusters (color coded) unresolved by pathologist annotations. (**C**) Comparison of the spatial cluster (color coded) subspot composition in untreated D2.*mdx* and D2.*mdx* treated Q.W. with Pred. UNT, untreated. (**D**) Spatial expression of representative glucocorticoid receptor (GR) targets, regenerative muscle, inflammation, and atrophy marker genes is shown. *Myh3* indicates newly regenerating fibers, *Tsc22d3* GR target engagement, *Lyz2* inflammatory myeloid cells, and *Trim63* atrophy-inducing pathways. (**E**) Representative DEGs in untreated D2.*mdx* versus D2.*mdx*+Q.W. Pred comparison, grouped in functional categories. Dot size represents the percentage of spots within a treatment group. (**F**) Unbiased global tissue-wide DGE of all spots in D2.*mdx*+Q.W. Pred versus D2.*mdx*-UNT (red dots indicate significant DEGs; *P* < 0.05, logFC > 1). Top DEG names are indicated. (**G**) GO pathway enrichment analysis of the DEGs in D2.*mdx*+Q.W. Pred versus D2.*mdx-*UNT ST datasets. Top significant up- and downregulated pathways are shown (*P* < 0.001, fold enrichment > 2). Gray box, enriched GO terms with Pred treatment; red box, downregulated terms with Pred-treatment. (**H**) Identification of tissue compartments in D2.*mdx*+Q.W. Pred-treated ST samples using NMF-based decomposition and 2-mo D2*.mdx* reference immune subtype expression signatures (7). Dot plot of estimated NMF weights of cell subtypes (rows) across 6 predicted NMF components (columns) corresponding to the identified cellular domains. Relative weights normalized across components for every MF subtype are shown. (**I**) Spatial plots show cell abundance for each immune cell type calculated in **H**. Scale bars: 1 mm.

Glucocorticoids (GCs) are used to treat DMD to delay loss of ambulation (56). Prolonged daily use, however, has detrimental side effects, including bone demineralization, Cushingoid, cardiomyopathy (57), and muscle atrophy (58, 59). Intermittent dosing has emerged as a potentially less harmful dosing regimen (58). Therefore, the effect of prednisolone (Pred) on regenerative inflammation zone (RIZ) architecture and MF subtype specification was assessed using a 4-week intermittent dosing regimen (5 mg/kg Pred once weekly) in 4-week-old D2.*mdx* mice (Supplemental Figure 6A). The expression of over 2,000 genes per spot was mapped on the Pred-treated GAST muscles using ST (Figure 6, A and B), with 7 distinct spatial clusters (Figure 6B and Supplemental Figure 6C) and markers (Supplemental Figure 6E) deconvoluted at subspot resolution. By integrating the ST data from nontreated dystrophic muscles (Supplemental Figure 6D), we compared the impact of Pred treatment on spot distribution and cluster composition (Figure 6C). More specifically, we observed increased spatial expression of GC receptor targets (*Tsc22d3*, *Dusp1*, *Fkbp5*, *Per1*) and atrophy-associated genes (*Fbxo32*, *Gadd45g*, *Trim63*; Figure 6, D and E). These findings suggest that atrogene expression occurs regardless of GC dosing frequency. Additionally, weekly Pred treatment resulted in a significant decrease in the expression of inflammatory (e.g., *Lyz2*, *Spp1*, *S100a4*, *Lgals3*, *Cd68*, *Trem2*), ECM (e.g., *Col1a1*, *Lum*, *Col3a1*, *Fn1*, *Fbn1*), and regenerative genes (e.g., *Igf1*, *Postn*, *Myh3*, *Myog*, *Myod1*, *Myl4*; Figure 6, D and E, and Supplemental Figure 6F). These cluster-specific data are supported by global spot-level pseudobulk gene expression differences (Figure 6F; tissue-wide top DEGs) and gene enrichment GO pathway analysis (Figure 6G) and are consistent with prior data (58). Mapping the MF subtypes isolated from untreated dystrophic muscles (Figure 5E) to the Pred-treated muscle using NMF and Cell2location’s spatial mapping model also reveals a similar pattern of distribution around necrotic and regenerative areas, but a substantial reduction in GFEM/resolution-related cell abundance (Figure 6, H and I; cluster 4).

**Figure 7:**
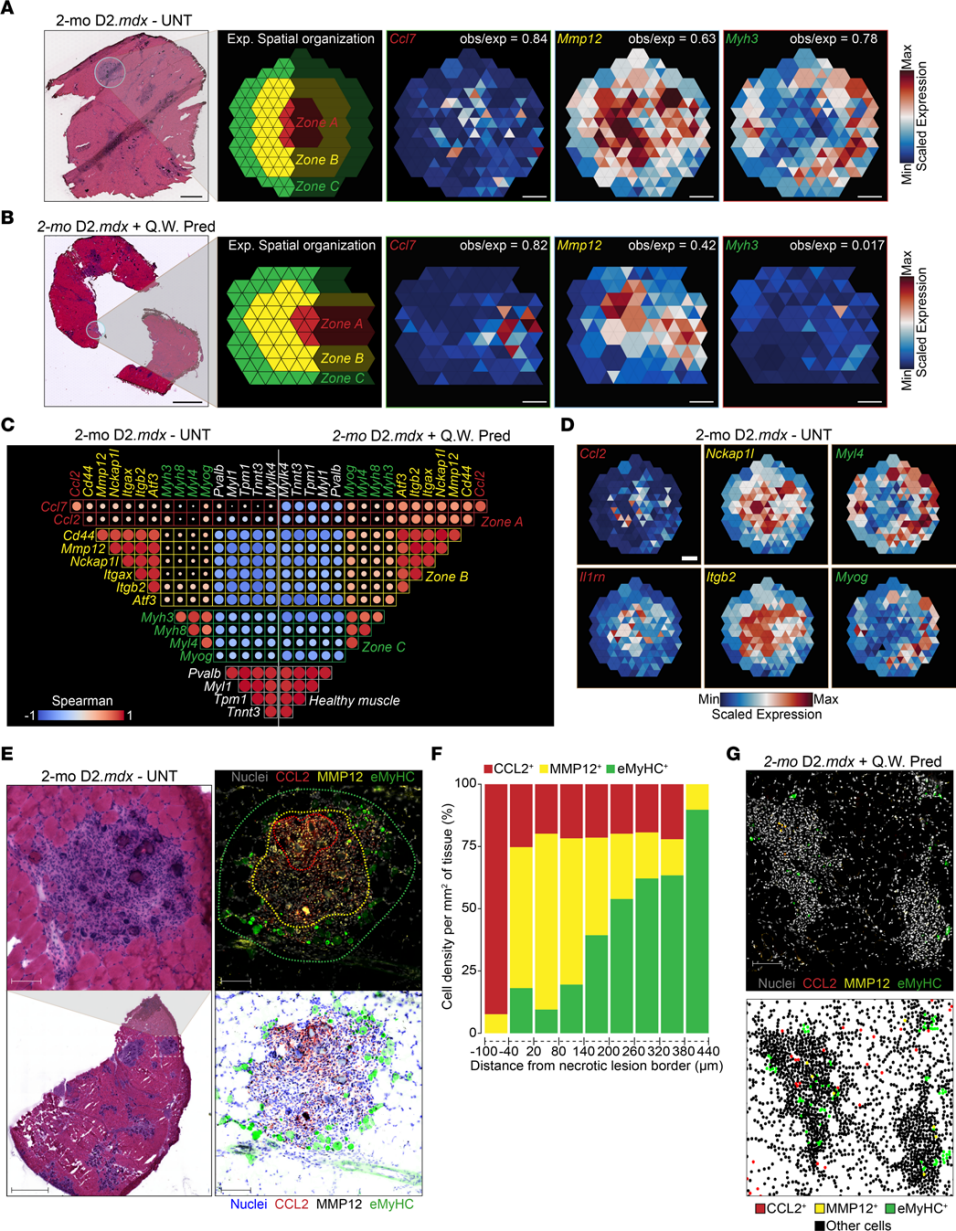
RIZs are disrupted by GC treatment in early stage dystrophy. (**A**) Magnified view of representative structures and RIZs in untreated 2-mo D2.*mdx* muscles. Zone A represents the center of an inflammatory lesion occupied by LGCs (Ccl7^+^); zone B is occupied by a gradient of resolution-related MFs (Mmp12^+^) and GFEMs; and zone C represents the regeneration zone marked by developing myofibers (*Myh3*^+^). The correlation of observed/expected zone organization is quantified per subspot. H&E data have been previously presented in Figure 4A and provide the location and context for the magnified feature plots. (**B**) Abnormal tissue zones in 2-mo D2.*mdx*+Q.W. Pred animals. Note the disintegration/absence of regenerating fibers (*Myh3*^+^) in zone C. H&E images have been previously presented in **A** and provide the location and context for the magnified feature plots. (**C**) Global subspot correlation (Spearman’s) of spatial gene expression in 2-mo D2.*mdx*-UNT and 2-mo D2.*mdx*+Q.W. Pred samples. Higher correlation in Pred samples suggests a collapse and rearrangement of inflammatory (zones A, B) and regenerative (zone C) zones (color coded). (**D**) Example of RIZs formed with alternative markers. Scale bars: 500 μm (left panel,**A**, H&E); 1 mm (left panel, **B**, H&E); 100 μm (right panel, **A**); 50 μm (right panel, **B**); 100 μm (**D**). (**E**) Representative H&E region of RIZs in 2-mo D2.*mdx* GAST validated by IF. The MF subtypes and zones were visualized with IF (bottom panel shows the absorbed signal) for CCL2 (zone A), MMP12 (zone B), and eMyHC (zone C). Dotted lines indicate the zones and interface layer (red: necrotic lesion) selected for cell density quantification in **F**. Scale bars: 1 mm (lower left); 100 μm (others). (**F**) Stacked bar histogram of CCL2^+^, MMP12^+^, and eMyHC^+^ cell density inside (–1 to –100 μm) and outside (+1 to +440 μm) the necrotic boundary in **E**. (**G**) Representative IF region with 2 inflammatory lesions in 2-mo D2.*mdx*+Q.W. Pred GAST samples. MF subtypes and regenerating fibers were visualized as in **E**. Bottom panel indicates the cell density and distribution. Scale bar: 100 μm.

These findings prompted us to inspect the necrotic/inflammatory and regenerating areas in both untreated and Pred-treated dystrophic muscle. A magnified view of these ST areas in untreated dystrophic muscle revealed layered structures and cell distribution (Figure 7A). According to the MF subtype distribution and predicted markers, these zones have (a) proinflammatory MFs forming LGCs (60) in the center of lesions around necrotic/phagocytic fibers (zone A), (b) a layer of resolution-related MFs and GFEMs creating a cell barrier between the inflammatory lesion and the healthy/regenerating areas (zone B), and (c) newly regenerating fibers occupying the periphery of the lesion, likely receiving the growth signals from the GFEMs (zone C; Figure 7A). In contrast, in Pred-treated muscles, the outer layer representing the regenerating fibers (zone C) is absent (Figure 7B). To select specific subspots and quantify these zones and cellular distribution in our ST data, we developed an R Shiny (https://github.com/hlszlaszlo/SpatialZoneR) application that compares a histologically defined RIZ model to the observed expression of regenerating fibers (e.g., *Myh3*, *Myog*, *Myl4*) and MF subtype-specific markers (e.g., *Ccl7*, *Ccl2*, *Il1rn*, *Mmp12*, *Nckap1l*, *Itgb2*, *Atf3*). This quantification validated the expected layered RIZ model in early stage dystrophic muscle (observed/expected ratio = 0.78; Figure 7A) and the collapse of zone C with Pred treatment (obs/exp ratio = 0.017; Figure 7B). Global tissue-wide subspot analysis of the RIZs revealed minimal spatial correlation of zone- and cell type–specific markers mapped in degenerative and regenerative foci of untreated muscles (Figure 7C), suggesting RIZs have distinct spatial architecture. This negligible spatial expression overlap could be further recapitulated by individual inspection of additional ST-predicted RIZ-specific markers (*Ccl2*, *Il1rn*, *Nckap1l*, *Itgb2*, *Myl4*, *Myog*; Figure 7, C and D). Importantly, a significantly higher pairwise spatial correlation between zone-specific markers in Pred-treated muscles was evident, indicating the loss of the expected spatial distinction of these structures (Figure 7C). Next, we validated the existence and spatial architecture of the RIZs in adult 2-mo D2.*mdx* untreated muscle (Figure 7E and Supplemental Figure 7A) and the effect of Pred treatment (Figure 7G and Supplemental Figure 7C) by IF and calculated the cell densities and average distances extending up to 500 μm from the boundary of a given necrotic lesion (Figure 7F, Supplemental Figure 6G, and Supplemental Figure 7B). Furthermore, we expanded the panel of antibodies to include other ST-predicted MF subtype markers that can be used to detect RIZs in dystrophic lesions (Supplemental Figure 7D). Overall, these data demonstrate that Pred treatment diminishes the muscle’s capacity to regenerate extensive lesions and causes the disorganization of zone-specific markers (Figure 7G, Supplemental Figure 6G, and Supplemental Figure 7C), in agreement with the ST (Figure 7, B and C). These findings suggest that GCs destabilize RIZs (overlap of zones A/B and loss of zone C), which may be the tissue architectural cause of hindered regeneration.

To confirm the presence of RIZs on an independent dataset, we reanalyzed available ST datasets from early stage dystrophic muscles (61, 62) using our enhanced subspot resolution and clustering BayesSpace workflow. Our ST analysis of mouse GAST tissue from young 6-week-old D2.*mdx* untreated animals validated the presence of RIZs and provided additional insights into the molecular landscape of dystrophic muscle (Supplemental Figure 8). More specifically, histological examination delineated distinct zones of tissue pathology (Supplemental Figure 8A), as described previously in our 2-mo D2.*mdx* ST samples (Figure 5A), while advanced subspot resolution clustering identified an equal number of 7 spatial clusters correlating with various states of muscle inflammation and structured regeneration (Supplemental Figure 8B). Gene expression profiling within these clusters validated the clustering and suggested cellular activity: *Pvalb* marked the quiescent state of healthy muscle in cluster 7, while *Csf1*, *Ly6c2*, and *Ccl7* were characteristic of neutrophils and monocyte-derived LGCs within cluster 2, indicative of a specialized proinflammatory/phagocytic response. *Mmp12*, *Atf3*, and *Nckap1l* expression identified GFEMs and mature MFs engaged in tissue resolution within cluster 6, and *Myh8* and *Myl4* marked the emergence of regenerating muscle fibers in cluster 1 (Supplemental Figure 8C). A closer examination of specific lesions revealed that the RIZ organization and architecture (Supplemental Figure 8D) were consistent with spatial patterns previously characterized in our study (Figure 7, A and C–E, and Supplemental Figure 7, A and B). Interestingly, analysis of prominent inflamed regions in these younger (6-week-old) animal samples unveiled unique spatial clustering indicative of a recent injury, with an absence of clusters representing the advanced regeneration stages and a predominance of phagocytes and necrotic fibers (Supplemental Figure 8E). These findings suggest an ongoing response to recently inflicted focal damage, which is observed by the increase in marker-specific IF signal intensity in these regions (Supplemental Figure 7E). Overall, these findings provide additional support for the existence of MF-organized RIZs, which are responsive to pharmacological interventions.

### The GFEM transcriptional program depends on ATF3.

**Figure 8:**
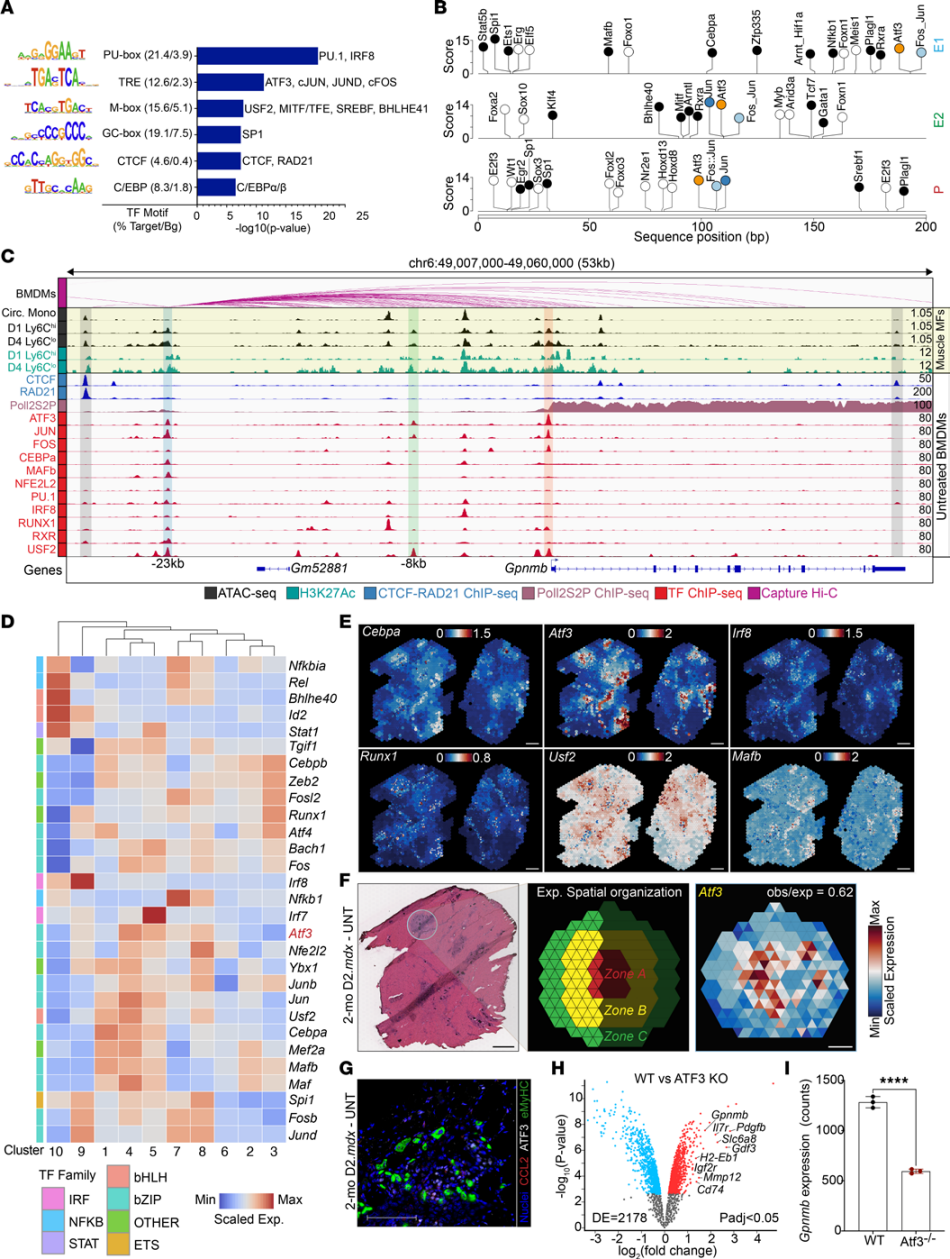
ATF3 directly regulates a GFEM-like transcriptional program. (**A**) De novo motif enrichments around the TSSs of GFEM-associated genes. ATAC-Seq peaks of day 4 after CTX Ly6C^lo^ repair muscle MFs within 50 kb of TSSs were selected as input. Detected motif matrices, *P* values, and background are shown. (**B**) Predicted scores and motif map of 2 distal enhancers (E1, E2) and 1 proximal (P) site around the *Gpnmb* locus, selected based on ATAC-Seq. Open and closed circles indicate the absence or presence of corresponding TF mRNA, respectively. Motifs of ATF3 and JUN are highlighted. (**C**) Genome browser view of the *Gpnmb* locus depicting capture Hi-C (in naive BMDMs), ATAC-Seq (blood monocyte and muscle-infiltrating MFs; normalized scale), and ChIP-Seq (in naive BMDMs and muscle-infiltrating MFs) for indicated TFs, active transcription histone marks (H3K27Ac), and elongating polymerase II (S2P). CTCF and RAD21-defined transcriptional unit boundaries, distal (E1, green; E2, blue) and proximal (P, red) *Gpnmb*-associated regulatory elements and track scales are indicated. (**D**) Heatmap of the highest expressed TFs (decile filtered) in the myeloid subtypes of the CTX scRNA-Seq dataset (Figure 2F). Hierarchical clustering and average log-normalized expression values are shown. *Atf3* is highlighted. (**E**) Spatial expression feature plots of top TFs with detected binding in the regulatory elements in the D2.*mdx* samples. (**F**) Magnified view of *Atf3* spatial expression in representative RIZs in untreated 2-mo D2.*mdx* muscles. The correlation of observed/expected zone organization is quantified per subspot for each zone and indicates an overlap of *Atf3* with zone B. Data (left and middle) have been previously presented in Figure 4A and Figure 7A, respectively, and provide the location and context for the magnified feature plot and expected spatial organization. Scale bars: 500 μm (**E**, left panel, **F**). (**G**) IF region of a lesion in 2-mo D2.*mdx* GAST muscle. MF subtypes were visualized with CCL2 (red, zone A) and ATF3 (yellow, zone B), and regenerating fibers with eMyHC (green, zone C). Scale bar: 100 μm. (**H**) Volcano plot showing the DEGs in the *Atf3*^–/–^ naive BMDMs (*P* < 0.01, FDR < 0.01). Number of DEGs and gene labels of GFEM-predicted markers among top DEGs are shown. (**I**) *Atf3* mRNA expression in WT and *Atf3*^–/–^ naive BMDMs (*n* = 3; unpaired *t* test, *P* < 0.0001).

Finally, we sought to identify the potential transcription factor(s) (TFs) driving the gene networks of GFEMs. De novo motif analysis using accessible chromatin regions revealed distinct motif matrices (i.e., PU-box, TRE, M-box, C/EBP) with expected (i.e., PU.1, JUN, FOS; lineage determining factors) (18) and unexpected (i.e., ATF3 and USF2) TF binding enrichment around transcription start sites (TSSs) of GFEM-linked markers (Figure 8A). While *Gpnmb* is a documented target of microphthalmia-associated transcription factor (MITF)/TFE transcription factor E (TFE) across various cell types, including myeloid cells (63–65), close examination by capture Hi-C and predictive modeling of TF interaction with distal and proximal regulatory elements of the *Gpnmb* locus suggests activating transcription factor 3 (ATF3) playing a central role in its regulation (Figure 8, B and C). This was evidenced by enriched motif scores (Figure 8B), clear demarcation of ATF3 and its cofactor JUN binding at muscle MF-specific chromatin accessible sites (Figure 8C), and consistent alignment of ATF3 binding with other markers of transcriptional activity, including active histone modification (H3K27Ac) and elongating RNA polymerase II (Figure 8C). Notably, the regulatory domain architecture defined by 2 chromatin structure regulators, namely the genome-wide insulator CTCF and the cohesin ring subunit RAD21, coincided with the positioning of strong ATF3 binding sites (Figure 8C). In parallel, the transcriptional landscape of myeloid cell subtypes depicted in the CTX-based scRNA-Seq dataset substantiated our prediction by the unbiased inclusion of ATF3 in the highest tier of expressed TFs (Figure 8D). ATF3’s spatial gene (Figure 7C, Figure 8, E and F, and Supplemental Figure 8, D and E) and protein (Figure 8G and Supplemental Figure 7F) expression patterns in D2.*mdx* muscle suggests a targeted response within RIZs (surrounding LGCs and overlapping with resolution-related MFs). Furthermore, comparative mRNA expression analysis in WT and *Atf3^–/–^* bone marrow–derived MFs (BMDMs) (a) underscores ATF3’s direct influence on basal GFEM-like marker gene regulation (Figure 8H) and (b) bolsters ATF3’s central regulatory role in *Gpnmb* expression (Figure 8I). These findings align with reports on ATF3’s regulatory functions within innate immune cells (66–70). Overall, in our extensive epigenomic investigation of primary MFs, integrating capture Hi-C, ATAC-Seq, and ChIP-Seq, we identified the TF ATF3 as a direct regulator of a gene expression module resembling that of a GFEM-like transcriptional network.

## Discussion

The spatiotemporal ordering of molecular events that drive regenerative inflammation and MF subtype specification in physiological repair and dystrophy remains unclear. Inherent limitations of current gene expression profiling technologies, such as low throughput or lack of spatial context, impede the understanding of how different subtypes interact in space to coordinate regeneration and, importantly, how immune asynchrony affects DMD progression. In this study, we (a) carried out a comprehensive high-dimensional transcriptomic analysis of MF subpopulations in acute and chronic injury models, (b) mapped the spatial distribution of the MF subsets, (c) validated a predicted GFEM marker (GPNMB) and its impact in regeneration, (d) identified ATF3 as a key TF in *Gpnmb* expression and GFEM regulation, (e) discovered a disease-associated subset that is enriched and present in human DMD pathology, and (f) evaluated the impact of intermittent GC treatment on the newly identified multilayered RIZs. Our computational analyses increased ST resolution and, by estimating myeloid subtype compositions at each location, linked the organization of injured and regenerating muscle at different time points to various histomorphological regions of the complex dystrophic muscle.

Recent studies have presented transcriptomic atlases of regenerating and dystrophic muscle, especially highlighting parenchymal and progenitor cells (19, 22, 26, 27, 71–73). Here, we present the analyses of comprehensive immune-specific single-cell and ST datasets of regenerating muscle after acute injury and during dystrophy, providing deep spatial annotation of myeloid subpopulations with enhanced subspot resolution. This approach enables us to understand changes in monocytic/MF/DC states based on spatial distribution/proximity and their transcriptional and regulatory variations. Additionally, our scRNA-Seq analysis offers a trajectory perspective on myeloid cell types, highlighting early monocyte infiltration as the main source of progenitors. The lack of proliferative signatures in CTX injury–related MFs and negligible naive monocyte profiles beyond the acute injury phase indicates their limited role in later reparative stages. Moreover, the fact that the expression of early monocyte markers such as *Vcan* is limited to the first 2 days after injury is consistent with the temporally restricted nature of monocyte infiltration. While the trajectories of MF markers *Aif1*, *Adgre1*, and *Csf1r* (74) align with early MF activation stages, they do not maintain peak expression at the end of differentiation. This suggests an earlier role for CSF1 signaling in MF subtype specification and supports a model where reparative MFs derive from early infiltrating monocytes rather than continuous monocyte infiltration throughout regeneration.

Analysis of the scRNA-Seq time course identified different states and subtypes of myeloid cells, including ones associated with spatial distribution and disease conditions. Leveraging our integrated data, we inferred and identified potential regulators of MF subtypes. The identification of GPNMB as a GFEM marker (7) and effector of regeneration has important ramifications for the D2.*mdx* DMD model (55). In other models of acute injury, GPNMB^+^CD68^+^ MFs are also found during the recovery phase and have been suggested as regulating the balance between fibrosis and fibrolysis (49), possibly contributing to the accelerated fibrotic DMD disease progression observed in the D2.*mdx* model. In parallel, unresolved inflammatory gene expression and the prolonged presence of antiinflammatory CD206^+^ and CD163^+^ MFs with increased antigen-presenting capacity in the absence of functional GPNMB is in line with an additional autocrine role of GPNMB in regulating the resolution of sterile inflammation and pointing toward a protective role in muscle injury by modulating the polarization of MFs (44, 48, 51, 75, 76). Interestingly, the cleaved ectodomain of GPNMB may also act as a paracrine GF, influencing the behavior of nearby cells and participating in the complex interplay of signaling required for regeneration (44, 77–80). Future studies should aim to delineate the specific, context-dependent functional contributions of both the membrane-bound and soluble forms of GPNMB.

In the context of dystrophy, the identification of an enriched mature cycling MF subset is intriguing. The predicted regulators of this subset that involve ECM-depositing genes suggest a bona fide fibrotic subset that will establish the later stages of DMD disease progression. A more likely scenario supported by the profile comparison with tissue-resident MFs in uninjured muscle (36) indicates the enrichment of a muscle-resident locally proliferating subset that expands to sufficient numbers to uniquely cluster. This is probably dependent on the disease stage at the time of muscle isolation, and thus, longitudinal studies are warranted to validate this observation. A well-conserved mature MF subset was also found between acutely (cluster 5) and chronically injured (cluster 7) muscles with strong interferon type I signatures and could reflect the enrichment of muscle-resident MFs (36). The exact function of this subset is not clear, but it may be similar to the function of regenerative bystander DCs that confer protection from subsequent infections (81). Finally, our findings stemming from the comparison of the CTX injury and D2.*mdx*-specific subsets highlight how the preexisting inflammatory environment within dystrophic tissues gradually alters regenerative inflammation and, ultimately, the quality of regeneration.

Interestingly, our ST data reveal a distinct niche demarking the zone surrounding the injured fibers with a border that doesn’t appear random between injured, regenerating, and uninjured tissue, marked by *Atf3*, *Mmp12*, *Pla2g7*, *Itgb2*, *Lilrb4*, *Hvcn1*, and *Nckap1l* expression, among others (spatial cluster 6; Figure 5C, Figure 7, A–D, Supplemental Figure 7, and Supplemental Figure 8). These markers of zone B are indicative of several MF-driven mechanisms that act simultaneously to guide zone formation. For example, we have previously shown that lipid mediator changes support MF subtype transitions during muscle regeneration (5). The phospholipase A2 group VII (PLA2G7) is secreted by MFs and can degrade platelet-activating factor and generate lysophosphatidylcholine to resolve inflammation by modulating MF polarization (82, 83). Increased metalloprotease-12 (MMP12) and leukocyte immunoglobulin-like receptor B4 (LILRB4) are shown to function as an immune checkpoint and regulate/limit MF activation, differentiation, and polarization during pathogenesis (84).

The formation of the multilayered zones is possibly directed by cell polarity, which is necessary for leukocytes to mediate inflammation and immune responses (85). In tissue injury, migrating cells generate distinct actin assemblies at the front and the back to maintain the physical separation of inflammatory signals. This coordinated control of asymmetric morphology and regulatory signals represents an important form of cell polarity. Cells can polarize directionally in response to subtle spatial cues (e.g., gradients of extracellular chemoattractants) (86). This self-organizing process is mediated by localized scaffolded protein complexes of regulatory proteins such as NCK-associated protein 1 like (NCKAP1L), predicted in zone B, to help confine the damage-clearing zone from the regenerative zone (87). It’s possible that NCKAP1L and other predicted proteins, such as hydrogen voltage-gated channel 1 (HVCN1), are required for MF cell polarity and chemotaxis for the proper formation of the multilayered RIZs. In parallel, MFs undergo plasma membrane fusion to form multinucleated cells such as the LGCs, typically found during active inflammatory processes (88). In inflamed muscle, LGCs have been shown to preferentially express CCL7 (60), and we found CCL7 to mark the damage-clearing inflammation zone (zone A). How multinucleation per se contributes to the functional specialization of mature mononuclear MFs remains unclear. It’s possible that cell-cell fusion and multinucleation function to confer LGC-specific activity.

Our findings have important ramifications for DMD therapeutic development, as current approaches seek to replace dystrophin with truncated microdystrophins (89). While protective of the muscle, these strategies will likely require small molecule adjuncts to address preexisting pathology, cardiotoxicity, and immune activation (90, 91). Additionally, the de novo expression of dystrophin-based therapies might not benefit advanced-stage DMD patients where the muscle is already replaced by connective tissue. Tailoring immune-modulating treatments that promote muscle regeneration and resolve unwanted inflammation is one strategy that may overcome some of these issues. Findings of this work include the discovery of distinct damage-clearing and RIZs in early stage dystrophic muscle resembling the temporal and synchronous MF subtype specification of physiological repair. Interestingly, the RIZs are sensitive even to intermittent GCs. By employing advanced imaging and high-dimensional data analysis, RIZs can be identified and quantified in the context of DMD antiinflammatory therapies (92–94). Overall, the identification of these dynamic and targetable structures provides opportunities to evaluate disease and impaired regeneration states, assess current therapeutic approaches, and develop strategies to enhance RIZs and associated cell types to preserve these highly ordered architectural foci of regeneration in DMD and likely in other tissues and in other chronic inflammatory diseases.

## Methods

### Sex as a biological variable.

We exclusively examined male mice because DMD is an X-linked disease.

### Statistics.

ANOVA with Bonferroni’s correction for multiple testing was used to determine statistical significance. Adjusted *P* values are stated within figure legends. All experiments were performed using at least 3 independent experiments from each sample group. For FACS, at least 4 independent samples were analyzed, and at least 5 × 10^5^ cells were counted for each population. For histology, at least 10 samples were used. In bar graphs, individual data points are shown, and the error bars represent the SD. Student’s *t* tests and ANOVA analyses were performed in GraphPad Prism 10 with 95% CIs, and *P* < 0.05 was considered statistically significant.

### Study approval.

All animal experiments were carried out in accordance with ethical regulations and approved by the IACUCs at Johns Hopkins University (license no. MO21C391) and the University of Florida (protocol 202011094).

### Data availability.

The day 4 post-CTX injury scRNA-Seq dataset is available in the NCBI’s Gene Expression Omnibus database (GEO GSE161467). Day 1 and day 2 post-CTX injury, D2.*mdx* scRNA-Seq data, and Visium ST datasets are under GEO GSE223813. Mouse PBMCs are from the 10X Genomics repository (SC3_v3_NextGem_DI_CellPlex_Mouse_PBMC_10K_Multiplex). Human vastus lateralis snRNA-Seq data are under BioProject PRJNA772047. ATF3 KO BMDM gene expression microarray data are from GEO GSE44034. TF ChIP-Seq data include cFOS (SRR2353461, SRR2353465, GSM1875488, GSM1875492), NFE2L2 (SRR3714085, GSM2212311), MAFB (SRR2976081, GSM1964739), PU.1 (SRR3407110), CEBPa (SRR4302495), IRF8 (SRR4302496), RUNX1 (SRR4302498), cJUN (SRR6660224, SRR6660225, GSM2974662, GSM2974663), USF2 (SRR6660274, GSM2974712), ATF3 (SRR10486877, GSM4174754), RXR (SRR25923453), CTCF (SRR1514109, GSM2867715), RAD21 (SRR5937719, GSM3164905), Poll2pS2 (SRR6247019, GSM2845631), and H3K27Ac (GSE262945). ATAC-Seq data are under GEO GSE129393 and GSE262945. cHi-C data are in GEO GSE276450. Values for all data points in graphs are reported in the Supporting Data Values file.

### Extended material and methods.

Reagents, assays, protocols, and bioinformatic analysis workflows are detailed in the Supplemental Methods.

## Author contributions

AP, DO, XW, PT, and DWH conducted the experiments. AP and LH designed the figures. AP, LH, and GN performed the computational analyses. AP and LN planned the project, TC, DWH, and HLS provided resources and histological samples, and LN supervised the work. AP and LN drafted, and AP, DWH, and LN revised the manuscript. All authors discussed the results and commented on the manuscript.

## Supplementary Material

Supplemental data

Supporting data values

## Version 1 08/27/2024

In-Press Preview

## Version 2 10/15/2024

Electronic publication

## References

[1] Patsalos A (2021). Myeloid cell diversification during regenerative inflammation: Lessons from skeletal muscle. Semin Cell Dev Biol.

[2] Juban G (2018). AMPK activation regulates LTBP4-dependent TGF-β1 secretion by pro-inflammatory macrophages and controls fibrosis in duchenne muscular dystrophy. Cell Rep.

[3] Theret M (2022). Macrophages in skeletal muscle dystrophies, an entangled partner. J Neuromuscul Dis.

[4] Villalta SA (2009). Shifts in macrophage phenotypes and macrophage competition for arginine metabolism affect the severity of muscle pathology in muscular dystrophy. Hum Mol Genet.

[5] Giannakis N (2019). Dynamic changes to lipid mediators support transitions among macrophage subtypes during muscle regeneration. Nat Immunol.

[6] Markworth JF (2020). Resolvin D1 supports skeletal myofiber regeneration via actions on myeloid and muscle stem cells. JCI Insight.

[7] Patsalos A (2022). A growth factor-expressing macrophage subpopulation orchestrates regenerative inflammation via GDF-15. J Exp Med.

[8] Caballero-Sanchez N (2022). Regenerative inflammation: When immune cells help to re-build tissues. FEBS J.

[9] Karin M, Clevers H (2016). Reparative inflammation takes charge of tissue regeneration. Nature.

[10] Chazaud B (2020). Inflammation and skeletal muscle regeneration: leave it to the macrophages!. Trends Immunol.

[11] Joe AW (2010). Muscle injury activates resident fibro/adipogenic progenitors that facilitate myogenesis. Nat Cell Biol.

[12] Varga T (2016). Macrophage PPARγ, a lipid activated transcription factor controls the growth factor GDF3 and skeletal muscle regeneration. Immunity.

[13] Wosczyna MN, Rando TA (2018). A muscle stem cell support group: coordinated cellular responses in muscle regeneration. Dev Cell.

[14] Bencze M (2012). Proinflammatory macrophages enhance the regenerative capacity of human myoblasts by modifying their kinetics of proliferation and differentiation. Mol Ther.

[15] Cheng M (2008). Endogenous interferon-gamma is required for efficient skeletal muscle regeneration. Am J Physiol Cell Physiol.

[16] Perdiguero E (2011). p38/MKP-1-regulated AKT coordinates macrophage transitions and resolution of inflammation during tissue repair. J Cell Biol.

[17] Rigamonti E (2013). Requirement of inducible nitric oxide synthase for skeletal muscle regeneration after acute damage. J Immunol.

[18] Patsalos A (2019). The BACH1-HMOX1 regulatory axis is indispensable for proper macrophage subtype specification and skeletal muscle regeneration. J Immunol.

[19] De Micheli AJ (2020). Single-cell analysis of the muscle stem cell hierarchy identifies heterotypic communication signals involved in skeletal muscle regeneration. Cell Rep.

[20] Giordani L (2019). High-dimensional single-cell cartography reveals novel skeletal muscle-resident cell populations. Mol Cell.

[21] McKellar DW (2021). Large-scale integration of single-cell transcriptomic data captures transitional progenitor states in mouse skeletal muscle regeneration. Commun Biol.

[22] Dell’Orso S (2019). Single cell analysis of adult mouse skeletal muscle stem cells in homeostatic and regenerative conditions. Development.

[23] Hwang B (2018). Single-cell RNA sequencing technologies and bioinformatics pipelines. Exp Mol Med.

[24] Wagner A (2016). Revealing the vectors of cellular identity with single-cell genomics. Nat Biotechnol.

[25] Bergen V (2021). RNA velocity-current challenges and future perspectives. Mol Syst Biol.

[26] Oprescu SN (2020). Temporal dynamics and heterogeneity of cell populations during skeletal muscle regeneration. iScience.

[27] Scripture-Adams DD (2022). Single nuclei transcriptomics of muscle reveals intra-muscular cell dynamics linked to dystrophin loss and rescue. Commun Biol.

[28] Dries R (2021). Advances in spatial transcriptomic data analysis. Genome Res.

[29] Zhao E (2021). Spatial transcriptomics at subspot resolution with BayesSpace. Nat Biotechnol.

[30] Li H (2023). A comprehensive benchmarking with practical guidelines for cellular deconvolution of spatial transcriptomics. Nat Commun.

[31] Kleshchevnikov V (2022). Cell2location maps fine-grained cell types in spatial transcriptomics. Nat Biotechnol.

[32] Johnson JAI (2023). Inferring cellular and molecular processes in single-cell data with non-negative matrix factorization using Python, R and GenePattern Notebook implementations of CoGAPS. Nat Protoc.

[33] Mounier R (2013). AMPKα1 regulates macrophage skewing at the time of resolution of inflammation during skeletal muscle regeneration. Cell Metab.

[34] Wang H (2014). Altered macrophage phenotype transition impairs skeletal muscle regeneration. Am J Pathol.

[35] Panduro M (2018). T_reg_ cells limit IFN-γ production to control macrophage accrual and phenotype during skeletal muscle regeneration. Proc Natl Acad Sci U S A.

[36] Wang X (2020). Heterogeneous origins and functions of mouse skeletal muscle-resident macrophages. Proc Natl Acad Sci U S A.

[37] Varga T (2013). Tissue LyC6- macrophages are generated in the absence of circulating LyC6- monocytes and Nur77 in a model of muscle regeneration. J Immunol.

[38] Varga T (2016). Highly dynamic transcriptional signature of distinct macrophage subsets during sterile inflammation, resolution, and tissue repair. J Immunol.

[39] Korsunsky I (2019). Fast, sensitive, and accurate integration of single-cell data with Harmony. Nat Methods.

[40] Heng TS (2008). The Immunological Genome Project: networks of gene expression in immune cells. Nat Immunol.

[41] Patterson-Cross RB (2021). Selecting single cell clustering parameter values using subsampling-based robustness metrics. BMC Bioinformatics.

[42] Street K (2018). Slingshot: cell lineage and pseudotime inference for single-cell transcriptomics. BMC Genomics.

[43] Mass E (2023). Tissue-specific macrophages: how they develop and choreograph tissue biology. Nat Rev Immunol.

[44] Yu B (2018). Glycoprotein nonmelanoma clone B regulates the crosstalk between macrophages and mesenchymal stem cells toward wound repair. J Invest Dermatol.

[45] Robinet P (2021). Quantitative trait locus mapping identifies the Gpnmb gene as a modifier of mouse macrophage lysosome function. Sci Rep.

[46] Timmermans S (2017). Complete overview of protein-inactivating sequence variations in 36 sequenced mouse inbred strains. Proc Natl Acad Sci U S A.

[47] Anderson MG (2008). GpnmbR150X allele must be present in bone marrow derived cells to mediate DBA/2J glaucoma. BMC Genet.

[48] Ripoll VM (2007). Gpnmb is induced in macrophages by IFN-gamma and lipopolysaccharide and acts as a feedback regulator of proinflammatory responses. J Immunol.

[49] Kumagai K (2015). Glycoprotein nonmetastatic melanoma B (Gpnmb)-positive macrophages contribute to the balance between fibrosis and fibrolysis during the repair of acute liver injury in mice. PLoS One.

[50] Silva WN (2018). Macrophage-derived GPNMB accelerates skin healing. Exp Dermatol.

[51] Li B (2010). The melanoma-associated transmembrane glycoprotein Gpnmb controls trafficking of cellular debris for degradation and is essential for tissue repair. FASEB J.

[52] Quattrocelli M (2017). Genetic modifiers of muscular dystrophy act on sarcolemmal resealing and recovery from injury. PLoS Genet.

[53] Howell GR (2007). Absence of glaucoma in DBA/2J mice homozygous for wild-type versions of Gpnmb and Tyrp1. BMC Genet.

[54] Srivastava B (2009). Host genetic background strongly influences the response to influenza a virus infections. PLoS One.

[55] Hammers DW (2020). The D2.mdx mouse as a preclinical model of the skeletal muscle pathology associated with Duchenne muscular dystrophy. Sci Rep.

[56] Griggs RC (2016). Efficacy and safety of deflazacort vs prednisone and placebo for Duchenne muscular dystrophy. Neurology.

[57] Barp A (2015). Genetic modifiersof duchenne muscular dystrophy and dilated cardiomyopathy. PLoS One.

[58] Quattrocelli M (2017). Intermittent glucocorticoid steroid dosing enhances muscle repair without eliciting muscle atrophy. J Clin Invest.

[59] Hammers DW (2020). Glucocorticoids counteract hypertrophic effects of myostatin inhibition in dystrophic muscle. JCI Insight.

[60] Chen Y (2022). Insight into the molecular characteristics of langhans giant cell by combination of laser capture microdissection and RNA sequencing. J Inflamm Res.

[61] Stec MJ (2023). A cellular and molecular spatial atlas of dystrophic muscle. Proc Natl Acad Sci U S A.

[62] Coulis G (2023). Single-cell and spatial transcriptomics identify a macrophage population associated with skeletal muscle fibrosis. Sci Adv.

[63] Gutknecht M (2015). The transcription factor MITF is a critical regulator of GPNMB expression in dendritic cells. Cell Commun Signal.

[64] Gabriel TL (2014). Lysosomal stress in obese adipose tissue macrophages contributes to MITF-dependent Gpnmb induction. Diabetes.

[65] Ripoll VM (2008). Microphthalmia transcription factor regulates the expression of the novel osteoclast factor GPNMB. Gene.

[66] Labzin LI (2015). ATF3 is a key regulator of macrophage IFN Responses. J Immunol.

[67] Sha H (2017). ATF3 promotes migration and M1/M2 polarization of macrophages by activating tenascin‑C via Wnt/β‑catenin pathway. Mol Med Rep.

[68] Jadhav K, Zhang Y (2017). Activating transcription factor 3 in immune response and metabolic regulation. Liver Res.

[69] Liu S (2024). The dual roles of activating transcription factor 3 (ATF3) in inflammation, apoptosis, ferroptosis, and pathogen infection responses. Int J Mol Sci.

[70] De Nardo D (2014). High-density lipoprotein mediates anti-inflammatory reprogramming of macrophages via the transcriptional regulator ATF3. Nat Immunol.

[71] Kim M (2020). Single-nucleus transcriptomics reveals functional compartmentalization in syncytial skeletal muscle cells. Nat Commun.

[72] Krasniewski LK (2022). Single-cell analysis of skeletal muscle macrophages reveals age-associated functional subpopulations. Elife.

[73] Rubenstein AB (2020). Single-cell transcriptional profiles in human skeletal muscle. Sci Rep.

[74] Hume DA, MacDonald KP (2012). Therapeutic applications of macrophage colony-stimulating factor-1 (CSF-1) and antagonists of CSF-1 receptor (CSF-1R) signaling. Blood.

[75] Saade M (2021). The role of GPNMB in inflammation. Front Immunol.

[76] Zhou L (2017). Glycoprotein non-metastatic melanoma protein b (Gpnmb) is highly expressed in macrophages of acute injured kidney and promotes M2 macrophages polarization. Cell Immunol.

[77] Oyewumi MO (2016). Osteoactivin (GPNMB) ectodomain protein promotes growth and invasive behavior of human lung cancer cells. Oncotarget.

[78] Furochi H (2007). Osteoactivin fragments produced by ectodomain shedding induce MMP-3 expression via ERK pathway in mouse NIH-3T3 fibroblasts. FEBS Lett.

[79] Rose AA (2010). ADAM10 releases a soluble form of the GPNMB/Osteoactivin extracellular domain with angiogenic properties. PLoS One.

[80] Sondag GR (2016). Osteoactivin inhibition of osteoclastogenesis is mediated through CD44-ERK signaling. Exp Mol Med.

[81] Jin RM (2018). Chronic infection stunts macrophage heterogeneity and disrupts immune-mediated myogenesis. JCI Insight.

[82] Milias GA (2005). Effects of eccentric exercise-induced muscle injury on blood levels of platelet activating factor (PAF) and other inflammatory markers. Eur J Appl Physiol.

[83] Spadaro O (2022). Caloric restriction in humans reveals immunometabolic regulators of health span. Science.

[84] Yang T (2022). LILRB4, an immune checkpoint on myeloid cells. Blood Sci.

[85] Mastrogiovanni M (2022). Cell polarity regulators, multifunctional organizers of lymphocyte activation and function. Biomed J.

[86] Weiner OD (2006). Hem-1 complexes are essential for Rac activation, actin polymerization, and myosin regulation during neutrophil chemotaxis. PLoS Biol.

[87] Suwankitwat N (2021). The actin-regulatory protein Hem-1 is essential for alveolar macrophage development. J Exp Med.

[88] Ahmadzadeh K (2022). Multinucleated giant cells: current insights in phenotype, biological activities, and mechanism of formation. Front Cell Dev Biol.

[89] Mendell JR (2020). Assessment of systemic delivery of rAAVrh74.MHCK7.micro-dystrophin in children with duchenne muscular dystrophy: a nonrandomized controlled trial. JAMA Neurol.

[90] Emami MR (2023). Innate and adaptive AAV-mediated immune responses in a mouse model of Duchenne muscular dystrophy. Mol Ther Methods Clin Dev.

[91] Hart CC (2024). Potential limitations of microdystrophin gene therapy for Duchenne muscular dystrophy. JCI Insight.

[92] Guglieri M (2022). Efficacy and safety of vamorolone vs placebo and prednisone among boys with duchenne muscular dystrophy: a randomized clinical trial. JAMA Neurol.

[93] Finkel RS (2021). A randomized, double-blind, placebo-controlled, global phase 3 study of edasalonexent in pediatric patients with duchenne muscular dystrophy: results of the PolarisDMD Trial. J Neuromuscul Dis.

[94] Del Giudice E (2022). Off-label use of canakinumab in pediatric rheumatology and rare diseases. Front Med (Lausanne).

[95] Sciorati C (2016). Cell death, clearance and immunity in the skeletal muscle. Cell Death Differ.

[96] Aran D (2019). Reference-based analysis of lung single-cell sequencing reveals a transitional profibrotic macrophage. Nat Immunol.

[97] Qiu X (2017). Reversed graph embedding resolves complex single-cell trajectories. Nat Methods.

[98] Atta L (2022). VeloViz: RNA velocity-informed embeddings for visualizing cellular trajectories. Bioinformatics.

