# Supplementary material for: Spatiotemporal transcriptomic mapping of regenerative inflammation in skeletal muscle reveals a dynamic multilayered tissue architecture
Source: J Clin Invest. 2024 Aug 27;134(20):e173858. doi: 10.1172/JCI173858 (PMC11473166; doi:10.1172/JCI173858)
Supplement: Supplemental data [file jci-134-173858-s009.pdf]

Supplementary Figures

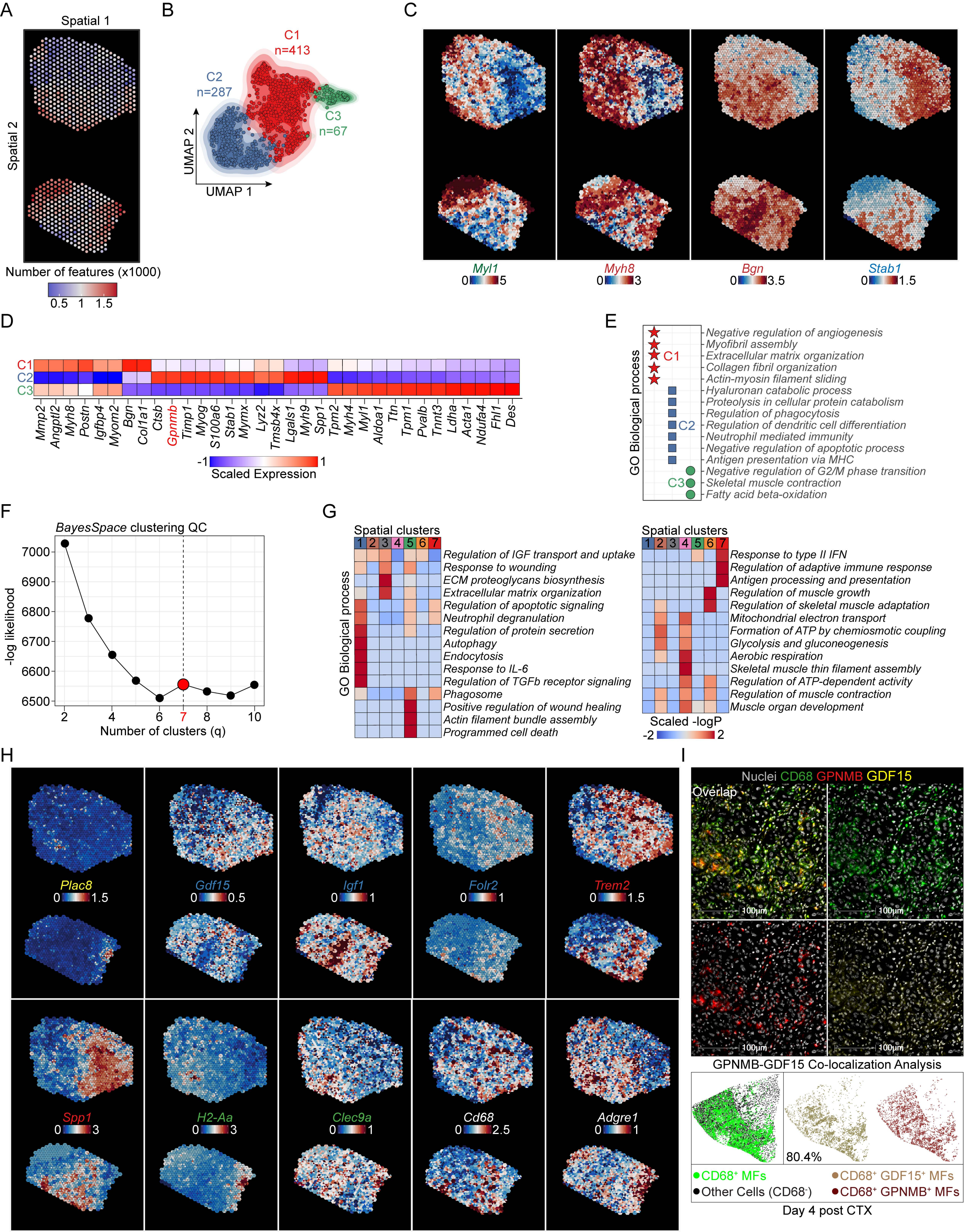
A
Spatial 1
Spatial 2
Number of features (x1000)
0.5 1 1.5
B
C1
n=413
C2
n=287
C3
n=67
UMAP 2
UMAP 1
C
Myl1
0 5
Myh8
0 3
Bgn
0 3.5
Stab1
0 1.5
D
C1
C2
C3
Mmp2 Angptl2 Myh8 Postn Igfbp4 Myom2 Bgn Col1a1 Ctsb Gpnmb Timp1 Myog S100a6 Stab1 Mymx Lyz2 Tmsb4x Lgals1 Myh9 Spp1 Tpm2 Myh4 Myl1 Aldoa1 Ttn Tpm1 Pvalb Tnnt3 Ldha Acta1 Ndufa4 Fhl1 Des
-1 1
Scaled Expression
E
GO Biological process
C1
C2
C3
Negative regulation of angiogenesis
Myofibril assembly
Extracellular matrix organization
Collagen fibril organization
Actin-myosin filament sliding
Hyaluronan catabolic process
Proteolysis in cellular protein catabolism
Regulation of phagocytosis
Regulation of dendritic cell differentiation
Neutrophil mediated immunity
Negative regulation of apoptotic process
Antigen presentation via MHC
Negative regulation of G2/M phase transition
Skeletal muscle contraction
Fatty acid beta-oxidation
F
BayesSpace clustering QC
-log likelihood
7000 6900 6800 6700 6600 6500
2 4 6 7 8 10
Number of clusters (q)
G
Spatial clusters
1 2 3 4 5 6 7
GO Biological process
Regulation of IGF transport and uptake
Response to wounding
ECM proteoglycans biosynthesis
Extracellular matrix organization
Regulation of apoptotic signaling
Neutrophil degranulation
Regulation of protein secretion
Autophagy
Endocytosis
Response to IL-6
Regulation of TGFb receptor signaling
Phagosome
Positive regulation of wound healing
Actin filament bundle assembly
Programmed cell death
Spatial clusters
1 2 3 4 5 6 7
Response to type II IFN
Regulation of adaptive immune response
Antigen processing and presentation
Regulation of muscle growth
Regulation of skeletal muscle adaptation
Mitochondrial electron transport
Formation of ATP by chemiosmotic coupling
Glycolysis and gluconeogenesis
Aerobic respiration
Skeletal muscle thin filament assembly
Regulation of ATP-dependent activity
Regulation of muscle contraction
Muscle organ development
Scaled -logP
-2 2
H
Plac8
0 1.5
Gdf15
0 0.5
Igf1
0 1
Folr2
0 1
Trem2
0 1.5
Spp1
0 3
H2-Aa
0 3
Clec9a
0 1
Cd68
0 2.5
Adgre1
0 1
I
Nuclei CD68 GPNMB GDF15
Overlap
100µm
GPNMB-GDF15 Co-localization Analysis
80.4%
CD68⁺ MFs
Other Cells (CD68⁻)
CD68⁺ GDF15⁺ MFs
CD68⁺ GPNMB⁺ MFs
Day 4 post CTX

Supplementary Figure S1 (related to main Figure 1).

A. Number of genes at spot-level resolution of TA muscles at day 4 post-CTX.

B. UMAP representation of all spots from the ST samples colored by the Leiden-guided clustering as in **Fig. 1A**. The number of spots per cluster is indicated.

C. Spatial expression patterns at *BayesSpace* subspot resolution of canonical healthy (*Myl1*), regenerative (*Myh8, Bgn*), and inflammatory (*Stab1*) gene expression by Visium spot. The color scale shows the log-normalized counts for each subspot.

D. Differential expression analysis between the three Leiden/pathologist-predicted clusters highlighted spatial differences in the expression of immune genes, regeneration markers, and genes encoding extracellular matrix proteins. GPNMB, a recently identified GFEM marker, is highlighted (1). The average marker gene expression after z-score transformation is shown for each cluster.

E. Gene ontology pathway analysis of the three Leiden-predicted and pathologist-annotated clusters. Top enriched pathways with $p<0.001$ and fold enrichment > 2 are shown for each cluster.

F. Negative-log likelihood plot. The elbow at $q=7$ was selected as the number of clusters to analyze.

G. Gene ontology pathway analysis of the seven *BayesSpace*-predicted clusters in panel **1C**. Top enriched pathways with fold enrichment > 5 are shown, indicating their correlation to the other clusters (scaled by negative log of P value).

H. Spatial expression patterns at *BayesSpace* subspot resolution of marker genes defining the myeloid subsets previously characterized by scRNA-seq (1). The color scale shows the log-normalized counts for each subspot. The color of the gene label corresponds to the MF and DC subset classification shown in panel **1H**.

I. Upper panel: IF images of CD68 (green), GPNMB (red), GDF15 (yellow), and nuclei (grey) stained muscles in C57BL/6J animals at day 4 post-CTX. Insets indicate split channels. Scale bars: 100 µm. Lower panel: Co-localization and distribution map of total $CD68^+$ MFs (green), $CD68^+GPNMB^+$ subset (gold), $CD68^+GDF15^+$ subset (red), and other $CD68^-$ cells (black) quantified by HALO's *Cytonuclear* and *Spatial Proximity*/co-localization modules. The percent of co-localization (80.4%) between $CD68^+GPNMB^+$ and $CD68^+GDF15^+$ subsets is indicated.

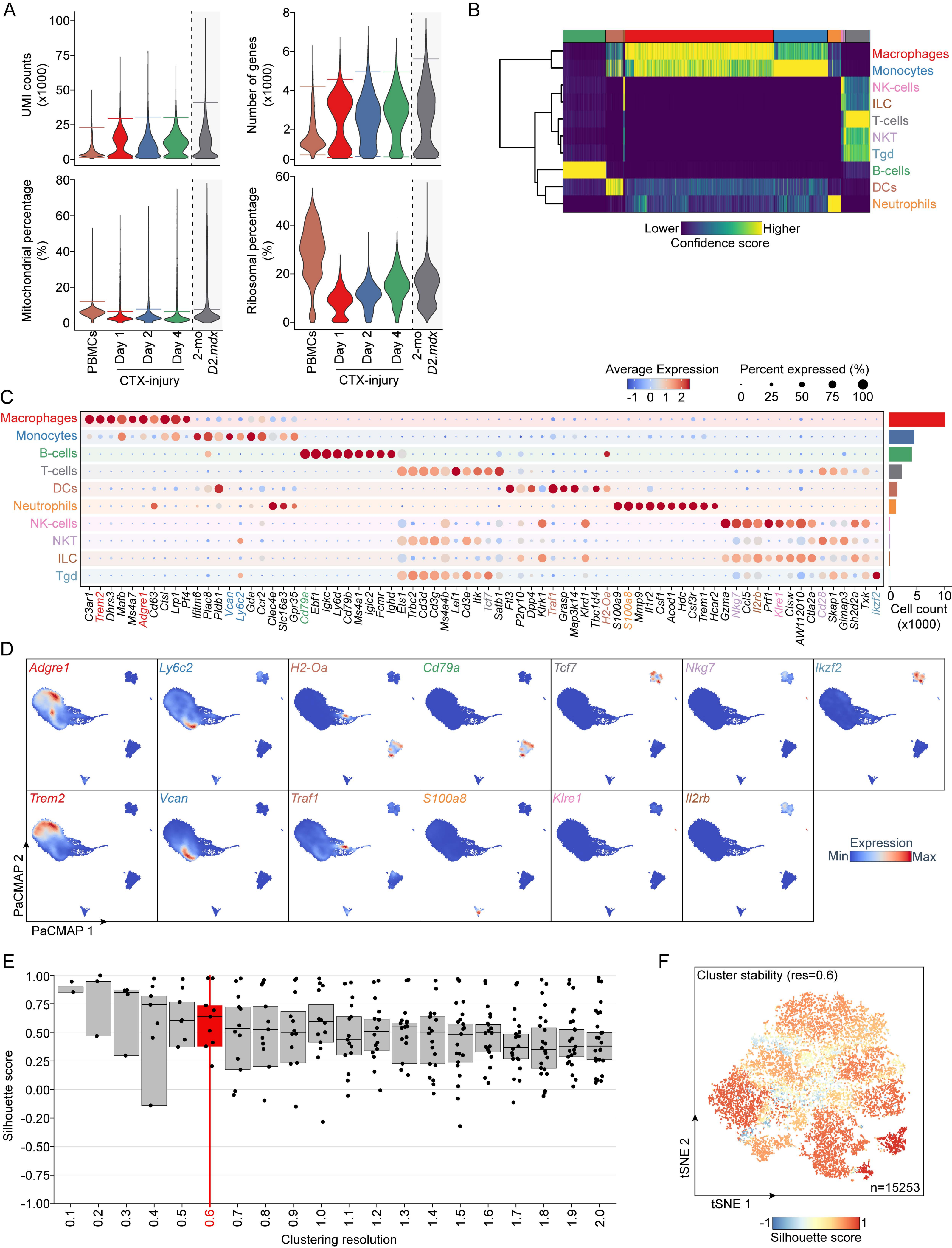
A
UMI counts (x1000)
Number of genes (x1000)
Mitochondrial percentage (%)
Ribosomal percentage (%)
PBMCs
Day 1
Day 2
Day 4
2-mo D2.mdx
CTX-injury
B
Macrophages
Monocytes
NK-cells
ILC
T-cells
NKT
Tgd
B-cells
DCs
Neutrophils
Lower
Higher
Confidence score
C
Average Expression
Percent expressed (%)
0 25 50 75 100
Cell count (x1000)
C3ar1 Trem2 Dhrs3 Mafb Ms4a7 Adgre1 Cd63 Ctsl Lrp1 Pf4 Ifitm6 Plac8 Pldb1 Vcan Ly6c2 Gda Ccr2 Clec4e Slc16a3 Gpr35 Cd79a Ebf1 Igkc Ly6d Cd79b Ms4a1 Iglc2 Fcmr Ighd Ets1 Trbc2 Cd3d Cd3g Ms4a4b Lef1 Cd3e Itk Tcf7 Satb1 Flt3 P2ry10 Dpp4 Klrk1 Traf1 Grasp Map3k14 Klrd1 Tbc1d4 H2-Oa S100a9 S100a8 Mmp9 Il1r2 Csf1 Acod1 Hdc Csf3r Trem1 Hcar2 Gzma Nkg7 Ccl5 Il2rb Prf1 Klre1 Ctsw AW112010 Ctla2a Cd28 Skap1 Gimap3 Sh2d2a Txk Ikzf2
D
Adgre1
Ly6c2
H2-Oa
Cd79a
Tcf7
Nkg7
Ikzf2
Trem2
Vcan
Traf1
S100a8
Klre1
Il2rb
PaCMAP 2
PaCMAP 1
Expression
Min
Max
E
Silhouette score
Clustering resolution
F
Cluster stability (res=0.6)
tSNE 2
tSNE 1
n=15253
Silhouette score

Supplementary Figure S2 (related to main Figure 2).

A. Violin plots representing the number of unique molecular identifiers (UMIs; upper left panel), genes per cell (upper right panel), mitochondrial (lower left panel) and ribosomal genes percentage (lower right panel) of all cells from PBMC, day 1, 2 and 4 post-CTX and 2-mo D2.*mdx* $CD45^+$ cells before QC filtering and clustering. Colored lines indicate the filtering parameter cutoff values. The cellular source is color-coded.

B. Cell type annotation confidence score matrix visualized as a heatmap. Each cell is a column, while each row is a label in the integrated scRNA-seq dataset (PBMCs, day 1, 2, and 4 post-CTX $CD45^+$ cells). The final label (after fine-tuning) for each cell is shown in the top color bar.

C. Dot-plot showing top DE genes that distinguish the 10 major cell type clusters (integrated dataset from PBMCs, day 1, 2, and 4 post-CTX $CD45^+$ cells). The dot size represents the percentage of cells expressing the respective marker gene within a cluster. Selected markers are color coded.

D. PaCMAP feature plots (expression z-score) of selected markers from panel **S2C** that define the various cell types from the regeneration time course. Selected markers are color coded as in panel **S2C**.

E. Silhouette distribution plot for the isolated monocyte/MF/DC scRNA-seq dataset in **Fig. 2D**. Each dot represents a cluster at a given clustering parameter value. Medians with 95% CI are shown for each parameter value. The vertical red line marks the optimal resolution.

F. t-SNE representation of cells, colored by silhouette score at the suggested optimal resolution value = 0.6.

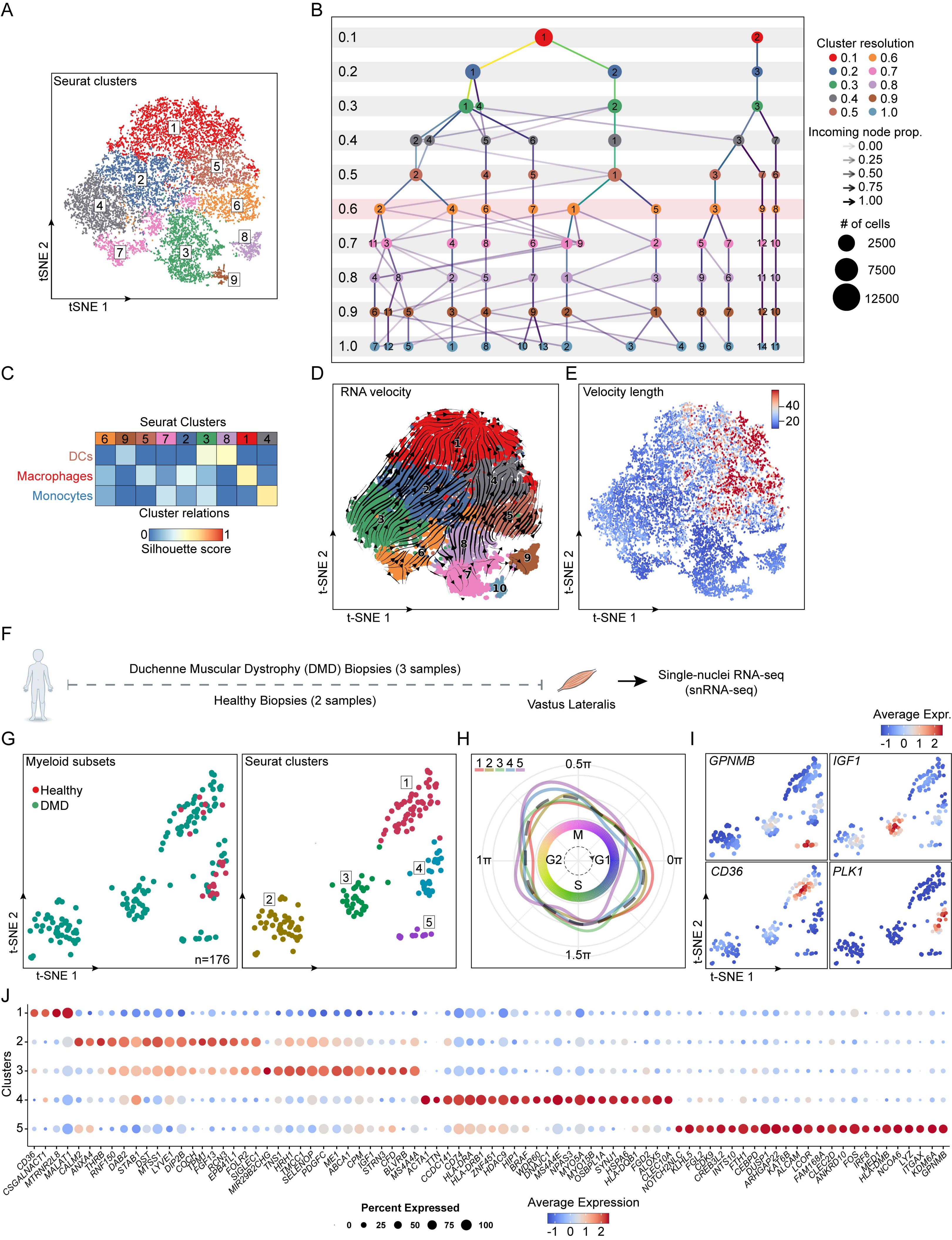
A
Seurat clusters
tSNE 1
tSNE 2
B
Cluster resolution
0.1
0.2
0.3
0.4
0.5
0.6
0.7
0.8
0.9
1.0
Incoming node prop.
0.00
0.25
0.50
0.75
1.00
# of cells
2500
7500
12500
C
Seurat Clusters
DCs
Macrophages
Monocytes
Cluster relations
Silhouette score
D
RNA velocity
t-SNE 1
t-SNE 2
E
Velocity length
F
Duchenne Muscular Dystrophy (DMD) Biopsies (3 samples)
Healthy Biopsies (2 samples)
Vastus Lateralis
Single-nuclei RNA-seq
(snRNA-seq)
G
Myeloid subsets
Healthy
DMD
n=176
Seurat clusters
H
0.5π
1π
0π
1.5π
M
G2
G1
S
I
Average Expr.
GPNMB
IGF1
CD36
PLK1
J
Clusters
CD36
CSGALNACT1
MTRNR2L8
MALAT1
CALM2
ANXA4
THRB
RNF150
DAB2
STAB1
DST
MTSS1
LYVE1
DIP2B
COCH
TPM1
FGF13
RCN3
EPB41L1
FOLR2
SIGLEC1
MIR29B2CHG
TNS1
HRH1
TMCC1
SELENOP
PDGFC
ME1
ABCA1
CPM
IGF1
STRN3
CFD
BLVRB
MS4A4A
ACTA1
TTN
CCDC141
CD74
HLA-DRA
HLA-DRB1
ZNF451
HDAC9
HIP1
BRAF
WDR60
DNAJC1
MS4A4E
NPAS3
MYO5A
MYO15B
OSBPL1A
SYNJ1
HSPA6
HLA-DQB1
FGD2
ALOX5
CLEC10A
NOTCH2NLC
KLHL5
FGL2
CDK9
CREB3L2
INTS10
FTH1
CEBPD
DUSP1
ARHGAP22
KAT6B
ALCAM
LCOR
FAM168A
CLEC2D
ANKRD10
FOS
IRF8
MED1
HLA-DMB
NCOA6
LYZ
ITGAX
KDM6A
GPNMB
Percent Expressed
0
25
50
75
100
Average Expression

Supplementary Figure S3 (related to main Figures 2 and 4).

A. t-SNE representation of Seurat clusters (integrated dataset from PBMCs, day 1, 2, and 4 post-CTX $CD45^+$ cells) at resolution 0.6 results in nine clusters.

B. Clustering tree (2, 3) visualizing the relationships between resolution parameters from 0.1 to 1 (step 0.1). At a resolution of 0.6, we see the formation of 9 main branches, one of which (cluster 3) continues to split up to a resolution of 0.7, after which there are only minor changes.

C. Heatmap of silhouette score at the predicted optimal resolution value = 0.6, indicating cluster and myeloid cell type relations. Cluster 3 shows a large overlap with all three myeloid subtypes (also shown in **Fig. 2D** with cell type annotations), supporting our decision to split this cluster.

D. Arrows indicating the projection of the velocities derived from the *scVelo* dynamical model (4) of the monocyte/MF/DC subtypes are projected into a t-SNE-based embedding.

E. t-SNE visualization of the speed/rate of differentiation given by the length of the velocity vector (*scVelo* dynamic modeling) (4) in the isolated monocyte/MF/DC scRNA-seq dataset.

F. Study schematic and workflow for the analysis of single-nuclei RNA-sequencing of human (healthy and DMD patients) vastus lateralis biopsies (5).

G. t-SNE visualization of myeloid subsets extracted from the human muscle single-nuclei RNA-sequencing datasets. Left panel: Indicates the origin of the myeloid subsets (red indicates cells from the healthy muscle biopsies and green cells originating from the DMD patients). Right panel: SNN clustering resolved five distinct myeloid subsets that are color-coded.

H. 2D embeddings visualizing cell cycle phases of the five myeloid subsets in the human snRNA-seq datasets generated using *VeloViz* embeddings. Cycling MFs are a conserved feature of dystrophy-mediated chronic inflammation in human DMD pathology.

I. Single-nuclei expression levels for selected functional markers. These markers allowed the delineation of functionally distinct MF subtypes present in human DMD biopsies. PLK1 indicates the presence of cycling MFs.

J. Top marker genes for the five identified MF clusters in the human snRNA-seq datasets. The dot size represents the percentage of cells within a group with an expression level > 0 and color-scale represents the average expression level (row Z-score) across all cells within the cluster.

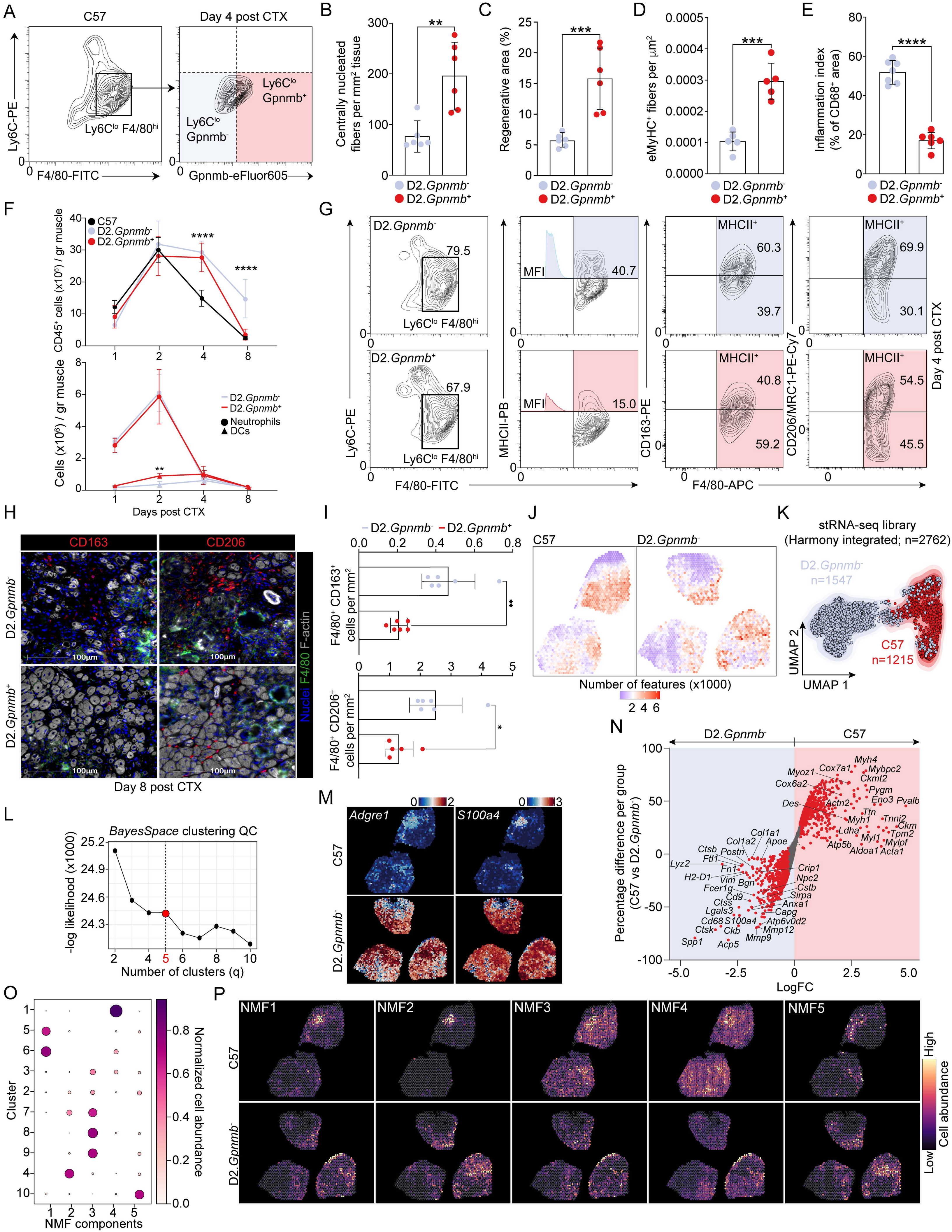

A
C57
Day 4 post CTX
Ly6C-PE
F4/80-FITC
Gpnmb-eFluor605
Ly6Clo F4/80hi
Ly6Clo Gpnmb+
Ly6Clo Gpnmb-
B
Centrally nucleated fibers per mm2 tissue
C
Regenerative area (%)
D
eMyHC+ fibers per µm2
E
Inflammation index (% of CD68+ area)
D2.Gpnmb-
D2.Gpnmb+
F
C57
CD45+ cells (x106) / gr muscle
Cells (x106) / gr muscle
Neutrophils
DCs
Days post CTX
G
MHCII-PB
CD163-PE
CD206/MRC1-PE-Cy7
F4/80-APC
MHCII+
MFI
Day 4 post CTX
H
CD163
CD206
Nuclei F4/80 F-actin
100µm
Day 8 post CTX
I
F4/80+ CD163+ cells per mm2
F4/80+ CD206+ cells per mm2
J
Number of features (x1000)
K
stRNA-seq library (Harmony integrated; n=2762)
D2.Gpnmb- n=1547
C57 n=1215
UMAP 1
UMAP 2
L
BayesSpace clustering QC
-log likelihood (x1000)
Number of clusters (q)
M
Adgre1
S100a4
N
Percentage difference per group (C57 vs D2.Gpnmb-)
LogFC
O
Cluster
NMF components
Normalized cell abundance
P
NMF1
NMF2
NMF3
NMF4
NMF5
Cell abundance
High
Low

Supplementary Figure S4 (related to main Figure 3).

A. FACS contour plots and gating strategy of $CD45^+$ $Ly6C^{low}$ $F4/80^{hi}$ $GPNMB^+$ and $CD45^+$ $Ly6C^{low}$ $F4/80^{hi}$ $GPNMB^-$ muscle-infiltrating MFs in WT C57BL/6J animals at day 4 post-CTX.

B. Number of centrally nucleated myofibers per mm^2 of tissue in D2.*Gpnmb*$^+$ and D2.*Gpnmb*$^-$ (DBA2/J) muscles at day 8 post-CTX injury (n=6 per group). Unpaired t-test was performed with P value 0.0022.

C. Percentage of regeneration area over the total injured area in D2.*Gpnmb*$^+$ and D2.*Gpnmb*$^-$ (DBA2/J) muscles at day 8 post-CTX injury (n=6 per group). Unpaired t-test was performed with P value 0.0007.

D. Number of regenerating myofibers (assessed by embryonic MyHC positivity) per mm^2 of tissue in D2.*Gpnmb*$^+$ and D2.*Gpnmb*$^-$ (DBA2/J) muscles at day 8 post-CTX injury (n=5 per group). Unpaired t-test was performed with P value 0.0002.

E. Percentage of inflammation area (assessed by CD68 positivity) over the total injured area in D2.*Gpnmb*$^+$ and D2.*Gpnmb*$^-$ (DBA2/J) muscles at day 8 post-CTX injury (n=6 per group). Unpaired t-test with Welch's correction was performed with P value < 0.0001.

F. Upper panel: Number of $CD45^+$ cells per g of tissue in C57BL/6J, D2.*Gpnmb*$^+$ and D2.*Gpnmb*$^-$ (DBA2/J) muscles at indicated time points post-CTX injury (n=6 per group). Two-way ANOVA with Dunnett's multiple comparisons test was performed between genotypes and time points (Day 4: C57BL/6J vs D2.*Gpnmb*$^+$ and C57BL/6J vs D2.*Gpnmb*$^-$ and Day 8: C57BL/6J vs D2.*Gpnmb*$^-$ and D2.*Gpnmb*$^+$ vs D2.*Gpnmb*$^-$ comparisons have P < 0.0001). Lower panel: Number of DCs ($CD45^+CD11c^+$ $F4/80^-$ $Ly6C^-$) and neutrophils ($CD45^+$ $Ly6G^+F4/80^-Ly6C^{int}$) per g of tissue in D2.*Gpnmb*$^+$ and D2.*Gpnmb*$^-$ (DBA2/J) muscles at indicated time points post-CTX injury (n=6 per group). Two-way ANOVA with Dunnett's

multiple comparisons test was performed between genotypes and time points (Day 2 DCs: D2.*Gpnmb*$^+$ vs D2.*Gpnmb*$^-$ comparison has $P < 0.01$).

G. FACS contour plots of CD45$^+$ Ly6C^{low} F4/80^{hi} MHCII$^+$ and CD45$^+$ Ly6C^{low} F4/80^{hi} MHCII$^-$ muscle-infiltrating MFs in D2.*Gpnmb*$^+$ and D2.*Gpnmb*$^-$ animals at day 4 post-CTX. Insets indicate the frequency and MHCII MFI for each population (n=6 biological replicates per group). The frequency of CD163 and CD206 in CD45$^+$ Ly6C^{low} F4/80^{hi} MHCII$^+$ is also shown.

H. IF detection of CD163 (left panel; red) and CD206 (right panel; red) MFs (F4/80; green) in D2.*Gpnmb*$^+$ and D2.*Gpnmb*$^-$ animals at day 8 post-CTX injury (F-actin/phalloidin is indicated in gray and nuclei in blue). Scale bars: 100 µm.

I. Quantification of CD163$^+$ F4/80$^+$ (upper panel) and CD163$^+$ F4/80$^+$ (lower panel) in D2.*Gpnmb*$^+$ and D2.*Gpnmb*$^-$ animals at day 8 post-CTX injury. Unpaired t-test with Welch's correction was performed in each comparison with P values 0.0048 and 0.0185, respectively.

J. Number of genes at spot-level resolution in the ST day 8 post-CTX injured muscle samples from C57BL/6J and D2.*Gpnmb*$^-$ animals.

K. UMAP visualization of all spots in the *harmony*-integrated ST datasets from C57BL/6J and D2.*Gpnmb*$^-$ at day 8 post-CTX. The number of spots per condition is indicated.

L. Negative-log likelihood plot. The elbow at q=5 was selected as the number of clusters for downstream analysis.

M. Spatial expression of representative mature MFs marker genes (*Adgre1* and *S100a4*) in WT (C57BL/6J; upper) and D2.*Gpnmb*$^-$ (GPNMB KO; lower) samples from Day 8 post CTX. Note the increased and prolonged presence of MFs in the absence of GPNMB.

N. Volcano plot indicating the DE genes between C57BL/6J vs D2.*Gpnmb*$^-$ spatial spots (padj<0.05, logFC>0.25). The enriched gene set for each sample group is color-coded and the top gene labels are shown.

O. Dot plot of the estimated/relative NMF weights and cell abundance of ten subtypes (rows) described in **Fig. 2** across five predicted NMF components (columns) in the day 8 post-CTX spatial samples.

P. Identification of tissue compartments in the day 8 post-CTX samples (upper: C57BL/6J, lower: D2.*Gpnmb*$^-$) using NMF-based decomposition (*Cell2location*) and reference immune subtype expression signatures from **Fig. 2**. Spatial plots show cell abundance (color intensity) for each subtype.

In all bar graphs, bars represent mean ± SD (**p < 0.01, ***p < 0.001, ****p < 0.0001).

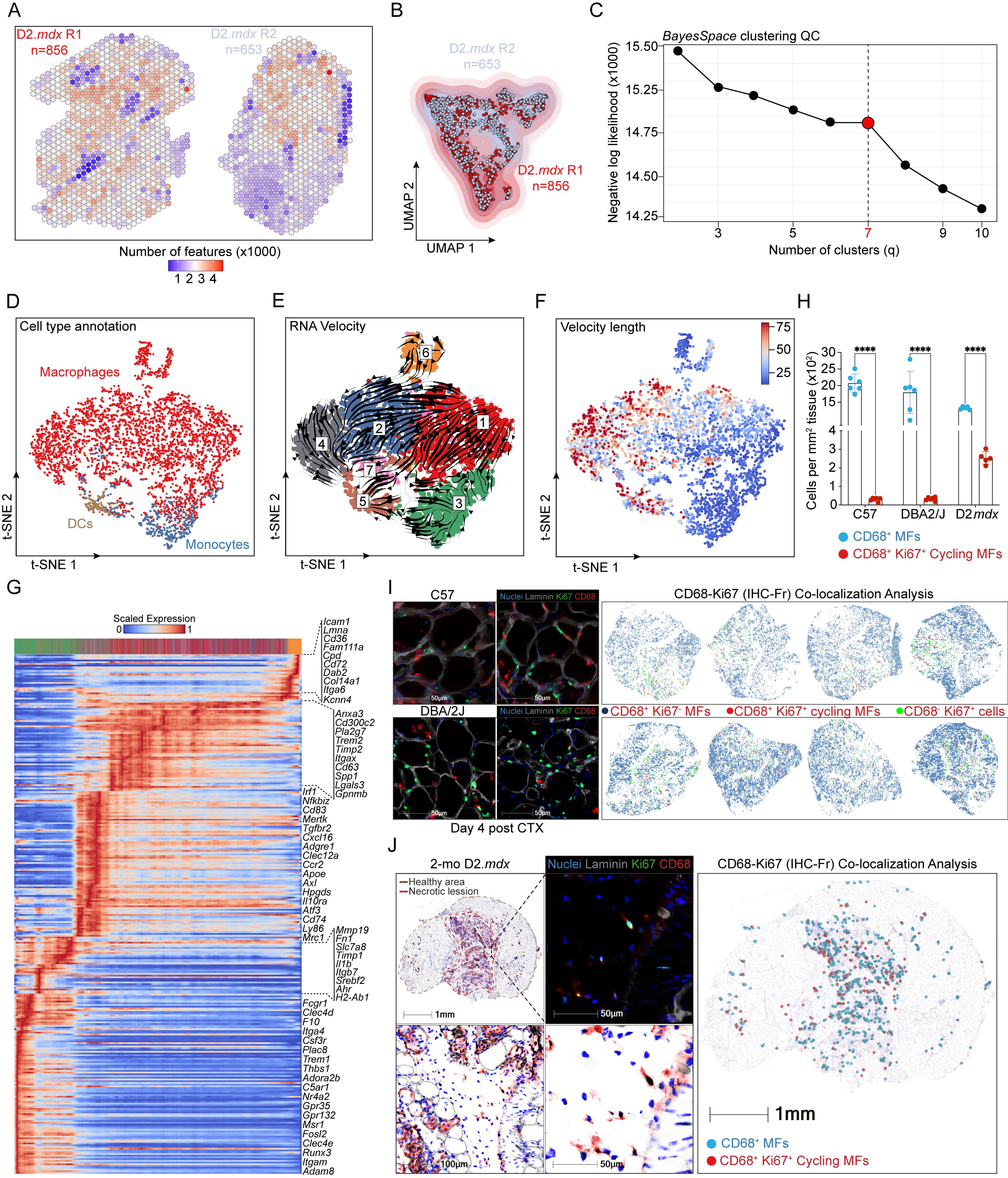
A
D2.mdx R1
n=856
D2.mdx R2
n=653
Number of features (x1000)
1 2 3 4
B
D2.mdx R2
n=653
D2.mdx R1
n=856
UMAP 2
UMAP 1
C
BayesSpace clustering QC
Negative log likelihood (x1000)
15.50
15.25
14.75
14.50
14.25
3
5
7
9
10
Number of clusters (q)
D
Cell type annotation
Macrophages
DCs
Monocytes
t-SNE 2
t-SNE 1
E
RNA Velocity
1
2
3
4
5
6
7
t-SNE 2
t-SNE 1
F
Velocity length
75
50
25
t-SNE 2
t-SNE 1
H
Cells per mm² tissue (x10²)
30
25
20
15
4
3
2
1
0
****
****
****
C57
DBA2/J
D2mdx
CD68⁺ MFs
CD68⁺ Ki67⁺ Cycling MFs
G
Scaled Expression
0
1
Icam1
Lmna
Cd36
Fam111a
Cpd
Cd72
Dab2
Col14a1
Itga6
Kcnn4
Anxa3
Cd300c2
Pla2g7
Trem2
Timp2
Itgax
Cd63
Spp1
Lgals3
Gpnmb
Irf1
Nfkbiz
Cd83
Mertk
Tgfbr2
Cxcl16
Adgre1
Clec12a
Ccr2
Apoe
Axl
Hpgds
Il10ra
Atf3
Cd74
Ly86
Mrc1
Mmp19
Fn1
Slc7a8
Timp1
Il1b
Itgb7
Srebf2
Ahr
H2-Ab1
Fcgr1
Clec4d
F10
Itga4
Csf3r
Plac8
Trem1
Thbs1
Adora2b
C5ar1
Nr4a2
Gpr35
Gpr132
Msr1
Fosl2
Clec4e
Runx3
Itgam
Adam8
I
C57
Nuclei Laminin Ki67 CD68
50μm
DBA/2J
Day 4 post CTX
CD68-Ki67 (IHC-Fr) Co-localization Analysis
CD68⁺ Ki67⁻ MFs
CD68⁺ Ki67⁺ cycling MFs
CD68⁻ Ki67⁺ cells
J
2-mo D2.mdx
Healthy area
Necrotic lession
1mm
Nuclei Laminin Ki67 CD68
50μm
100μm
CD68-Ki67 (IHC-Fr) Co-localization Analysis
1mm
CD68⁺ MFs
CD68⁺ Ki67⁺ Cycling MFs

Supplementary Figure S5 (related to main Figure 4).

A. Number of genes at spot-level resolution of two gastrocnemius muscles from 2-mo D2.*mdx* animals.

B. UMAP representation of the integrated spots from 2 biological replicates. The number of spots per biological replicate is indicated.

C. Negative-log likelihood plot. The elbow at q=7 was selected as the number of clusters to analyze.

D. t-SNE visualization of the monocyte/MF/DC cells from the D2.*mdx* scRNA-seq sample. *SingleR (ImmGen* database*)* cell type annotation prediction was used for the classification and cell type selection.

E. Arrows indicating the projection of the velocities derived from the *scVelo* dynamical model (4) of the monocyte/MF/DC subtypes in the D2.*mdx* scRNA-seq dataset are projected into a t-SNE-based embedding.

F. t-SNE visualization of the speed/rate of differentiation given by the length of the velocity vector (*scVelo* dynamic modeling) (4) in the 2-mo D2.*mdx* monocyte/MF/DC scRNA-seq dataset.

G. Gene expression dynamics of the 7 monocyte/MF/DC subpopulations in the scRNA-seq D2.*mdx* dataset resolved along latent time (*scVelo* dynamic modeling). The top likelihood-ranked genes predicted to drive cell differentiation are ordered within each branch.

H. Number of non-proliferating $CD68^{+}$ $Ki67^{-}$ and $CD68^{+}$ $Ki67^{+}$ cycling MFs per mm^2 of tissue in C57BL/6J and DBA2/J animals at day 4 post-CTX injury and in 2-mo D2.*mdx* animals (an unpaired t-test with Welch's correction was performed for all indicated comparisons with P values < 0.0001).

I. Left panel: IF images (inverted and color-coded) of CD68 (red), Laminin (orange), Ki67 (green), and nuclei (blue) stained muscles from C57BL/6J and DBA2/J animals at day 4 post-CTX injury for the detection of cycling MFs. No cycling double-positive MFs were detected. Scale bars: 50 µm. Right panel: Co-localization and distribution map of $CD68^{+}$ $Ki67^{-}$ non-proliferating MFs (blue), $CD68^{+}Ki67^{+}$ cycling MFs (red), and non-MF proliferating cells ($CD68^{-}Ki67^{+}$) quantified by HALO co-localization modules.

J. Left panel: IF images (inverted and color-coded) of CD68 (red), Laminin (grey), Ki67 (green), and nuclei (blue) stained muscles from 2-mo D2.*mdx* animals for the detection of cycling MFs. Scale bars: macroscopic views 1 mm; insets 50 and 100 µm. Right panel: Co-localization and distribution map of $CD68^{+}$ MFs (blue) and $CD68^{+}Ki67^{+}$ cycling MFs (red) quantified by HALO co-localization modules.

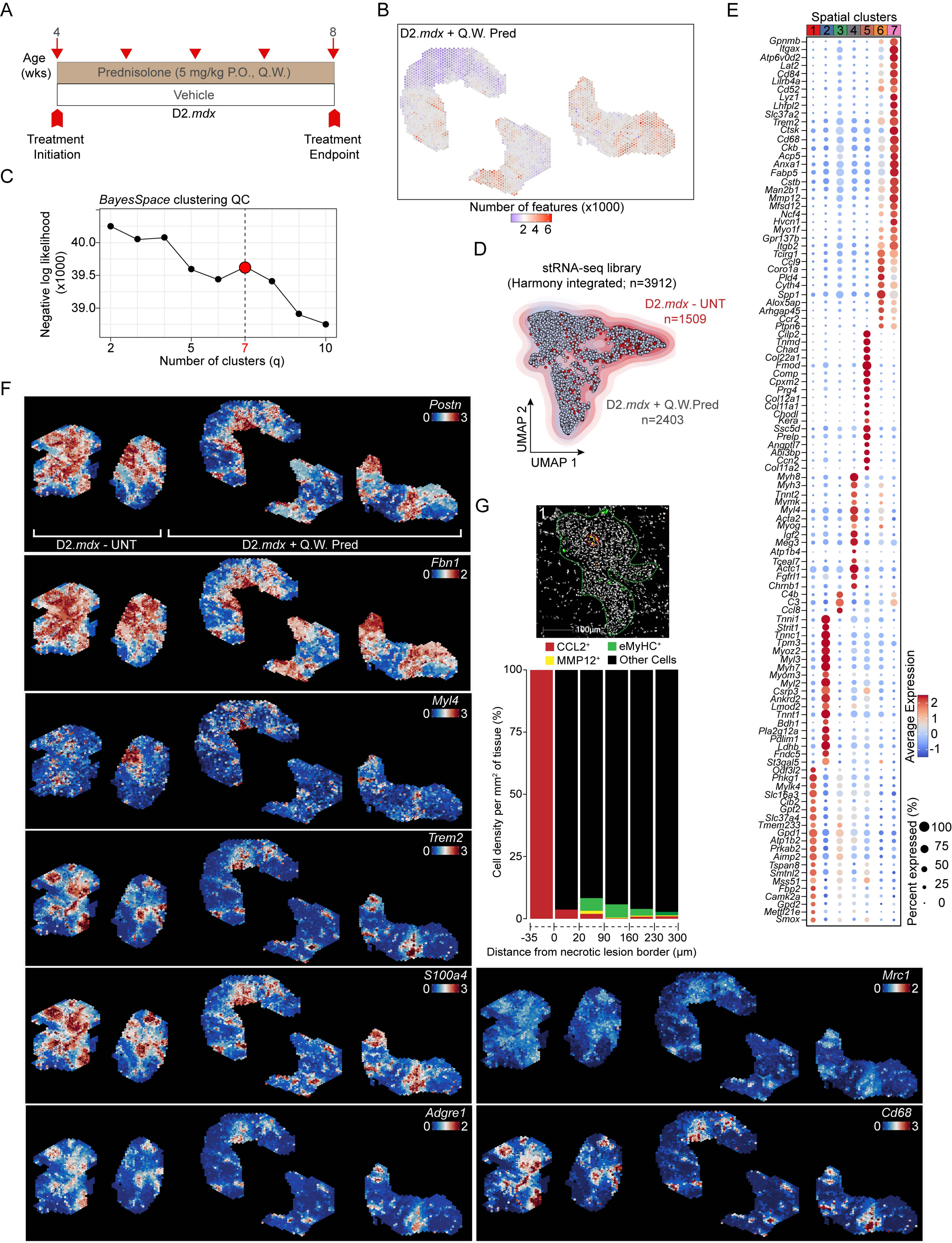
A
Age (wks)
4
8
Prednisolone (5 mg/kg P.O., Q.W.)
Vehicle
D2.mdx
Treatment Initiation
Treatment Endpoint
B
D2.mdx + Q.W. Pred
Number of features (x1000)
2 4 6
C
BayesSpace clustering QC
Negative log likelihood (x1000)
Number of clusters (q)
D
stRNA-seq library
(Harmony integrated; n=3912)
D2.mdx - UNT n=1509
D2.mdx + Q.W.Pred n=2403
UMAP 1
UMAP 2
E
Spatial clusters
Average Expression
Percent expressed (%)
F
Postn
Fbn1
Myl4
Trem2
S100a4
Mrc1
Adgre1
Cd68
D2.mdx - UNT
D2.mdx + Q.W. Pred
G
100µm
CCL2+
eMyHC+
MMP12+
Other Cells
Cell density per mm² of tissue (%)
Distance from necrotic lesion border (µm)

Supplementary Figure S6 (related to main Figures 6 and 7).

A. Experimental schematic workflow. 4-week-old D2.*mdx* animals were treated weekly orally with 5 mg/kg Prednisolone for 4 weeks. Gastrocnemius muscles were then subjected to ST.

B. Number of genes at spot-level resolution of three gastrocnemius muscles from 2-mo D2.*mdx* + Q.W. Pred animals.

C. Negative-log likelihood plot. The elbow at *q*=7 was selected as the number of clusters to analyze.

D. UMAP representation of the integrated spots from D2.*mdx*-UNT and D2.*mdx* + Q.W. Pred. The number of spots per condition is indicated.

E. Top marker gene expression after z-score transformation is shown for each spatial cluster. The dot size represents the percentage of cells within a group.

F. Spatial feature plots of representative DE genes from the D2.*mdx* - UNT *vs.* D2.*mdx* + Q.W. Pred. comparison is shown.

G. Upper panel: A magnified IF image of an inflammatory lesion in 2-mo D2.mdx + Q.W. Pred GAST muscle (the lesion location label is indicated in the upper left corner; see also **Figs. 7E** and **S7C** for different magnifications and more representative examples). The MF subtypes were visualized with CCL2 (red; Zone A) and MMP12 (yellow; Zone B) and regenerating fibers with eMyHC (green; Zone C). Scale bar: 100 µm. Lower panel: A stacked bar histogram reflecting CCL2^{+}, MMP12^{+}, eMyHC^{+}, and Other Cells (CCL2^{-} MMP12^{-} eMyHC^{-}) cell density inside (-1 to -50 µm) and outside the necrotic boundary (+1 to +300 µm). Note the near complete absence of MF subtypes (i.e., MMP12^{+} cells), regenerating fibers (eMyHC^{+} cells), and unstructured damage-clearing and RIZ tissue organization.

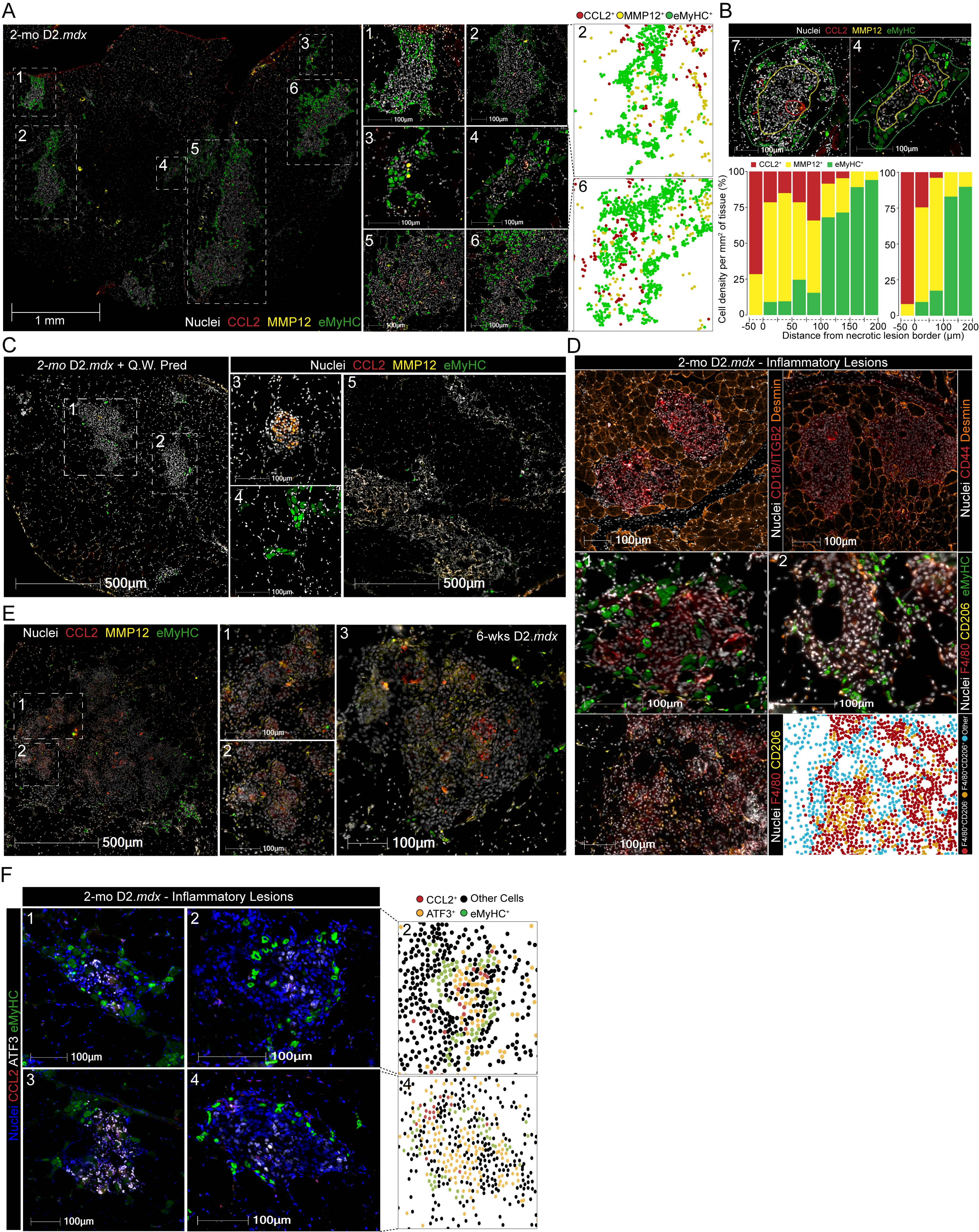
A
2-mo D2.mdx
1 mm
Nuclei CCL2 MMP12 eMyHC
100µm
CCL2+ MMP12+ eMyHC+
B
Nuclei CCL2 MMP12 eMyHC
100µm
CCL2+ MMP12+ eMyHC+
Cell density per mm2 of tissue (%)
Distance from necrotic lesion border (µm)
C
2-mo D2.mdx + Q.W. Pred
Nuclei CCL2 MMP12 eMyHC
500µm
100µm
D
2-mo D2.mdx - Inflammatory Lesions
Nuclei CD18/ITGB2 Desmin
Nuclei CD44 Desmin
Nuclei F4/80 CD206 eMyHC
Nuclei F4/80 CD206
F4/80+CD206- F4/80+CD206+ Other
100µm
E
Nuclei CCL2 MMP12 eMyHC
6-wks D2.mdx
500µm
100µm
F
2-mo D2.mdx - Inflammatory Lesions
Nuclei CCL2 ATF3 eMyHC
CCL2+ Other Cells
ATF3+ eMyHC+
100µm

Supplementary Figure S7 (related to main Figures 7 and 8).

A. Six representative regions of RIZs in 2-mo D2.*mdx* GAST validated by IF. The MF subtypes and zones were visualized with IF for CCL2 (red; Zone A), MMP12 (yellow; Zone B), and eMyHC (green; Zone C). White rectangles indicate the region magnified in the middle panel. Scale bars: macroscopic view 1 mm; insets 100 µm. Right panel indicates the cell density and distribution of cells in regions 2 and 6. Note the consistent and expected organization and architecture of RIZs.

B. RIZ quantification in 2-mo D2.*mdx* muscles. Dotted lines indicate the zones and interface layer (red; necrotic lesion) selected for cell density quantification. The bottom panels show stacked bar histograms reflecting $CCL2^+$, $MMP12^+$, and $eMyHC^+$ cell density inside (-1 to -50 µm) and outside (+1 to +200 µm) the necrotic lesion boundaries. Scale bars: 100 µm.

C. Five IF regions with inflammatory lesions in 2-mo D2.*mdx* + Q.W. Pred GAST samples. The MF subtypes and zones were visualized with IF for CCL2 (red; Zone A), MMP12 (yellow; Zone B), and eMyHC (green; Zone C). Note the unexpected collapse of RIZ organization in large inflammatory lesions with the overall diminished expression of MMP12 and the signal overlap of CCL2 with the remaining MMP12 (as observed previously by ST; **Fig. 7B-C**). Scale bars: macroscopic view 500 µm; insets 100 µm.

D. IF regions with inflammatory lesions in 2-mo D2.*mdx* samples. The presence of MF subtypes within the lesions was visualized with other ST predicted Zone B markers (**Figs. 7C-D**) CD18/ITGB2 (upper left panel; red), CD44 (upper right panel; red), and CD206 (middle panels; yellow). Mature MFs are marked with F4/80, pro-inflammatory monocytes/MFs with CCL2 and regenerating fibers with eMyHC. The middle right panel indicates the cell density quantification of $F4/80^+CD206^+$ and $F4/80^+CD206^-$ cells within

inflammatory lesions, placing the former closer to the center of the lesions and the latter closer to the periphery. Scale bars: 100 µm.

E. IF regions with inflammatory lesions enriched in damage-clearing zones but without advanced regeneration zones in 6-wks D2.*mdx* gastrocnemius muscles. These experiments are consistent and predictive of the architecture and organization of recently formed lesions, as revealed in the ST clustering analysis of age-matched samples (**Fig. S8E**). The MF subtypes and zones were visualized with IF for CCL2 (red; Zone A), MMP12 (yellow; Zone B), and eMyHC (green; Zone C). White rectangles indicate the region magnified in the middle panel. Scale bars: macroscopic view 500 µm; insets 100 µm.

F. IF regions with inflammatory lesions in 2-mo D2.*mdx* samples. The presence of MF subtypes within the lesions was visualized with another Zone B ST-predicted marker (ATF3; **Figs. 7C**). Pro-inflammatory monocytes/MFs were visualized with CCL2 and regenerating fibers with eMyHC. The right panel indicates the cell density quantification of $ATF3^+$ cells within inflammatory lesions in regions 2 and 4 (occupying Zone B), placing them adjacent to $CCL2^+$ cells (Zone A). Scale bars: 100 µm.

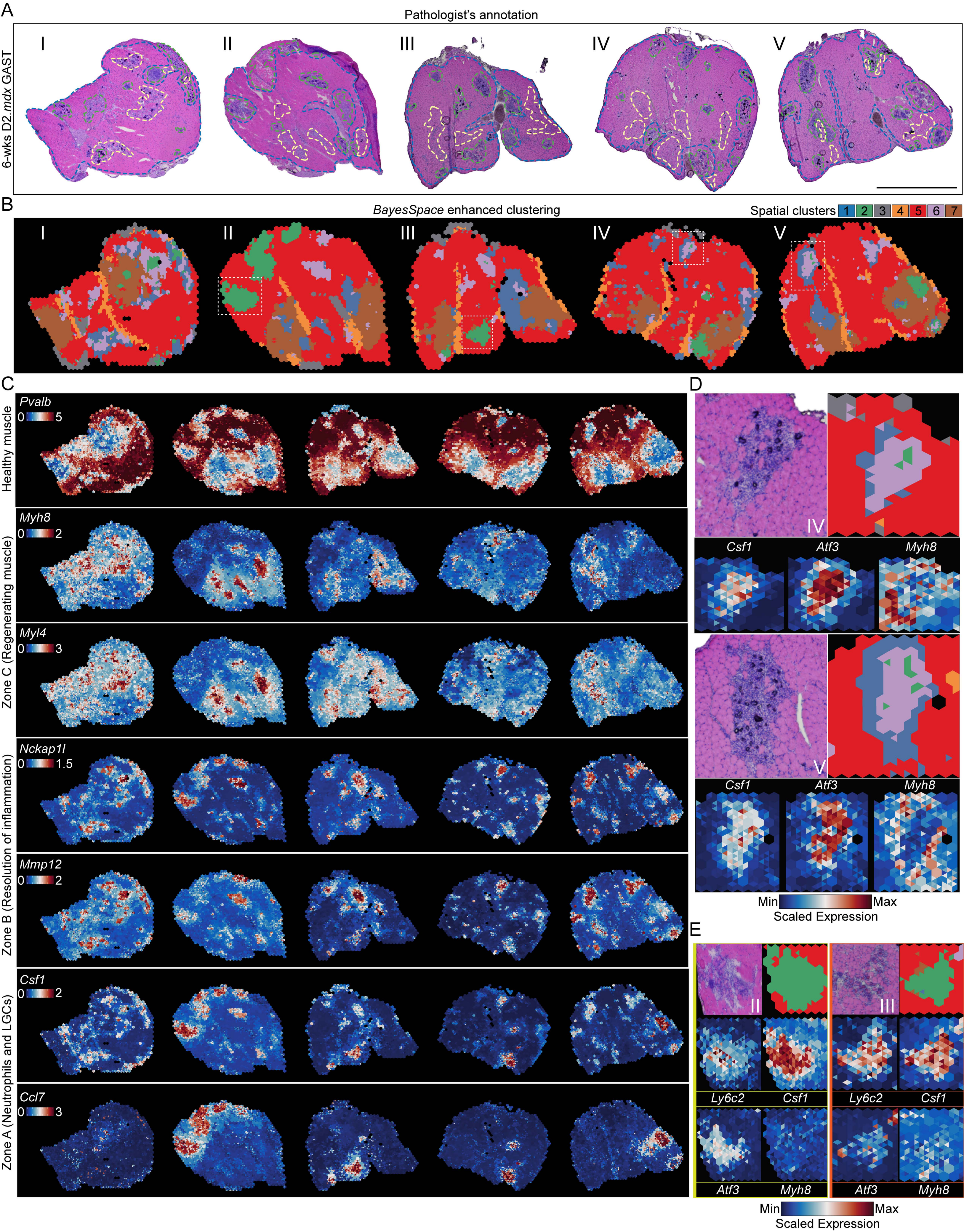

A
Pathologist's annotation
6-wks D2.mdx GAST
I
II
III
IV
V
B
BayesSpace enhanced clustering
Spatial clusters 1 2 3 4 5 6 7
C
Healthy muscle
Pvalb 0 5
Myh8 0 2
Myl4 0 3
Zone C (Regenerating muscle)
Nckap1l 0 1.5
Mmp12 0 2
Zone B (Resolution of inflammation)
Csf1 0 2
Ccl7 0 3
Zone A (Neutrophils and LGCs)
D
Csf1
Atf3
Myh8
Min Max
Scaled Expression
E
Ly6c2
Csf1
Atf3
Myh8
Min Max
Scaled Expression

Supplementary Figure S8 (related to main Figure 7).

A. H&E images of mouse gastrocnemius from 6-wks D2.*mdx* animals used for ST (6, 7). Histopathological annotation areas are noted (yellow: regenerative muscle; green: necrotic/inflammatory lesions; blue: healthy muscle) and classified as described in **Fig. 1A**. Latin numbers indicate the reference sample number. Each section is derived from a different biological replicate, and all datasets were integrated to remove any batch effects.

B. Enhanced subspot resolution clustering (*BayesSpace*) for the five samples indicated in panel **S8A**. The seven spatial clusters are color-coded, as in **Fig. 4B**. The white rectangles highlight areas with inflamed and structured regeneration zones.

C. The spatial expression of representative genes coding for damage-clearing and regenerative inflammation markers is shown. *Pvalb* indicates the healthy muscle (cluster 7), *Csf1* and *Ccl7* indicate the LGCs (cluster 2*), Mmp12* and *Nckap1l* indicate the presence of resolution-related MFs and GFEMs (cluster 6), and *Myh8* and *Myl4* indicate the newly regenerating fibers (cluster 1). Note the differential spatial expression patterns between the indicated markers and the regenerative architecture, as observed previously in **Figs. 4B-D**.

D. Magnified histological and *BayesSpace* clustering view of lesions in regions IV and V from panel **S8B**. The spatial expression of indicated RIZ markers in each of these regions is provided at the bottom panel. Note the expected RIZ organization and architecture as described previously in **Fig. 7**.

E. Magnified histological and *BayesSpace* clustering view of lesions in regions II and III from panel **S8B**. The spatial expression of indicated RIZ markers in each of these regions is provided at the bottom panel. Note the unique spatial clustering, with Zone B (spatial

cluster 6) and C (regenerating fibers; spatial cluster 1) being absent and Zone A (spatial cluster 2) strongly enriched in necrotic fibers, pointing towards recent lesion formation.

Supplemental Methods

Mice

This study used C57BL/6J (RRID:IMSR_JAX: 000664), and DBA/2J mice (RRID: IMSR_JAX: 000671) carrying a nonsense mutation in *Gpmnb* (D2.*Gpnmb*$^{-}$), the coisogenic strain DBA/2J-Gpnmb+/SjJ with a functional *Gpnmb* allele (D2.*Gpnmb*$^{+}$; RRID: IMSR_JAX: 007048) and *mdx* mice on the DBA/2J background (D2.B10-Dmd^{mdx}/J; RRID: IMSR_JAX: 013141) carrying the dystrophin gene mutation (D2.*mdx*). Mice were obtained from the Jackson Laboratories, bred under specific-pathogen-free conditions, with *ad libitum* access to food and water, and a 12-hour light cycle. Prednisolone suspensions were prepared in a cherry syrup vehicle for weekly *per os* (PO) treatment of 5 mg/kg dose (equates to a dose of 0.41 mg/kg in humans when normalized to the body surface area). Euthanasia was performed by CO_2 exposure per IACUC guidelines.

Acute sterile muscle injury

Mice (8-12 weeks-old) were anesthetized with isoflurane and 50 µl of 10 µM cardiotoxin (EMD Millipore, 217503-1MG) was injected into the TA muscle (8). Mice were monitored after recovery until they were euthanized. Muscles were recovered for flow cytometry analysis on days 1-4 post-injury or for histopathological assessments on days 4 to 8 post-injury.

Histological analysis of muscle regeneration

Muscles were removed, mounted on precut cork discs (EMS #63305) using tragacanth gum (MP Biomedicals, #104792), and snap-frozen in nitrogen-chilled isopentane (-160°C). 8 µm thick cryosections were cut (Leica CM1950) and stained with hematoxylin-eosin (H&E) as described previously (1). H&E-stained muscle sections were scanned with a Leica Aperio Versa digital slide scanner. For each histological analysis, at least six sections (per condition) were selected where the total regenerative region within the CTX injured TA muscle was at least 70%.

For each TA, myofibers in the entire injured area were analyzed and quantified with the *Muscle Fiber* 2.0 module of the HALO Digital Pathology software (Indica Labs). The regeneration area is expressed as the number of centrally nucleated fibers over the total area. For the ST sample selection and histological annotations, two active/experienced clinical pathologists at the Johns Hopkins All Children's Hospital specializing in muscle pathology performed and guided the assessments. Their evaluation of each injury/regeneration stage included a pool of 20 biological replicates per timepoint, from which we selected the samples that collectively incorporated most of the features the pathologists had identified to place on the Visium ST slides.

Immunofluorescence

Tibialis anterior and gastrocnemius muscles embedded in tragacanth gum were cryosectioned at 8 µm, fixed in ice-cold acetone for 5 min, blocked for 1 hour at room temperature in PBS containing 5% BSA, and stained for immunofluorescent (IF) analysis using a combination of Phalloidin (CF568-conjugated; Biotium #00044; 1:100), rabbit anti-laminin (Sigma-Aldrich L9393; 1:200), mouse anti-eMyHC (DSHB F1.652; 1:20), rabbit anti-MYH3 (Proteintech 22287-1-AP; 1:250), rabbit anti-Desmin (Abcam 32362; 1:200), rat anti-F4/80 (Abcam 6640; 1:200), rat anti-CD68 (Bio-Rad MCA1957GA; 1:100), goat anti-GPNMB (R&D Systems AF2330; 1:250), rat anti-CCL2 (Bio-Techne MAB479-SP; 1:100), rabbit anti-MMP-12 (Proteintech 22989-1-AP; 1:400), rabbit anti-GDF15 (Abcam ab105738; 1:100), rat anti-CD18/ITGB2 (Thermo Scientific 14-0181-82, 1:200), rat anti-CD44 (BD 550538; 1:100), rat anti-CD206-PE (BioLegend #141705; 1:50), rat anti-CD163-PE (BioLegend #155307; 1:50), rabbit anti-ATF3 (Novus Biologicals NBP1-85816; 1:250), and mouse anti-Ki67 (BD Pharmingen 550609; 1:20) primary antibodies for 1 hour at RT. Unconjugated primary antibody staining was followed by three 5-minute PBST washes and stained with donkey anti-rabbit Cy3 (JIR 711-165-152; 1:200), donkey anti-rat FITC (JIR 712-095-

153; 1:200), goat anti-rat Alexa Fluor 647 (Invitrogen A-21247; 1:200), donkey anti-goat Alexa Fluor 647 (Invitrogen A-21447; 1:200), donkey anti-mouse Alexa Fluor 488 (JIR 715-545-151; 1:200), and goat anti-mouse Alexa Fluor 488 (Invitrogen A-21121; 1:200) secondary antibodies for 45 min at RT. Following three PBST washes, the nuclei were counterstained with 1 µg/ml Hoechst 33342 for 1 minute, washed twice in PBS, and samples were mounted with Fluoromount (Sigma F4680). The IF slides were scanned at 20x and visualized with the dedicated fluorescent cameras of a Leica Aperio Versa digital slide scanner. Whole slide IF images were processed and analyzed for detection and co-localization of $CD68^+$/$Ki67^+$ cycling MFs, inflammation index ($CD68^+$ or $F4/80^+$ areas), as well as regenerating muscle fiber distribution ($eMyHC^+$ fibers) by a blinded investigator using the *Object Colocalization FL* 1.0 and *Area* Quantification *FL* 1.0 modules of the HALO software (Indica Labs). For quantifying the distance of the GFEMs ($CD68^+GPNMB^+$) and other MF subtypes ($CD68^+GPNMB^-$) to the regenerating fibers ($eMyHC^+$), the *Cytonuclear FL* 1.0 and *Spatial Analysis FL* 1.0 (Nearest Neighbor Analysis workflow) modules (HALO software; Indica Labs) were used. For calculating cell densities within and outside an interface layer (i.e., necrotic lesion), the *Cytonuclear FL 1.0* and *Infiltration Analysis* workflow (HALO software; Indica Labs) were used. Min-max normalization was then applied to scale the cell densities in each interface distance bin and plotted with *ggplot* as a 100 percent stacked bar. Representative high-resolution IF images were obtained under a confocal microscope with a resonant scanning disk (Nikon A1R, Nikon Instruments) with Z-sectioning (0.5 µm). The Nikon NIS-Elements AR Analysis 4.40 software was used to create the volume projection image (3-D reconstruction), and the final figures were assembled in Illustrator v27.2 (Adobe).

***In vivo* isolation of MFs from muscle**

Isolation of muscle-infiltrating MFs was performed as described previously (9, 10). Briefly, the fascia of the TA was removed, and muscles were dissociated in either RPMI containing 0.2% collagenase B (Roche Diagnostics GmbH) at 37°C for 1 hour or by using the MACS Skeletal Muscle Dissociation Kit (Miltenyi, 130-098-305) or gentleMACS Octo Dissociator, per kit instructions. Cell homogenate was filtered through a 100 µm and a 40 µm filter, and $CD45^+$ cells were isolated using magnetic sorting (Miltenyi Biotec). For FACS, myeloid cells were treated with Fcγ receptor blocking antibodies and with 10% normal rat serum: normal mouse serum 1:1 mix, then stained with a combination of PE-conjugated anti-Ly6C antibody (HK1.4, eBioscience), APC-conjugated or FITC-conjugated F4/80 antibody (BM8, eBioscience), FITC-conjugated Ly6G antibody (1A8, Biolegend), Pacific Blue-conjugated MHCII antibody (M5/114.15.2, Biolegend) and eFluor660-conjugated GPNMB antibody (CTSREVL, eBioscience). $Ly6C^{low}$ $F4/80^{high}$ $Gpnmb^-$ MFs, $Ly6C^{low}$ $F4/80^{high}$ $Gpnmb^+$ MFs, and $Ly6C^{low}$ $F4/80^{high}$ $MHCII^+$ MFs were quantified (gating strategy is shown in **Figs. S4A** and **S4G**). In each experiment, compared samples were processed in parallel to minimize experimental variation. Cells were analyzed on either a Cytoflex LX (Beckman Coulter), or MoFlo Astrios EQ (Beckman Coulter) sorter, and data analysis was performed using FlowJo V10 software.

Muscle-infiltrating MF cell culture for conditioned medium generation and apoptosis assay

Wild-type (C57BL/6J) $Ly6C^{low}$ $F4/80^{high}$ $Gpnmb^-$ MFs, and $Ly6C^{low}$ $F4/80^{high}$ $Gpnmb^+$ MFs were sorted from CTX-injured muscle at day 4. An equal number of sorted cells per population were seeded ($1x10^6$ cells per well) and cultured with DMEM containing 20% endotoxin-reduced fetal bovine serum (FBS) and 20% conditioned medium of L929 cell line (enriched in saturating levels of CSF-1; tested in a 5-day BMDM differentiation assay) for 12 hours (11, 12). The supernatant was then collected and centrifugated to obtain the MF-conditioned medium used in

myoblast proliferation and differentiation assays (see below). For the apoptosis assay, sorted MFs were seeded in coverslips, fixed with 4% PFA, and immunostained with Cleaved Caspase 3 (Cell Signaling #9661; 1:200) for 1 hour at RT. Immunofluorescent and brightfield images were obtained using a Carl Zeiss Axio Imager Z2 microscope and analyzed for detection of Cleaved Caspase 3$^+$ MFs by a blinded investigator using Fiji. Representative images and figures were then assembled in Illustrator v27.2 (Adobe).

Myoblast proliferation and differentiation assay

Murine myoblast C2C12 cells were obtained from American Type Culture Collection (CRL-1772) and were maintained according to the company's instructions. In brief, cells were cultured in DMEM supplemented with 10% FBS, 100 U/ml penicillin, and 100 μg/ml streptomycin (growth medium) at 37°C in 5% CO2 and 95% air at 100% humidity. For proliferation assays, C2C12 cells were seeded at 10,000 cells/cm^2 on Matrigel (1:10) and incubated for 1 day with the MF-conditioned medium plus 2.5% FBS (13, 14). For differentiation assays, C2C12 cells were seeded at 30,000 cells/cm^2 on Matrigel (1:10) and incubated for 3 days with the MF-conditioned medium plus 2% horse serum (13, 14). Cells were then fixed with 4% PFA, incubated with anti-Ki67 (Abcam #15580; 1:200), or anti-Myosin heavy chain 1E (DSHB MF 20; 1:20) for 1 h at room temperature, and were subsequently visualized using Cy3-conjugated secondary Abs (JIR; 1:200). The nuclei were counterstained with 0.1–1 μg/ml Hoechst. IF images were obtained using a Carl Zeiss Axio Imager Z2 microscope and analyzed for proliferation (% of Ki67$^+$ myoblasts) and fusion (% of myotubes with > 3 myonuclei) index using Fiji.

Spatial RNA sequencing library preparation

Fresh frozen skeletal muscle samples were cryosectioned (Leica CM1950) at 10-µm section thickness and were placed on the pre-chilled Optimization slides (Visium, 10X Genomics, PN-1000191) to determine the optimal lysis time. The tissues were treated as recommended by 10X Genomics, and the optimization procedure showed an optimal permeabilization time of 30 min of digestion and release of RNA from the tissue slide. Spatial gene expression slides (Visium, 10X Genomics, PN-1000185) were used for ST following the Visium User Guides, and whole slide images were taken using a 20X objective of a Leica Aperio Versa scanner. Next-generation sequencing libraries were prepared according to the Visium user guide. Libraries were loaded at 300 pM and sequenced on a NovaSeq 6000 System (Illumina) as recommended by 10X Genomics. Each section is derived from a different biological replicate, and each library was obtained from a separate Visium experiment followed by bioinformatic integration to remove any batch effects.

Spatial transcriptomics data analysis

Filtered feature-barcode expression matrices from *SpaceRanger* (v1.3.1) were used as initial input for the spot-level ST analysis using *Giotto (v2.0.0).* Spots with less than 25 measured genes were filtered out. Ribosomal, mitochondrial, *Rik*, and *Gm* genes were excluded from the analysis. Principal component analysis was run on the highly variable genes calculated by *calculateHVF* function. PCA coordinates were used for data integration with *harmony* (v0.1.1). UMAP dimension reduction was performed with harmony embeddings and visualized in R. Spot-level clustering was performed using the Leiden algorithm at resolution 0.3. Spatially variable genes were calculated with *binSpect* using log-normalized data (FDR < 0.001). Gene Ontology enrichment analysis was performed using PANTHER (15) and Metascape v3.5 (16). Filtered gene-spot matrix and metadata were further analyzed with *BayesSpace* (v1.6.0) using the top 10 PCs

and the top 2000 HVGs. Clusters were then created at sub-spot resolution with the following parameters (d=10, platform=”Visium”, init.method=”mclust”, model=”t”, gamma=2). Optimal cluster numbers were determined by *qTune* function and manual curation of marker genes. Cluster marker genes were determined using *immunogenomics/presto* (v1.0.0) (padj<0.05, logFC>0.25). Cell-type compositions were calculated for each spot using *cell2location* (v0.1) (17). Reference expression signatures of major cell types were estimated using regularized negative binomial regressions and our integrated single-cell RNA-seq datasets. Each slide was later deconvoluted using hierarchical Bayesian models as implemented in *run_cell2location* function. For cell-type-specific abundance estimations, we used 10 cells per location with a detection alpha of 20 as hyperparameters. The donut charts were created using the *ggpubr* package. To further investigate the spatial organization of the tissue zones and calculate the observed vs. expected ratio, we have developed an R shiny app (*SpatialZoneR*) to interactively define and analyze tissue zones in sub-spot resolution using Visium ST rds data. Gene set expression values representing different cell subtypes at each subspot were extracted and binarized (expression cutoff was selected at > q90 for each marker gene) to define tissue zone cell organization. *SpatialZoneR* is made available as a fully open-source tool at https://github.com/hlszlaszlo/SpatialZoneR.

Single-cell RNA-sequencing library preparation

After tissue digestion and bead selection, CD45$^+$ single-cell sorted suspensions were washed and resuspended in 0.04% BSA in PBS at a concentration of at least 400 cells/µL. Cells were counted manually with a hemocytometer to determine their concentration. Single-cell RNA-sequencing libraries were then prepared using the Chromium Single-Cell 3’ reagent kit v3.1 (10X Genomics, Pleasanton, CA) in accordance with the manufacturer’s protocol. Briefly, the cells were diluted into the Chromium Single-Cell A Chip to yield recovery of ~10,000 single-cell

transcriptomes with < 5% doublet rate. Following the library preparation, the libraries were sequenced on the NovaSeq 6000 sequencer (Illumina, San Diego, CA) to produce about 450 million reads per library and, on average, a minimum of 40,000 reads per single cell.

Single-cell RNA-seq data analysis

Single-cell sequencing reads were processed and aligned to the mouse reference transcriptome (*mm10*) with the *CellRanger* (v7.0.1) (10x Genomics, Pleasanton, CA). We used *CellBender* (v0.2.2) to eliminate technical artifacts. From the gene expression matrix, the downstream analysis was carried out in R (v4.2.1). Quality control, filtering, data clustering and visualization, and the differential expression analysis were carried out using *Seurat* (v4.1.3) R package (18) with custom modifications. Genes expressed in <10 cells and cells with <500 detected genes were removed from the gene expression matrix. Cells with high mitochondrial mapped read percentage (determined by MiQC) (19), as well as outliers with UMI counts in the lower and upper 2.5% (q97.5) were excluded from downstream analysis. Doublets identified using *scDblFinder* (v1.10.0) were also removed. Ribosomal, mitochondrial, Rik and Gm genes were excluded from the analysis. After log-normalizing the data, the expression of each gene was scaled, and PCA was performed on the top feature genes determined by *DUBStepR* (v1.2.0). Data integration was carried out using *harmony* (20). Harmony embeddings were used for dimension reduction, clustering, and visualization. Unsupervised shared nearest neighbor (SNN) clustering was performed with a k parameter of 20 using the Leiden algorithm, and visualization was done using PaCMAP (21) using the *ReductionWrappers* (v2.5.4) R package. Cell type automatic annotations were predicted using the *SingleR* (v1.10.0) package and the *ImmGen* database from *celldex* as reference (22, 23). Confidence scores were visualized using the *plotScoreHeatmap* function from SingleR. We also verified the accuracy of the assignment by exploring the

expression of known cell-type gene markers and by evaluating the top differential genes between cell clusters on *PanglaoDB* (24). Cluster marker genes were identified using *presto* to perform a Wilcoxon rank-sum test and auROC analysis (logFC > 0.25, p-adj < 0.0001, AUC > 0.65, and ranked based on their exclusive presence in each cluster calculated as the difference between the percentage of inclusion vs. exclusion). Cells in the macro-clusters of interest (Monocytes, MFs, and DCs) were extracted and reanalyzed. Visualization was done using t-distributed stochastic neighbor embedding (t-SNE) to reveal local differences (25). To determine optimal cluster resolution, we first utilized a subsampling-based approach *chooseR* that guides parameter selection while characterizing cluster robustness (26) (**Figs. S2E-F**). We also independently performed the clustering workflow with the *FindClusters* function from resolution 0.1 to 1 in steps of 0.1 (**Fig. S3B**). The resolution 0.6 was evaluated as best by both approaches. However, one cluster emerged that expressed marker genes representing multiple cell populations (**Figs. 2D** and **S3A-C**), suggesting re-clustering/splitting into two clusters. This choice was evaluated and confirmed with the aid of the *Clustree* package (**Fig. S3B**) for a total of 10 clusters. To assign identities to these immune subclusters, we manually curated and cross-referenced their marker genes with known subtype markers data from the literature and relevant mouse models and studies (**Fig. 5**) (5, 27-30). For computing the Spearman similarity correlation between the subpopulations in the different samples, the R package *corrplot* was used (logFC > 0.5, p-adj < 0.1, auc > 0.5, sig.level = 0.05, and AOE order) (31). Feature plots were generated using the *Nebulosa* package (32), heatmaps using the *pheatmap* (v1.0.12) package, *tricycle* (v1.4.0) for cell cycle stage scoring (33), and *ggalluvial* (v0.12.3)(34) for compositional plots. Combined feature expression module scores were calculated using *Seurat*'s *AddModuleScore* function. Lastly, the dynamic changes in gene expression were evaluated by performing a trajectory analysis using three packages (*Slingshot, Monocle v2,* and *scVelo*) (4, 35-37). To give a finer definition of cell states and

unknown myeloid subpopulations, the trajectory analyses were performed only on the MF, monocyte, and DC subsets. RNA velocity estimations and visualizations were performed using dynamic modeling by *scvelo (4, 38)*. Counts were filtered to a minimum of 20 shared counts across samples, and the top 2000 variable genes were selected for velocity analyses. Fifty nearest neighbors were used when calculating moments of velocity, followed by velocity estimation and embedding on dimensional reductions using scvelo's *velocity_embedding_stream* function. Pseudotime analysis was performed using *Slingshot* and the *harmony*-corrected principal components to calculate cluster lineages with *getLineages* providing cluster 3 (circulating monocytes) as a start cluster. Pseudotime trajectories were then calculated using the t-SNE embeddings and visualized appropriately. RNA velocity-informed embedding was used to represent the proliferating MF cluster in isolation using the *veloviz* package (39). The cell cycle phase (G1, S, G2M) of individual cells was assigned using the calculated standardized z-scores of highly expressed stage-specific cell cycle marker genes using a previously defined list of cell cycle genes (40, 41). *tricycle* was then used to visualize the cell cycle stage scoring of the clusters as Cartesian coordinates by computing the kernel density of θ conditioned on a phenotype using the von Mises distribution (33). To generate the TF heatmap, the average log-normalized expression values for the list of TFs were calculated, ordered, and decile-filtered to select the top 75% for visualization.

Human single-nuclei RNA-seq data analysis

Raw sequence data for human samples (vastus lateralis biopsies from healthy and DMD patients) from BioProject PRJNA772047 (5) were processed using *CellRanger (v7.0.1)* to generate gene expression matrices aligned to the *hg38* reference genome. Single nuclei data were logged normalized, and scaled with *Seurat*. Doublets identified using *scDblFinder*, nuclei with < 500 UMI,

and high mitochondrial gene content (*miQC*) were removed before integrating datasets using *harmony*. Major cell-type populations were identified using the *SingleR* package and the Human Primary Cell Atlas, and only cells classified as “Monocyte,” and “Macrophage” were selected for downstream analysis (22). Clustering trees were used to identify the optimal number of clusters (cluster resolution = 0.9) and visualized with t-SNE. For each immune subset, marker genes were identified using *presto* using the same parameters as described above for the scRNA-seq datasets. Feature plots were generated using the *Nebulosa* package (32) and *tricycle* for cell cycle stage scoring of individual cells, as described previously (33).

ATF3 KO microarray gene expression data analysis

GEOquery and *limma* v 3.58.1 were used to perform differential expression analysis using the original submitter-supplied processed data tables as input and by applying multiple-testing corrections (Benjamini & Hochberg FDR) on P-values to correct for the occurrence of false positives (p-value cutoff of 0.05).

Chromatin immunoprecipitation (ChIP)

ChIP was performed as previously described (42) with minor modifications. Briefly, sorted cells were crosslinked with DSG (Sigma) for 30 minutes and then with formaldehyde (Sigma) for 10 minutes. After fixation, chromatin was sonicated with Diagenode Bioruptor to generate 200-1000 bp fragments. Chromatin was immunoprecipitated with an antibody against H3K27ac (ab4729). Chromatin antibody complexes were precipitated with Protein A-coated paramagnetic beads (Life Technologies). After six washing steps, complexes were eluted and reverse crosslinked. DNA fragments were column purified (Qiagen, MinElute). The amount of immunoprecipitated DNA was quantified with Qubit fluorometer (Invitrogen). Libraries were

prepared by Ovation Ultralow Library Systems (Nugen) from two biological replicates according to the manufacturer's instructions.

ChIP-seq analysis

The collection of TF ChIP-seq data and the primary analysis of raw sequence reads were carried out as described earlier (43, 44). In more detail, raw data were selected and downloaded from the SRA database of NCBI. Alignment to the *mm10* mouse reference genome assembly was performed by the *BWA* v0.7.17 tool (45). BAM files were created by *SAMtools* v1.7 (46). Genome coverage (bedgraph) files were generated by *makeUCSCfile.pl* (HOMER v4.9.1) (47) and visualized by Integrative Genomics Viewer (IGV v2.16.1) (48).

Assay for Transposase-Accessible Chromatin with high-throughput sequencing (ATAC-seq)

ATAC-seq was carried out as described earlier with minor modifications (49). 20,000 $Ly6C^{high}$ circulating cells from C57BL/6J were sorted in ice-cold PBS. Nuclei were isolated with ATAC-Lysis Buffer (10mM Tris-HCl pH7.4, 10mM NaCl, 3mM $MgCl_2$, 0.1% IGEPAL) and were used for tagmentation using Nextera DNA Library Preparation Kit (Illumina). After tagmentation DNA was purified with MinElute PCR Purification Kit (Qiagen). Tagmented DNA was amplified with Kapa Hifi Hot Start Kit (Kapa Biosystems) using 9 PCR cycles. Amplified libraries were purified again with MinElute PCR Purification Kit. Fragment distribution of libraries was assessed with Agilent Bioanalyzer and libraries were sequenced on a HiSeq 2500 platform.

Mapping and normalization of ATAC-seq

Circulating monocytes ($Ly6C^{high}$) and muscle-derived $Ly6C^{high}$ MFs of day 1 and $Ly6C^{low}$ MFs of day 4 upon muscle injury datasets were used. The primary analysis of ATAC-seq-derived raw

sequence reads has been carried out using the newest version of the ChIP-seq analysis command line pipeline, including the following steps: Alignment to the *mm10* mouse genome assembly was done by the BWA tool (45), and BAM files were created by *SAMTools* (46). Signals (peaks) were predicted by MACS2 (50), artifacts were removed according to the blacklist of ENCODE (51), and filtered for further analysis by removing low mapping quality reads (MAPQ score < 10), duplicated reads and reads located in blacklisted regions. All regions derived from at least any two samples were united within 0.5kb and those summits having the highest MACS2 peak score in any sample were assigned to each region. Promoter-distal regions were selected, excluding the TSS+/-0.5kb regions according to the mouse GRCm38.p1 (*mm10*) annotation version. Tag directories used by HOMER in the following steps were generated with a 120-nucleotide fragment length with makeTagDirectory (47). Genome coverage (bedgraph and tdf) files were generated by makeUCSCfile.pl (HOMER) and igvtools, respectively, and used for visualization with IGV2 (48). Coverage values were further normalized by the upper decile value detected in the consensus regions for each sample to minimize the inter-sample variance.

Motif enrichment analysis

Peaks derived from day 4 Ly6C^{low} muscle MFs and closer than 50 kb to any TSS of *Gpnmb* and other GFEM marker genes showing an expression dynamic similar to *Gpnmb* (Pearson correlation > 0.8) were determined by *intersectBed* (*bedtools* v2.27.1) (52) and used as inputs for a *de novo* motif enrichment analysis. The central 200 bp of the peaks were used as target sequences, and the enrichment of 10-, 12-, and 14-mers was determined by *findMotifsGenome.pl* (HOMER). P-values were calculated by comparing the number of target and random (background) sequences carrying a certain motif. To generate the motif lolliplot, we used *trackViewer* (53) to visualize individual motif prediction scores for selected DNA sequences.

Chromatin interactions of the -23kb enhancer of the Gpnmb gene locus

We applied a capture Hi-C (cHi-C) approach (54) to map chromatin interactions in the murine *Gpnmb* gene locus (chr6:49007000-49060000, mm10). Briefly, differentiated mouse BMDMs were cross-linked with 1% formaldehyde and stored as cell pellets at -20°C. Cells were lysed and processed using the Arima Hi-C kit according to the manufacturer's protocol. A capture enrichment step was applied to the generated Hi-C libraries using custom-designed probes (SureSelect, Agilent Tier 2 -2,9Mb) targeting a selective group of myeloid-specific genomic regulatory elements, including the -23kb enhancer of the *Gpnmb* gene locus shown in **Fig. 8C**. cHi-C libraries were prepared with the Arima Hi-C kit and Agilent SureSelect library systems according to the manufacturers's protocol. cHi-C raw sequence output was processed with *Hicup* (v0.6.1) to produce a filtered set of mapped interaction pairs in the mm10 genome. Interactions between virtual restriction fragments were detected using *Chicago* (v1.6.0) and custom weights calculated from high-confidence interactions in the data. Bigwig files containing mapped normalized interaction pairs were uploaded to the IGV browser to visualize chromatin loops of the -23kb enhancer of the *Gpnmb* gene locus together with ChIP-seq tracks.

References

1. Patsalos A, et al. A growth factor-expressing macrophage subpopulation orchestrates regenerative inflammation via GDF-15. *J Exp Med.* 2022;219(1).

2. Kiselev VY, et al. SC3: consensus clustering of single-cell RNA-seq data. *Nat Methods.* 2017;14(5):483-6.

3. Zappia L, and Oshlack A. Clustering trees: a visualization for evaluating clusterings at multiple resolutions. *Gigascience.* 2018;7(7).

4. Bergen V, et al. Generalizing RNA velocity to transient cell states through dynamical modeling. *Nat Biotechnol.* 2020;38(12):1408-14.

5. Scripture-Adams DD, et al. Single nuclei transcriptomics of muscle reveals intra-muscular cell dynamics linked to dystrophin loss and rescue. *Commun Biol.* 2022;5(1):989.

6. Stec MJ, et al. A cellular and molecular spatial atlas of dystrophic muscle. *Proc Natl Acad Sci U S A.* 2023;120(29):e2221249120.

7. Coulis G, et al. Single-cell and spatial transcriptomics identify a macrophage population associated with skeletal muscle fibrosis. *Sci Adv.* 2023;9(27):eadd9984.

8. Hardy D, et al. Comparative Study of Injury Models for Studying Muscle Regeneration in Mice. *PLoS One.* 2016;11(1):e0147198.

9. Giannakis N, et al. Dynamic changes to lipid mediators support transitions among macrophage subtypes during muscle regeneration. *Nat Immunol.* 2019;20(5):626-36.

10. Patsalos A, et al. The BACH1-HMOX1 Regulatory Axis Is Indispensable for Proper Macrophage Subtype Specification and Skeletal Muscle Regeneration. *J Immunol.* 2019;203(6):1532-47.

11. Barish GD, et al. A Nuclear Receptor Atlas: macrophage activation. *Mol Endocrinol.* 2005;19(10):2466-77.

12. Heap RE, et al. Proteomics characterisation of the L929 cell supernatant and its role in BMDM differentiation. *Life Sci Alliance.* 2021;4(6).

13. Saclier M, et al. Effects of Macrophage Conditioned-Medium on Murine and Human Muscle Cells: Analysis of Proliferation, Differentiation, and Fusion. *Methods Mol Biol.* 2017;1556:317-27.

14. Varga T, et al. Macrophage PPARγ, a Lipid Activated Transcription Factor Controls the Growth Factor GDF3 and Skeletal Muscle Regeneration. *Immunity.* 2016;45(5):1038-51.

15. Mi H, et al. Large-scale gene function analysis with the PANTHER classification system. *Nat Protoc.* 2013;8(8):1551-66.

16. Zhou Y, et al. Metascape provides a biologist-oriented resource for the analysis of systems-level datasets. *Nat Commun.* 2019;10(1):1523.

17. Kleshchevnikov V, et al. Cell2location maps fine-grained cell types in spatial transcriptomics. *Nat Biotechnol.* 2022;40(5):661-71.

18. Butler A, et al. Integrating single-cell transcriptomic data across different conditions, technologies, and species. *Nat Biotechnol.* 2018;36(5):411-20.

19. Hippen AA, et al. miQC: An adaptive probabilistic framework for quality control of single-cell RNA-sequencing data. *PLoS Comput Biol.* 2021;17(8):e1009290.

20. Korsunsky I, et al. Fast, sensitive and accurate integration of single-cell data with Harmony. *Nat Methods.* 2019;16(12):1289-96.

21. Wang Y, et al. Understanding How Dimension Reduction Tools Work: An Empirical Approach to Deciphering t-SNE, UMAP, TriMAP, and PaCMAP for Data Visualization. *arXiv pre-print server.* 2020.

22. Aran D, et al. Reference-based analysis of lung single-cell sequencing reveals a transitional profibrotic macrophage. *Nat Immunol.* 2019;20(2):163-72.

23. Heng TS, et al. The Immunological Genome Project: networks of gene expression in immune cells. *Nat Immunol.* 2008;9(10):1091-4.

24. Franzén O, et al. PanglaoDB: a web server for exploration of mouse and human single-cell RNA sequencing data. *Database.* 2019;2019.

25. Becht E, et al. Dimensionality reduction for visualizing single-cell data using UMAP. *Nat Biotechnol.* 2018.

26. Patterson-Cross RB, et al. Selecting single cell clustering parameter values using subsampling-based robustness metrics. *BMC Bioinformatics.* 2021;22(1):39.

27. De Micheli AJ, et al. Single-Cell Analysis of the Muscle Stem Cell Hierarchy Identifies Heterotypic Communication Signals Involved in Skeletal Muscle Regeneration. *Cell Rep.* 2020;30(10):3583-95 e5.

28. Giordani L, et al. High-Dimensional Single-Cell Cartography Reveals Novel Skeletal Muscle-Resident Cell Populations. *Mol Cell.* 2019;74(3):609-21 e6.

29. McKellar DW, et al. Large-scale integration of single-cell transcriptomic data captures transitional progenitor states in mouse skeletal muscle regeneration. *Commun Biol.* 2021;4(1):1280.

30. Dell'Orso S, et al. Single cell analysis of adult mouse skeletal muscle stem cells in homeostatic and regenerative conditions. *Development.* 2019;146(12).

31. Friendly M. Corrgrams: Exploratory displays for correlation matrices. *Am Stat.* 2002;56(4):316-24.

32. Alquicira-Hernandez J, and Powell JE. Nebulosa recovers single cell gene expression signals by kernel density estimation. *bioRxiv.* 2020:2020.09.29.315879.

33. Zheng SC, et al. Universal prediction of cell-cycle position using transfer learning. *Genome Biology.* 2022;23(1).

34. Brunson J. ggalluvial: Layered Grammar for Alluvial Plots. *Journal of Open Source Software.* 2020;5(49).

35. Qiu X, et al. Reversed graph embedding resolves complex single-cell trajectories. *Nat Methods.* 2017;14(10):979-82.

36. Street K, et al. Slingshot: cell lineage and pseudotime inference for single-cell transcriptomics. *BMC Genomics.* 2018;19(1):477.

37. Saelens W, et al. A comparison of single-cell trajectory inference methods. *Nat Biotechnol.* 2019;37(5):547-54.

38. Bergen V, et al. RNA velocity-current challenges and future perspectives. *Mol Syst Biol.* 2021;17(8):e10282.

39. Atta L, et al. VeloViz: RNA velocity-informed embeddings for visualizing cellular trajectories. *Bioinformatics.* 2022;38(2):391-6.

40. Tirosh I, et al. Dissecting the multicellular ecosystem of metastatic melanoma by single-cell RNA-seq. *Science.* 2016;352(6282):189-96.

41. Schwabe D, et al. The transcriptome dynamics of single cells during the cell cycle. *Mol Syst Biol.* 2020;16(11):e9946.

42. Daniel B, et al. Mapping the genomic binding sites of the activated retinoid X receptor in murine bone marrow-derived macrophages using chromatin immunoprecipitation sequencing. *Methods Mol Biol.* 2014;1204:15-24.

43. Nagy G, et al. Lineage-determining transcription factor-driven promoters regulate cell type-specific macrophage gene expression. *Nucleic Acids Res.* 2024.

44. Barta E. Command line analysis of ChIP-seq results. *2011.* 2011;17(1):5.

45. Li H, and Durbin R. Fast and accurate short read alignment with Burrows-Wheeler transform. *Bioinformatics.* 2009;25(14):1754-60.

46. Li H, et al. The Sequence Alignment/Map format and SAMtools. *Bioinformatics.* 2009;25(16):2078-9.

47. Heinz S, et al. Simple combinations of lineage-determining transcription factors prime cis-regulatory elements required for macrophage and B cell identities. *Mol Cell.* 2010;38(4):576-89.

48. Thorvaldsdottir H, et al. Integrative Genomics Viewer (IGV): high-performance genomics data visualization and exploration. *Brief Bioinform.* 2013;14(2):178-92.

49. Buenrostro JD, et al. Transposition of native chromatin for fast and sensitive epigenomic profiling of open chromatin, DNA-binding proteins and nucleosome position. *Nat Methods.* 2013;10(12):1213-8.

50. Zhang Y, et al. Model-based analysis of ChIP-Seq (MACS). *Genome Biol.* 2008;9(9):R137.

51. Consortium EP. An integrated encyclopedia of DNA elements in the human genome. *Nature.* 2012;489(7414):57-74.

52. Quinlan AR, and Hall IM. BEDTools: a flexible suite of utilities for comparing genomic features. *Bioinformatics.* 2010;26(6):841-2.

53. Ou J, and Zhu LJ. trackViewer: a Bioconductor package for interactive and integrative visualization of multi-omics data. *Nat Methods.* 2019;16(6):453-4.

54. Orlando G, et al. Capture Hi-C Library Generation and Analysis to Detect Chromatin Interactions. *Curr Protoc Hum Genet.* 2018;98(1):e63.
